# Noncanonical enhancers control gene expression and cell fate in human pluripotent stem cells

**DOI:** 10.1126/sciadv.adz3328

**Published:** 2026-11-13

**Authors:** Yang Cao, Oleksandr Dovgusha, Ayyub Ebrahimi, Carmela Rieline V. Cruz, Stephen Bevan, Lukas Widmer, Fereshteh Torabi, Julia Kurlovich, Ignacio Rodríguez-Polo, Soumen Khan, Manthan Patel, Oliver T. Shiels, Madapura M. Pradeepa, Sean M. Flynn, Ufuk Günesdogan, Stefan Schoenfelder

**Affiliations:** ^1^ Epigenetics Program, Babraham Institute, Babraham Research Campus, Cambridge CB22 3AT, UK.; ^2^ Göttingen Center for Molecular Biology, Department of Developmental Biology, University of Göttingen, Justus-von-Liebig Weg 11, 37077 Göttingen, Germany.; ^3^ Institute for Biomedical Research, Health and Medical University, Dalbergsweg 5, 99084 Erfurt, Germany.; ^4^ Blizard Institute, Barts and the London School of Medicine and Dentistry, The Blizard Building 4, Newark Street, London E1 2AT, UK.; ^5^ Department of Clinical and Biomedical Sciences, Faculty of Health and Life Sciences, University of Exeter, Exeter EX2 5DW, UK.; ^6^ Department of Molecular Developmental Biology, Max-Planck-Institute for Multidisciplinary Sciences, Am Faßberg 11, 37077 Göttingen, Germany.

## Abstract

Enhancers are key gene regulatory elements that ensure the precise spatiotemporal execution of developmental gene expression programs. However, recent findings indicate that current approaches to identify enhancers may not capture the full repertoire of active enhancers in mammalian genomes. Here, we combine massively parallel enhancer assays with chromatin structure and transcriptome profiling to functionally annotate enhancers genome-wide in human induced pluripotent stem cells. We find that a substantial fraction (∼40%) of accessible chromatin regions with enhancer function lack key features associated with active enhancers, including the active enhancer mark histone H3 lysine-27 acetylation and enhancer-associated RNAs. Perturbation of this class of noncanonical enhancers by CRISPR-mediated epigenome editing or deletion results in decreased levels of target gene expression and, in one instance, loss of pluripotent stem cell characteristics. Collectively, our data demonstrate enhancer activity for a class of gene regulatory elements that had, until now, only been associated with a neutral or inactive status, challenging current models of enhancer function.

## INTRODUCTION

Developmental gene expression programs are controlled by transcriptional enhancers (*1*, *2*), often over large genomic distances from their target genes (*3*, *4*). The human genome is estimated to harbor up to 1 million mostly tissue-specific enhancers, vastly outnumbering genes (*5*). Highlighting their importance in development, aberrant enhancer function, or ectopic enhancer activation can lead to severe developmental malformations (*6*–*8*). Furthermore, thousands of disease-associated noncoding loci across dozens of disease indications carry hallmarks of enhancers (*9*), and an increasing number of human diseases are associated with impaired enhancer activity (*10*, *11*).

Enhancers also act as key regulators of the pluripotent stem cell (PSC) fate. Interfering with the function of enhancers that control key pluripotency genes results in the loss of mouse PSC properties (*12*). Modulating the frequency of enhancer-promoter interactions is a key gene regulatory step during stem cell maintenance and differentiation into specialized cell types (*13*, *14*). Epigenetic remodeling of enhancers has been shown to trigger reprogramming toward induced PSCs (iPSCs) (*15*), and targeting enhancers that control lineage-specifying genes can enhance the efficiency of differentiation from human PSCs (hPSCs) into target cell types (*16*). This illustrates the potential to experimentally target enhancers to direct cell fate decisions for stem cell–based applications in biomedicine.

Enhancers are predominantly located in the noncoding genome and are characterized by a high density of transcription factor binding sites. Enhancers were originally found through their ability to drive the expression of reporter genes (*17*). However, most current genome-wide enhancer mapping strategies rely on transcription factor binding, such as EP300 (*18*), enrichment for specific posttranslational histone modifications (*19*–*21*), and/or the presence of enhancer-associated RNAs (eRNAs) (*22*). In particular, specific combinations of posttranslational histone tail modifications are widely used to classify enhancers into functional categories: inactive/neutral/primed [histone H3 lysine-4 monomethylation (H3K4me1)], poised [H3K4me1 and histone H3 lysine-27 trimethylation (H3K27me3)], and active [H3K4me1 and histone H3 lysine-27 acetylation (H3K27ac)] (*19*–*21*). Among these, H3K27ac is generally accepted as an active enhancer mark. However, recent findings suggest that not all, and possibly only a minority of, H3K27ac-enriched regions in the noncoding genome function as active enhancers. Only 12% of putative enhancers marked with H3K27ac in hPSCs function as transcriptional enhancers in a reporter gene assay (*23*). Deletion of selected enhancers with reporter gene activity, but not of those without, affects the transcription levels of target genes, irrespective of H3K27ac enrichment (*23*). Furthermore, a substantial fraction (∼50%) of H3K27ac-positive and EP300-bound regions do not show enhancer activity in vivo (*24*–*27*). Notably, H3K27ac is dispensable for enhancer activity in mouse embryonic stem cells (ESCs) (*28*, *29*). Conversely, genomic regions devoid of H3K27ac have been shown to act as enhancers. In mouse ESCs, noncanonical enhancers that lack H3K27ac but instead are enriched for acetylation in the histone H3 core domain have been identified (*30*). Studies dissecting gene control mechanisms at selected genomic loci in mouse (*Zfp42*, *Nanog*, *Rpp25*, and *Tdgf1*) and human (*POU5F1*) PSCs have uncovered functional gene regulatory elements that lack canonical enhancer features (*31*, *32*). Furthermore, around one-quarter of enhancers that are active in vivo in two mouse developmental regulator loci (*Gli3* and *Smad4/Smad6*) do not show canonical enhancer chromatin signatures in the corresponding tissues (*26*). Together, these studies challenge the concept of H3K27ac as a universal mark of active enhancers and raise the possibility that enhancer-like noncoding gene control elements exist in mammalian genomes that lack canonical enhancer chromatin signatures and thus evade detection by conventional chromatin profiling approaches. However, this has not been systematically addressed on a genome-wide scale in hPSCs.

Here, we combine a range of functional genomics methods including self-transcribing active regulatory region sequencing [STARR-seq (*33*)], assay for transposase-accessible chromatin with high-throughput sequencing [ATAC-seq (*34*)], gene expression profiling by RNA sequencing (RNA-seq) and transient transcriptome sequencing (TT-seq) (*35*), CUT&Tag (*36*) for posttranslational histone modifications, and Promoter Capture Hi-C (PCHi-C) (*37*, *38*) to functionally annotate enhancers in human iPSCs (hiPSCs) genome-wide. Unexpectedly, we find that a substantial proportion of genomic regions with enhancer function in hiPSCs are devoid of the canonical active enhancer mark H3K27ac and have only low levels of eRNAs, thus escaping detection by conventional enhancer mapping approaches. We identify naturally occurring genetic variants in hiPSC cell lines from different donors at these putative noncanonical enhancers that impair their activity, which occur concomitantly with changes in chromatin accessibility and nearby gene expression. This establishes a link between noncanonical enhancer genetic variation, chromatin structure, enhancer activity, and gene transcription. Furthermore, we show that perturbation of putative noncanonical enhancer function at their endogenous chromatin loci by CRISPR interference (CRISPRi) or enhancer deletion leads to a reduction in the expression levels of target genes, demonstrating enhancer-like gene regulatory potential. In particular, we identified a noncanonical enhancer without active enhancer features that is required for cell survival and maintenance of pluripotency. Last, noncoding putative regulatory elements with similar features also exist in mouse PSCs and in human and *Drosophila* cell lines. Together, our results challenge the concept of H3K27ac as a universal active enhancer mark and define a novel class of noncanonical enhancers in hPSCs, suggesting that eukaryotic genomes may harbor tens of thousands of hitherto unknown enhancers that actively contribute to the control of developmental gene expression programs.

## RESULTS

### Functional enhancer annotation in hiPSCs

To functionally annotate enhancers in hiPSCs, we performed genome-wide STARR-seq (*39*). To this end, genomic DNA was sonicated and cloned into a STARR-seq vector downstream of a minimal promoter that is inducible by human enhancers (*40*) and upstream of the 3′-untranslated region of a reporter gene. Sequencing of the input genome-wide STARR-seq library revealed 89% coverage of the mappable human genome, with a mean coverage of 5.9×. After transfecting the STARR-seq library into hiPSCs, we determined enhancer activity by RNA-seq. We generated three biological STARR-seq replicates from a hiPSC line generated by the HipSci consortium (*41*), which we specifically selected for its representative pluripotency and novelty score (fig. S1A) (*42*) and the absence of copy number variations (fig. S1B). The three hiPSC STARR-seq replicates showed high correlation (fig. S1, C and D), allowing us to pool sequence reads for subsequent analyses.

Using a bespoke algorithm to correct for potential biases in STARR-seq data (*43*), we identified 57,784 peaks in hiPSCs, 38,847 (67.3%) of which were putative enhancer candidates as they were located in intronic and intergenic regions of the human genome. To correlate STARR-seq activity with chromatin accessibility at the endogenous enhancer loci, we generated omni-ATAC-seq (*44*) libraries from hiPSCs. We identified 42,462 ATAC-seq peaks in introns and intergenic regions (total of 60,575 ATAC-seq peaks), 19.9% of which overlapped with STARR-seq enhancers. In line with the finding that PSCs are incapable of inducing an interferon type I response (*45*), only 85 of the accessible hiPSC enhancers (0.9%) overlapped with a previously defined set of 1517 interferon-related enhancers that are induced by plasmid transfection (*40*); these enhancers were excluded from further analyses. In addition, we omitted 29 putative enhancers that were enriched for the heterochromatin-associated mark trimethylation of histone H3 lysine-9 (H3K9me3) at their endogenous chromatin loci.

We note that a major fraction of STARR-seq-positive genomic regions are located in inaccessible chromatin (Fig. 1, A to C, and table S1), in agreement with previous studies (*33*, *43*, *46*, *47*). Although inaccessible genomic regions can have gene regulatory functions (*48*) and have been shown to function as enhancers in some cases (*26*), we conclude that the majority of STARR-seq+/ATAC-seq− enhancers most likely represent genomic sequences that have the ability to function as enhancers in episomal reporter assays but whose activity is repressed by the endogenous chromatin context in hiPSCs. We here refer to this enhancer class as chromatin-repressed enhancers.

**Fig. 1.**
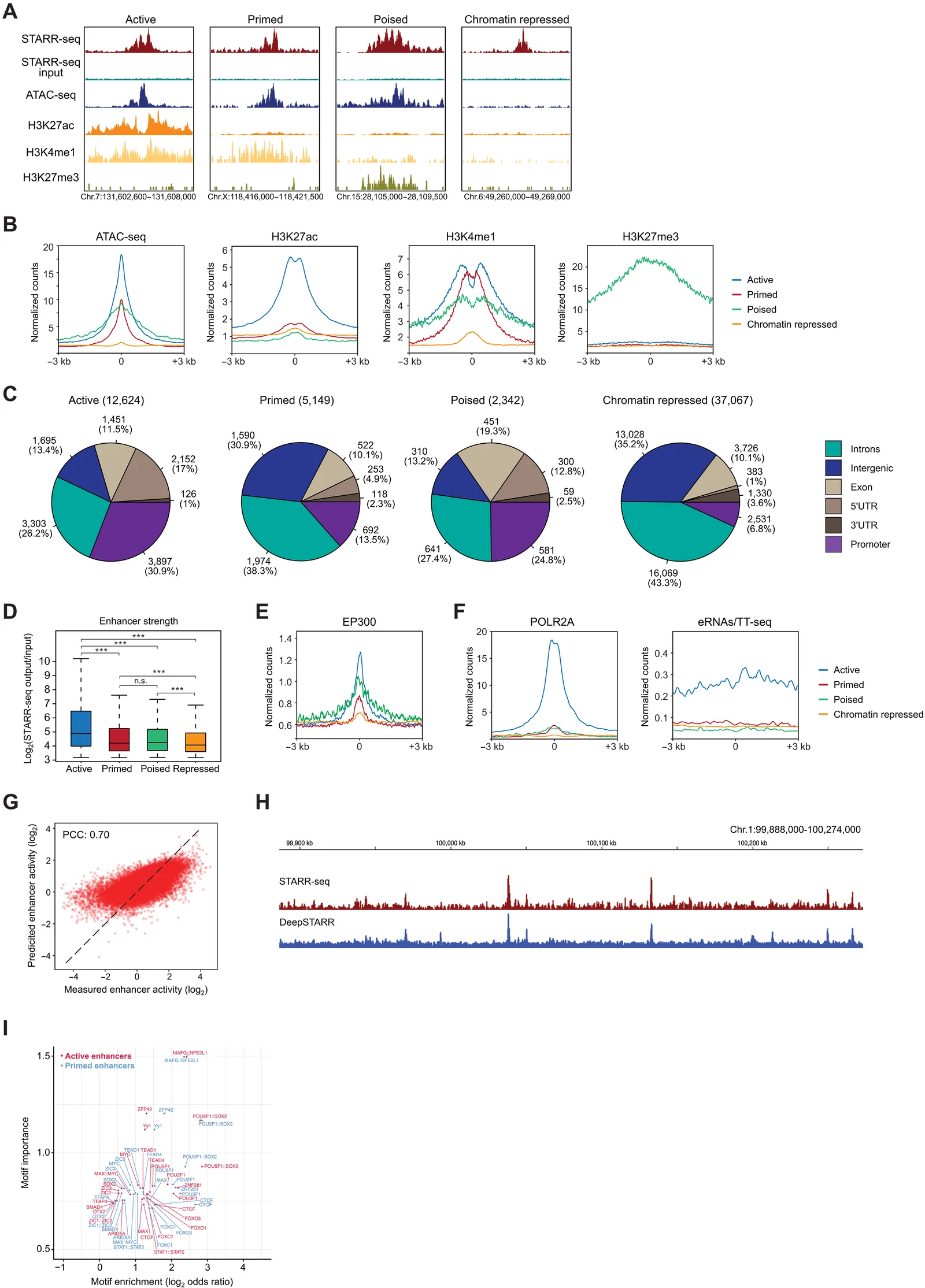
Identification and classification of putative enhancers in hiPSCs. (**A**) Coverage tracks of STARR-seq, ATAC-seq, and ChIP-seq (H3K27ac, H3K4me1, and H3K27me3) data in hiPSCs. Putative enhancers were mapped on the basis of STARR-seq signal enrichment (top) over background control (STARR-seq input). Active, primed, and poised enhancers are within accessible chromatin (ATAC-seq enrichment) and enriched for H3K4me1 and H3K27ac (active), H3K4me1 (primed), or H3K4me1 and H3K27me3 (poised). Chromatin-repressed enhancers are devoid of ATAC-seq signal and any of the analyzed histone marks. Chr, chromosome. (**B**) Profile plots of ATAC-seq and ChIP-seq (H3K27ac, H3K4me1, and H3K27me3) normalized counts at enhancers +/−3 kb of STARR-seq peak centers. (**C**) Numbers (and % of total) of enhancers within indicated genomic features. 3′UTR, 3′ untranslated region; 5′UTR, 5′ untranslated region. (**D**) Box plot showing enhancer strength, defined as STARR-seq signal over STARR-seq input control (background). Two-sided Mann-Whitney *U* test with Benjamini-Hochberg correction for multiple testing. ***P < 0.001; n.s., nonsignificant. (**E**) Profile plot of ChIP-seq EP300 normalized counts at enhancers +/−3 kb of STARR-seq peak centers. Color legend as shown in (F). (**F**) Profile plots of RNAPII ChIP-seq (POLR2A; left) and TT-seq (right) normalized counts at enhancers +/−3 kb of STARR-seq peak centers. (**G**) Pearson correlation (PCC) of DeepSTARR-predicted versus observed enhancer activity genome-wide. (**H**) Example coverage tracks of STARR-seq data (red) in comparison to DeepSTARR-predicted (blue) profiles. (**I**) Scatterplot showing motif enrichment (*x* axis) and DeepSTARR-predicted global importance (*y* axis) at active enhancers (red) and primed enhancers (blue). Note that only a subset of identified motifs is shown.

### Epigenetic modifications and transcription factor occupancy at hiPSC enhancers

On the basis of the enrichment of posttranslational histone modifications H3K4me1, H3K27ac, and H3K27me3, we classified STARR-seq+/ATAC-seq+ intergenic and intronic regions into three categories: 4998 active enhancers (enriched for H3K4me1 and H3K27ac), 3564 primed enhancers (enriched for H3K4me1), and 951 poised enhancers (enriched for H3K4me1 and H3K27me3; Fig. 1, A to C, and tables S2 to S4). To ensure that our results are not confined to a specific hiPSC line, we generated a second STARR-seq library from an independently derived hiPSC line (HPSI0214i-wibj_2; Wibj2 hereafter). While this second STARR-seq dataset was less complex, with a lower signal-to-noise ratio than the original STARR-seq dataset, the overall reproducibility was high, with 72.1% of Wibj2 STARR-seq peaks shared with Yoch6 (fig. S1, E and F). Furthermore, the percentages of active, primed, and poised enhancers were highly similar across the two STARR-seq datasets (fig. S1G), demonstrating that our STARR-seq enhancer categorization is reproducible in independently derived hiPSC lines.

As expected, active enhancers can be distinguished from the other enhancer classes by their higher STARR-seq activity (Fig. 1D). Furthermore, EP300 and RNA polymerase II (RNAPII) occupancy was higher at active enhancers compared to primed and poised enhancers, concomitant with enhanced levels of eRNA transcription (Fig. 1, E and F). Collectively, our results establish a genome-scale functional annotation of enhancers in hPSCs.

To identify enhancer sequence motifs, we used our STARR-seq data to train an enhancer syntax deep-learning model [DeepSTARR (*49*)]. The predicted and observed enhancer activity profiles were highly similar genome-wide [Pearson correlation coefficient (PCC) = 0.70; Fig. 1, G and H], closely matching the reported predictive performance of DeepSTARR for *Drosophila* enhancers (*49*). Next, we used the trained DeepSTARR model to predict the importance of enriched transcription factor motifs for active and primed enhancers. We found the enrichment of various motifs of transcription factors involved in the control of pluripotency in both enhancer classes including POU5F1(OCT4), POU5F1::SOX2, ZFP281, YY1, and ZFP42(REX1) (Fig. 1I and fig. S1, H to K). In addition, binding motifs for ZIC2 and ZIC3 were enriched, which are required to maintain primed pluripotency (*50*). Together, the sequence motifs in active and primed enhancers are consistent with an important function in the maintenance of the pluripotent state.

### Long-range chromatin contacts and target genes of human pluripotent enhancers

Enhancers can control genes over large genomic distances, in some cases, skipping over more proximally located genes to interact with their target genes (*4*). To capture regulatory chromatin contacts between enhancers and all human gene promoters, we carried out PCHi-C (*37*, *38*). To detect both mid- and long-range three-dimensional (3D) chromatin interactions between enhancers and promoters, we generated PCHi-C maps using two different restriction enzymes (Dpn II for higher-resolution and mid-range interactions and Hind III for long-range interactions; fig. S2A and tables S5 and S6). Using HiCUP (*51*), we confirmed that all PCHi-C libraries had a high percentage of valid reads (78.1 to 98%) and cis reads, representing interactions on the same chromosome (71.6 to 84.6%; fig. S2B), indicative of high-quality chromatin interaction libraries. The individual PCHi-C libraries were sequenced to a depth between 84.3 million and 170.6 million unique promoter-centered reads (fig. S2C), totaling over 1.95 billion promoter interaction reads. CHiCAGO (*52*) interaction calling uncovered 534,042 statistically significant promoter contacts (242,343 in Hind III PCHi-C libraries and 291,699 in Dpn II PCHi-C libraries), representing the most comprehensive pan-hiPSC 3D promoter interactome atlas to date.

We next determined which gene promoters contact pluripotent STARR-seq enhancers. This uncovered 3346, 1724, and 931 putative target genes of active, primed, and poised enhancers, respectively. Notably, we found that a major fraction of STARR-seq peaks do not engage in promoter contacts (Fig. 2A), indicating either that the promoter-enhancer contacts are too dynamic to be captured or that these putative enhancers may regulate their target genes without physical association in the 3D nuclear space, as has been described for individual cases of “contactless” enhancers (*53*, *54*). As expected, genes interacting with active enhancers showed the highest expression levels (Fig. 2B and fig. S2D). Assigning enhancers to their putative target genes on the basis of genomic distance (+/−100 kb) revealed the same trend (Fig. 2C). Notably, genes interacting with, or located in proximity to, primed enhancers are more highly expressed than genes interacting with poised enhancers (Fig. 2, B and C, and fig. S2D), which have been shown to interact with their silenced target genes in PSCs (*55*).

**Fig. 2.**
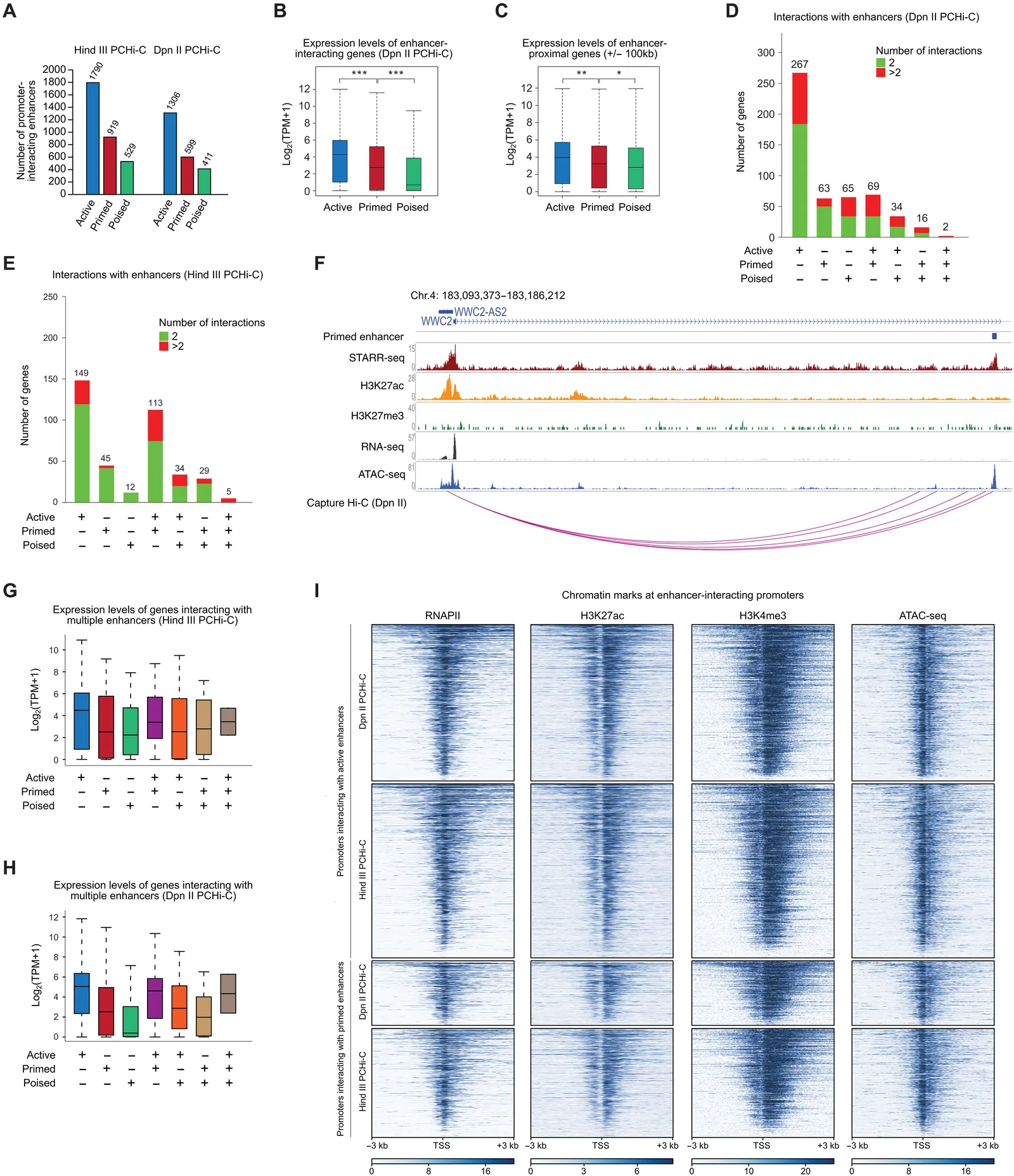
Long-range chromatin interactions of hiPSC enhancers. (**A**) Number of interactions of promoters with active, primed, or poised enhancers identified using Hind III PCHi-C (left) and Dpn II PCHi-C (right). (**B**) Expression levels [RNA-seq, log_2_(normalized counts + 1)] of enhancer-interacting genes. Interactions were mapped using Dpn II PCHi-C data. Two-sided Mann-Whitney *U* test with Benjamini-Hochberg correction for multiple testing. ***P < 0.001. TPM, transcripts per million. (**C**) Expression levels [RNA-seq, log_2_(normalized counts +1)] of enhancer-proximal genes (+/−100 kb). Two-sided Mann-Whitney *U* test with Benjamini-Hochberg correction for multiple testing. *P < 0.05; **P < 0.01. (**D**) Number of genes interacting with two or more active, primed, or poised enhancers or combinations of enhancers. Interactions were mapped using Dpn II PCHi-C data. (**E**) Number of genes interacting with two or more active, primed, or poised enhancers or combinations of enhancers. Interactions were mapped using Hind III PCHi-C data. (**F**) Coverage tracks of genome-wide data on chromosome 4 showing a representative example of a primed enhancer, which interacts with an expressed gene (*WWC2*). Capture Hi-C corresponds to Dpn II PCHi-C data. (**G**) Expression levels [RNA-seq, log_2_(normalized counts + 1)] of genes interacting with two or more active, primed, or poised enhancers or combinations of enhancers [categories as in (E)]. Interactions were mapped using Hind III PCHi-C data. (**H**) Expression levels [RNA-seq, log_2_(normalized counts + 1)] of genes interacting with two or more active, primed, or poised enhancers or combinations of enhancers [categories as in (D)]. Interactions were mapped using Dpn II PCHi-C data. (**I**) Heatmap showing H3K27ac and H3K4me3 enrichment (ChIP-seq) and ATAC-seq signal at promoters interacting with active or primed enhancers +/−3 kb of transcription start sites (TSSs) in primed hiPSCs. Interactions were mapped using Hind III PCHi-C or Dpn II PCHi-C data.

We found that most genes engage in long-range chromatin contacts with just one enhancer (fig. S2, E to H). To also investigate more complex enhancer-promoter 3D networks, in which genes receive regulatory inputs from more than one enhancer, we separated genes by both the number and activity status of their interacting enhancers. This revealed numerous instances of homotypic and heterotypic enhancer interactions (Fig. 2, D to F), possibly contributing to a coordinated control of fine-tuned target gene expression levels (Fig. 2, G and H).

Last, we determined chromatin accessibility (ATAC-seq), active chromatin marks (H3K4me3 and H3K27ac), and RNAPII occupancy at the promoter regions of enhancer-interacting genes. Despite clearly distinct gene expression levels (Fig. 2B and fig. S2D), we detected only minor differences in RNAPII occupancy and H3K27ac enrichment at promoters interacting with active enhancers compared to promoters interacting with primed enhancers, and the H3K4me3 and ATAC-seq profiles were nearly indistinguishable (Fig. 2I). Thus, chromatin accessibility, histone marks (Fig. 1B), and RNAPII occupancy (Fig. 1F) at enhancers appear to correlate more strongly with gene expression levels than the corresponding marks at the enhancer-interacting gene promoters.

### Chromatin mark dynamics at hiPSC enhancers during cell lineage specification

Primed enhancers are thought to exist in a neutral and inactive state, poised for activation upon acquisition of the active histone mark H3K27ac during cell lineage diversification. To test this prediction for the primed hiPSC STARR-seq enhancers (STARR-seq+ ATAC-seq+ H3K27ac−), we used previously published epigenome profiling data from cell types that represent key lineages in the human embryo: trophoblast-like cells (TBL), mesendoderm (MES), neural progenitor cells (NPCs), and mesenchymal stem cells (MSCs) (*56*). We found that 18.5% of candidate primed enhancers gained H3K27ac specifically in one of the four lineages (“lineage-restricted”; MES: 9.2%; TBL: 3.1%; NPC: 4%; MSC: 2.2%), whereas 20.2% of candidate primed enhancers gained H3K27ac in all four lineages (“multilineage”; Fig. 3A). However, most (61.3%) primed enhancers failed to acquire the active enhancer mark H3K27ac in any of the cell types interrogated (Fig. 3, A and B), comparable to poised and chromatin-repressed enhancers (Fig. 3B and fig. S3, A and B). In contrast, most active enhancers (STARR-seq+ ATAC-seq+ H3K27ac+) maintained H3K27ac in all lineages (53.7%), whereas 18.1% of active enhancers maintained H3K27ac selectively in one lineage (“lineage-restricted”; MES: 11%; TBL: 1.9%; NPC: 4.2%; MSC: 1%), and 28.2% of active enhancers harbor H3K27ac exclusively in hiPSCs (Fig. 3, B and C). We next tested whether primed enhancers carry active enhancer marks in naïve hPSCs, which represent an earlier stage of human development. Using published H3K27ac data (*57*), we established that most active hiPSC enhancers are marked with H3K27ac already in naïve hPSCs (Fig. 3, D and E). In contrast, most primed, poised, and chromatin-repressed enhancers are not enriched for H3K27ac in naïve hPSCs (Fig. 3, D and F to H). Thus, we conclude that most primed enhancers in hiPSCs are not occupied by H3K27ac in either developmental progenitor states or major cell lineages derived from hPSCs. This observation is consistent with enhancer priming for activation in cell types or states that we have not interrogated in this study, including at later developmental stages. Alternatively, primed enhancers may have yet-to-be-identified functions in hPSCs.

**Fig. 3.**
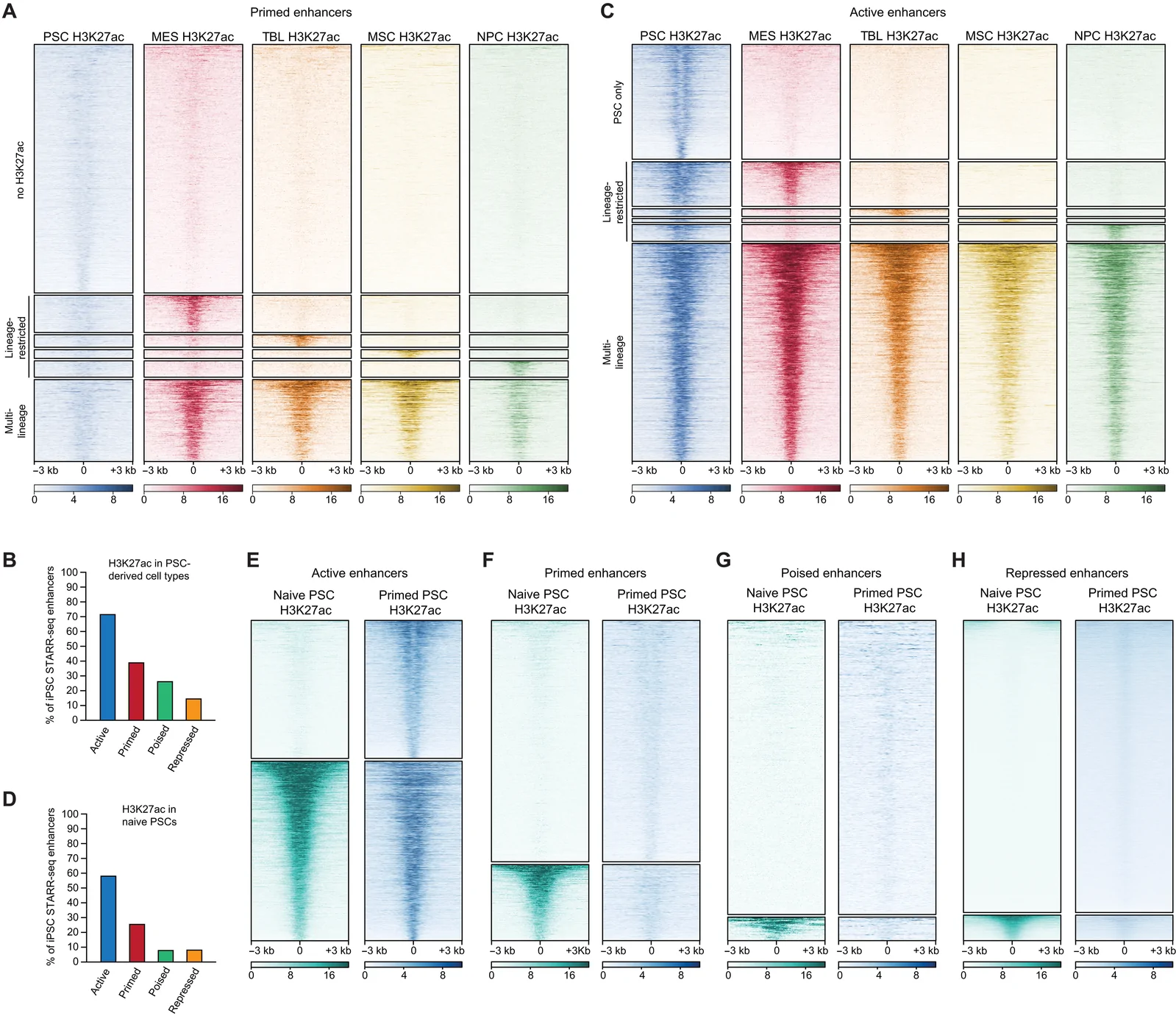
The majority of primed enhancers do not gain H3K27ac during differentiation. (**A**) Heatmap showing H3K27ac enrichment (ChIP-seq) at primed enhancers +/−3 kb of STARR-seq peak centers in primed hPSCs, MES, TBL, MSCs, and NPCs. Most enhancers are not enriched for H3K27ac in any of the analyzed cell types (no H3K27ac). Subsets are enriched for H3K27ac in one (lineage-restricted) or multiple differentiated cell types (multilineage). (**B**) Proportion (in %) of enhancers mapped in hiPSCs that maintain or gain H3K27ac in one or multiple differentiated cell types. (**C**) Heatmap showing H3K27ac enrichment (ChIP-seq) at active enhancers +/−3 kb of STARR-seq peak centers in PSCs, MES, TBL, MSCs, and NPCs. Subsets of enhancers are enriched for H3K27ac only in hiPSCs (PSC only) or in hiPSCs and one (lineage-restricted) or multiple cell types (multilineage). (**D**) Proportion (in %) of enhancers mapped in primed hiPSCs that are enriched for H3K27ac in naïve PSCs. (**E** to **H**) Heatmap showing H3K27ac enrichment (ChIP-seq) at active (E), primed (F), poised (G), and repressed (H) enhancers (mapped in primed hiPSCs) +/−3 kb of STARR-seq peak centers in naïve and primed PSCs.

### Sequence determinants of human pluripotent enhancers

Mutations in transcription factor binding sites within enhancers can lead to severe developmental malformations (*58*), and a growing number of diseases and disease susceptibilities have been linked to aberrant enhancer function caused by genetic variation *(**10**,* *11**)*. Deciphering how enhancer variants affect developmental gene expression programs and shape complex human traits is therefore a central goal of the “variant-to-function” challenge. However, our understanding of how naturally occurring genetic variation controls enhancer function in hPSCs is limited.

To identify functional enhancer variants, we pursued a two-pronged approach. First, we interrogated our STARR-seq data for heterozygous enhancer sequence variants that result in interallelic differences in enhancer activity. This approach revealed 16 and 15 functional enhancer variants in active and primed enhancers, respectively, with at least twofold differences in activity between major and minor enhancer alleles (Fig. 4, A and B). Next, as the absence of a haplotype-phased genome assembly for the hiPSC line used for our STARR-seq experiments prevented us from unambiguously assessing the effect of interallelic enhancer variants on the expression level of target genes, we focused on homozygous enhancer single-nucleotide polymorphisms (SNPs) that differ between individual hiPSC lines. As a proof of concept, we tested genetic variants for one active and two primed enhancers in a luciferase enhancer assay (Fig. 4C) using the *POU5F1* (*32*) enhancer as a positive control (fig. S4A). We used matching ATAC-seq (*59*) and RNA-seq (*60*) data from a hiPSC line in which these enhancers have opposing activities, i.e., enhancer 1 (enh1) is an active enhancer in the hiPSC line derived from donor 1 but a chromatin-repressed enhancer in the hiPSC line derived from donor 2 (Fig. 4D). Enhancer 2 (enh2) is a primed enhancer in the hiPSC line derived from donor 1 but a chromatin-repressed enhancer in the hiPSC line derived from donor 2 (Fig. 4E). Last, enhancer 3 (enh3) is a chromatin-repressed enhancer in the hiPSC line derived from donor 1 but a primed enhancer in the hiPSC line derived from donor 2 (Fig. 4F). For these enhancers, we detected pronounced interindividual differences in enhancer activity (Fig. 4C), which correlated positively with ATAC-seq peak strength and the expression levels for specific genes at the endogenous loci (*EEIG2* for enh1, *LAMC1* for enh2, and *ABHD4* for enh3; Fig. 4, D to F). Furthermore, we used AlphaGenome (*61*) to predict the effect of enhancer variants on histone modifications, which showed an increase in repressive or active marks consistent with their luciferase reporter activity (Fig. 4C and fig. S4B). These results indicate that genetic variants in active, primed, and chromatin-repressed enhancers have the potential to shape interindividual differences in gene expression programs between hiPSC lines derived from different donors.

**Fig. 4.**
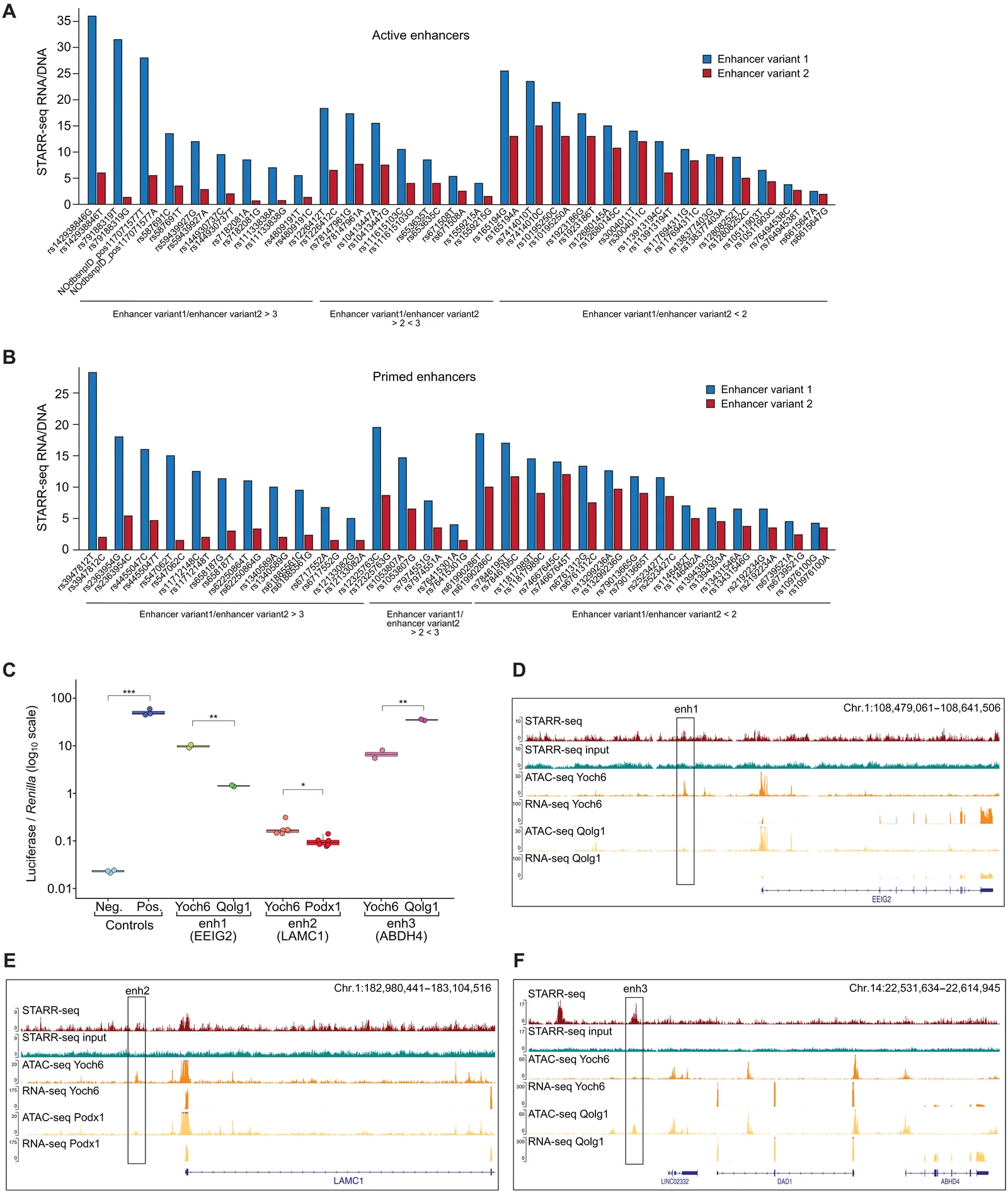
Sequence variation affects the activity of active and primed enhancers. (**A**) Enhancer activity measured by STARR-seq at active enhancers with interallelic sequence variation. (**B**) Enhancer activity measured by STARR-seq at primed enhancers with interallelic sequence variation. (**C**) Luciferase reporter assay for genetic variants in three hiPSC STARR-seq enhancers (enh1 to enh3). Enhancer sequences were derived from HipSci consortium whole-genome sequencing data (www.hipsci.org/#/) of three independent hiPSC lines (HPSI0215i-yoch_6, Yoch6; HPSI0215i-qolg_1, Qolg1; HPSI0215i-podx_1, Podx1). Negative control: empty vector; positive control: distal enhancer of *POU5F1*. Each data point corresponds to one biological independent replicate. *n* = 2 to 6; two-tailed unpaired Student’s *t* test: *P < 0.05; **P < 0.01; ***P < 0.001. (**D**) Coverage tracks of STARR-seq (Yoch6) as well as ATAC-seq and RNA-seq data from two independent hiPSC lines (Yoch6 and Qolg1) for enh1 [see (C)]. (**E**) Coverage tracks of STARR-seq (Yoch6) as well as ATAC-seq and RNA-seq data from two independent hiPSC lines (Yoch6 and Podx1) for enh2 [see (C)]. (**F**) Coverage tracks of STARR-seq (Yoch6) as well as ATAC-seq and RNA-seq data from two independent hiPSC lines (Yoch6 and Qolg1) for enh3 [see (C)].

### CRISPR enhancer perturbation reveals primed enhancer activity in hPSCs

Given that (i) most primed STARR-seq enhancers do not acquire active enhancer signatures in key PSC-derived lineages (Fig. 3, A and B), (ii) sequence variants affect primed enhancer strength in hPSCs (Fig. 4, B to F), and (iii) primed enhancers interact with genes that are expressed at moderate levels (Fig. 2B and fig. S2D), it is possible that primed enhancers control gene expression in hiPSCs, contradicting current models of primed enhancer function. To test this hypothesis directly, we used a doxycycline (DOX)–inducible CRISPR inactivation/activation system (*16*) to modulate enhancer function within the endogenous chromatin contexts. Targeting the KRAB transcriptional repressor domain to enhancers (CRISPRi), a widely used method to interrogate enhancer function (*62*–*64*), resulted in transcriptional down-regulation of the putative primed enhancer target genes *APLN*, *XPNPEP2*, *LAGE3*, and *SLC13A4* at levels comparable to the active enhancer target genes *EEIG2* and *SFRP2* (*23*), which we used as positive controls (Fig. 5A). Conversely, targeting the transcriptional activator VP64 [CRISPR activation (CRISPRa)] to primed enhancers resulted in transcriptional up-regulation of target genes *APLN* and *XPNPEP2* (Fig. 5B). In addition, we used CRISPR to delete three primed enhancers from their endogenous genomic loci (Fig. 5, C and D). In all three cases, primed enhancer deletion resulted in a pronounced down-regulation of the respective target gene expression (*APLN*, *PDLIM1*, and *SOX4*). These results demonstrate that interfering with primed enhancer activity results in changes in target gene expression in hPSCs, challenging the current model for primed enhancer function that predicts that this class of enhancers becomes active only at later developmental stages in more specialized cell types.

**Fig. 5.**
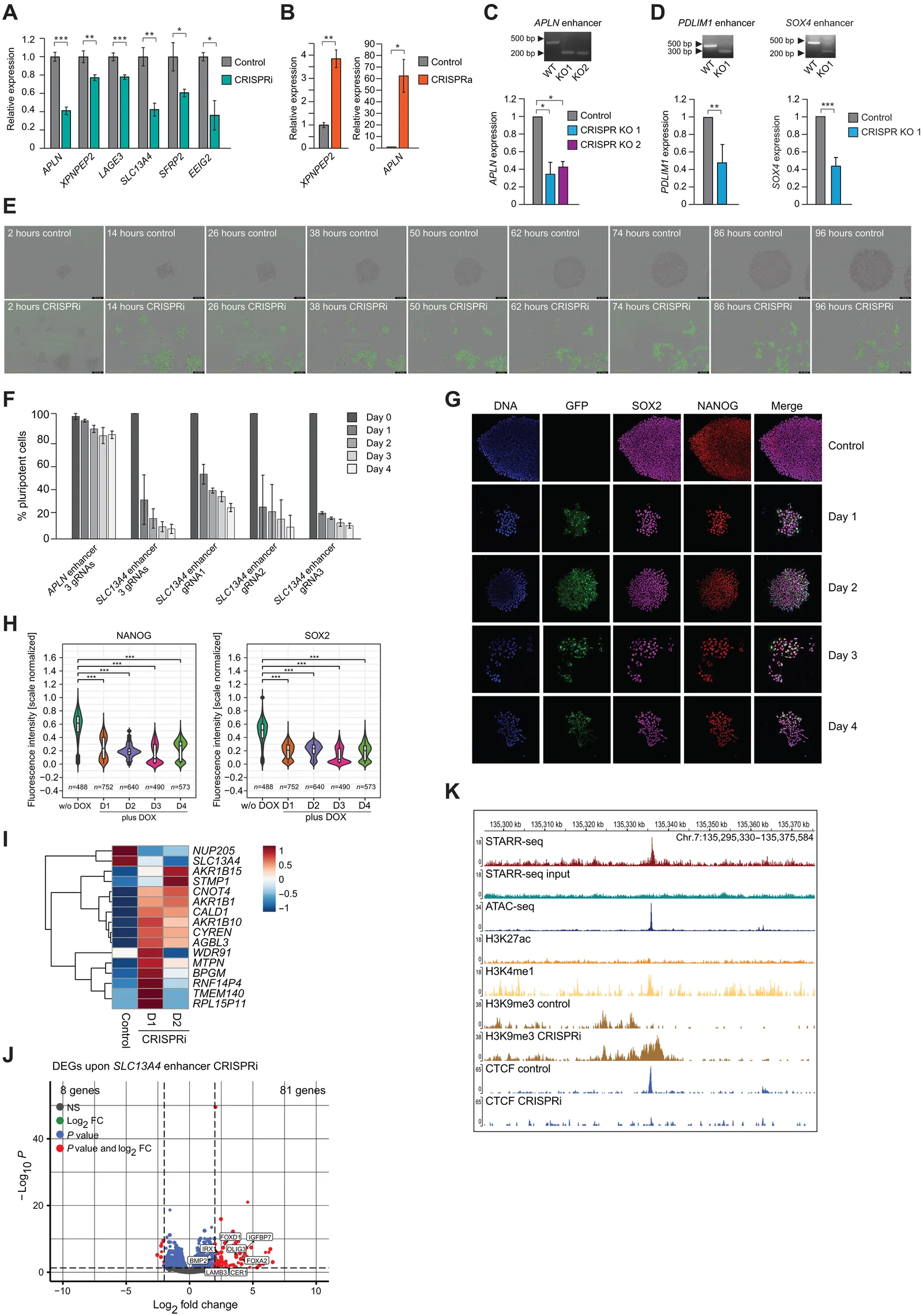
Repression or deletion of primed enhancers reduces target gene expression. (**A** and **B**) qRT-PCR for indicated genes before (control) or after DOX-induced expression of dCas9-KRAB (CRISPRi) (A) or dCas9-SunTag-VP64 (CRISPRa) (B) and gRNAs targeting primed enhancers or active enhancers (*SFRP2*, *EEIG2*) in hiPSCs. 2^−ΔΔCt^ ± SD; normalization using *GAPDH*. Two-tailed paired Student’s *t* test: *P < 0.05; **P < 0.01; ***P < 0.001. (**C** and **D**) Top: Gel electrophoresis analysis of PCR amplification of genomic DNA using primers flanking the *APLN* (C), *PDLIM1* [(D), left], or *SOX4* [(D), right] enhancer in wild-type (WT) and knockout (KO) clones. Bottom: qRT-PCR for target gene expression in wild-type and enhancer knockout clones. 2^−ΔΔCt^ ± SD; normalization using *GAPDH*. Two-tailed paired Student’s *t* test: *P < 0.05; **P < 0.01; ***P < 0.001. (**E**) Live-cell imaging of hiPSCs without (control) or with DOX/TMP (CRISPRi) to induce the expression of dCas9-KRAB (GFP-positive cells) with three gRNAs targeting the primed *SLC13A4* enhancer. (**F**) Proportion of remaining cells (in %) during live-cell imaging as described in (E). (**G**) Immunofluorescence images for SOX2 and NANOG of hiPSCs before (control) or after addition of DOX/TMP (days 1 to 4) for dCas9-KRAB expression (GFP-positive cells) with three gRNAs. DNA was counterstained. (**H**) Quantification of fluorescence intensities of images shown in (G). Two-sided Mann-Whitney *U* test with Benjamini-Hochberg correction for multiple testing. ***P < 0.001. (**I**) The heatmap shows relative expression levels of genes +/−1 Mb of the *SLC13A4* locus on the basis of RNA-seq data for hiPSCs after the addition of DOX/TMP for 1 day [day 1 (D1)] or 2 days [day 2 (D2)] to induce dCas9-KRAB with constitutive expression of gRNAs targeting the enhancer of *SLC13A4*. (**J**) The volcano plot shows differentially expressed genes of RNA-seq data at D1, as described in (I). NS, not significant; FC, fold change. (**K**) Coverage tracks showing the *SLC13A4* enhancer region in hiPSCs and H3K9me3 and CTCF CUT&Tag tracks showing the same *SLC13A4* enhancer genomic region in control and enhancer CRISPRi conditions.

### A CTCF-bound primed enhancer is required for human pluripotency

Notably, CRISPRi targeting of one primed enhancer resulted not only in *SLC13A4* transcriptional down-regulation (Fig. 5A) but also in pronounced changes of hiPSC colony morphology and substantial cell death, as revealed by live-cell imaging over 4 days (Fig. 5E and movie S1). In contrast, in non–CRISPRi-induced control hiPSCs, colony formation, hiPSC morphology, and cell survival were unaffected (Fig. 5E and movie S2). In addition, CRISPRi targeting of the primed enhancer controlling *APLN* had only minor effects on cell viability (Fig. 5F) and morphology (fig. S5A and movies S3 and S4). The changes in hiPSC colony morphology could be recapitulated with all three *SLC13A4* enhancer–targeting guide RNAs (gRNAs) used individually in CRISPRi, albeit to differing degrees (Fig. 5F and fig. S5, B to D). Furthermore, we recapitulated the changes in hiPSC morphology upon *SLC13A4* enhancer perturbation in an independent transfection using a different hiPSC line (movies S5 and S6), demonstrating that neither donor genetic background nor the genomic integration sites of the dCas9-KRAB and gRNA plasmids can account for the observed phenotype. As an additional control, we detected no correlation between dCas9 induction levels and the severity of the induced phenotype, arguing against unspecific effects on cell fitness caused by dCas9-KRAB overexpression (fig. S5E).

Although the remaining hiPSC colonies expressed the pluripotency markers SOX2 and NANOG after *SLC13A4* enhancer perturbation (Fig. 5G), quantification of the immunofluorescence images revealed reduced protein levels for both pluripotency factors (Fig. 5H). To determine whether the *SLC13A4* enhancer controls additional target genes, we carried out RNA-seq in hiPSCs on days 1 and 2 after dCas9-KRAB induction. Within a 2-Mb genomic window around the enhancer, only *NUP205*, a component of the nuclear pore complex, and *SLC13A4* showed consistent down-regulation upon CRISPRi, whereas several other genes in the locus displayed moderate expression changes including mild transcriptional up-regulation and oscillations between up- and down-regulation (Fig. 5I). At the genome-scale level, we detected up-regulation of several differentiation markers upon enhancer CRISPRi, including *FOXD1*, *FOXA2*, *IGFBP7*, and *OLIG3* (Fig. 5J). Gene ontology analyses of differentially expressed genes revealed an enrichment for tissues of mesodermal origin, consistent with aberrant expression of *FOXD1* and *FOXA2* (fig. S5F). Together, these findings are indicative of a partial loss of PSC features as a consequence of impaired primed *SLC13A4* enhancer function.

To control for possible CRISPRi off-target effects, we next carried out CUT&Tag against H3K9me3, the histone modification induced by dCas9-KRAB. We found that H3K9me3 spreading is limited to a region of ∼6 kb around the genomic site targeted by the gRNAs (Fig. 5K) without detectable changes to H3K9me3 levels on any of the gene promoters or gene bodies in the *SLC13A4* enhancer locus.

Consensus CCCTC-binding factor (CTCF) binding sites are among the most strongly enriched sequence motifs of primed hiPSC enhancers (Fig. 1I and fig. S1, H to K). Notably, CUT&Tag revealed pronounced CTCF binding to the *SLC13A4* enhancer (Fig. 5K). Upon CRISPRi, CTCF binding to the enhancer is markedly reduced (Fig. 5K), indicating that CTCF occupancy may be required for appropriate enhancer function. Last, we attempted to delete the primed *SLC13A4* enhancer from its endogenous locus in the hPSC genome. Unlike for the other three primed enhancer deletions described above (Fig. 5, C and D), we were unable to derive a stable, homozygous iPSC line harboring a deletion for the *SLC13A4* enhancer. However, initial CRISPR targeting to the enhancer was successful as determined by standard and digital droplet polymerase chain reaction (ddPCR) (fig. S5, G and H), which suggests that clones with a homozygous *SLC13A4* enhancer deletion may be selected against because of their impaired fitness and/or loss of pluripotency. While this prevented us from further characterizing the *SLC13A4* enhancer phenotype, the result is consistent with the enhancer having an indispensable function in hPSCs. Collectively, our data identify a hitherto uncharacterized gene control element as an essential component of the gene regulatory circuitry that maintains the pluripotent state of hiPSCs.

### Putative noncanonical enhancers are widespread in the human genome

Our results demonstrate that some primed enhancers can function as bona fide enhancers in hPSCs despite lacking the active enhancer mark H3K27ac. Similar enhancer activities have been reported for noncanonical enhancers in mouse ESCs and human K562 cells (*30*), which are marked by acetylated lysine residues in the globular domain of histone H3, including lysine-122. We found that H3K122ac is not enriched at primed STARR-seq enhancers compared to active STARR-seq enhancers in hPSCs (Fig. 6A), suggesting that the enhancers we uncovered here are functionally distinct from mouse stem cell noncanonical enhancers. Acetylation at specific histone H2B, H3, and H4 lysine residues has also been linked to enhancer function (*30*, *65*, *66*). However, we found that acetylation at histone H2B lysine-5 or lysine-12 was not enriched at primed enhancers compared to active enhancers (Fig. 6A). Acetylation of specific histone H4 lysine residues showed a similar trend (Fig. 6B), except for a slight enrichment of H4K16ac at primed enhancers over active enhancers in hPSCs (Fig. 6, B and C). Thus, it remains to be determined whether the noncanonical enhancers uncovered in this study can be identified by a characteristic chromatin mark signature.

**Fig. 6.**
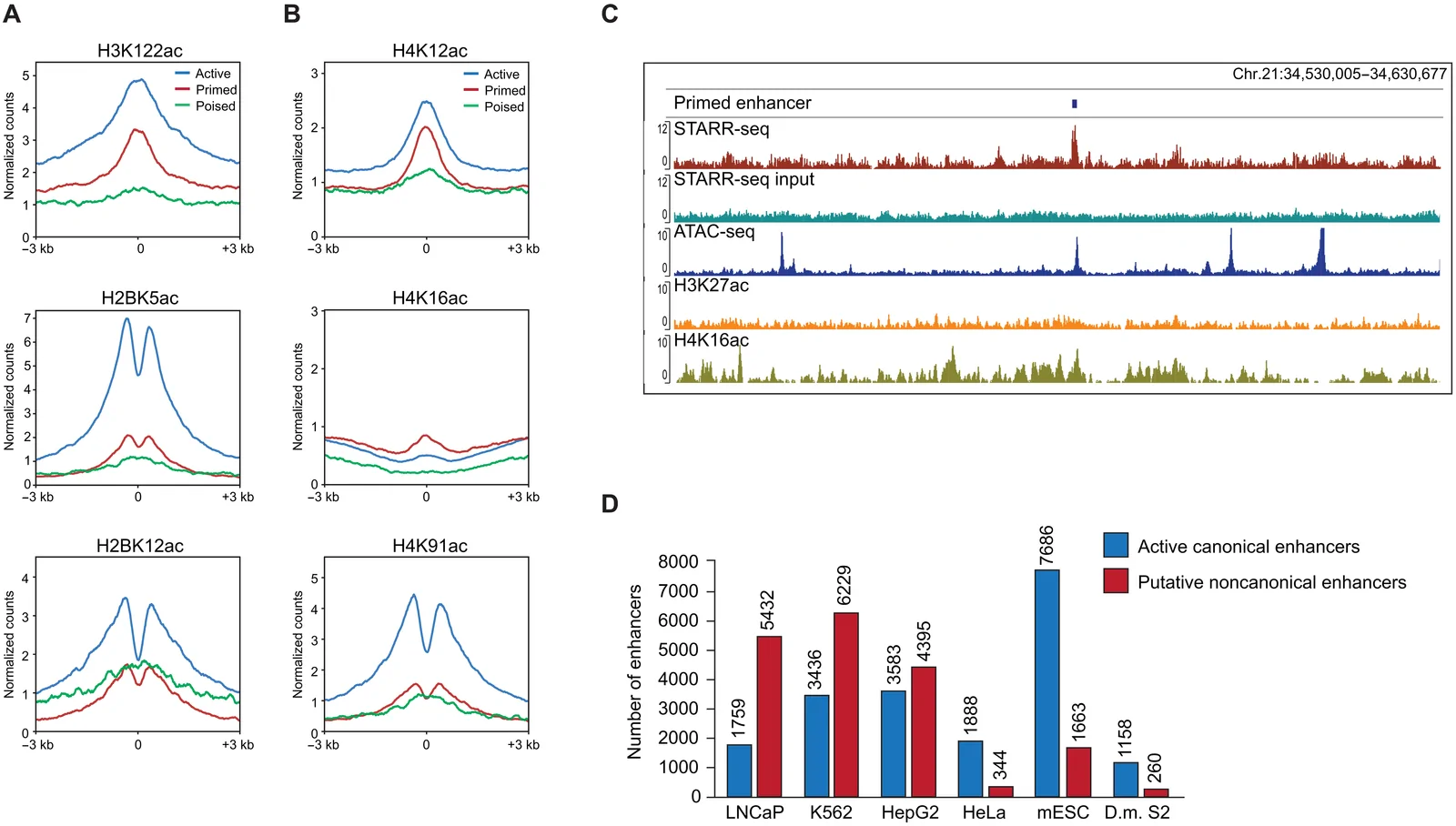
Putative noncanonical enhancers in *Drosophila*, mouse, and human cells. (**A**) Profile plots of H3K122ac (top), H2BK5ac (middle), and H2BK12ac (bottom) at active, primed, and poised enhancers +/−3 kb of STARR-seq peak centers. (**B**) Profile plots of H4K12ac (top), H4K16ac (middle), and H4K91ac (bottom) at active, primed, and poised enhancers +/−3 kb of STARR-seq peak centers. (**C**) Coverage tracks of STARR-seq, ATAC-seq, ChIP-seq (H3K27ac), and H4K16ac CUT&Tag, demonstrating an example of H4K16ac enrichment over the indicated primed enhancer. (**D**) Number of active canonical and putative noncanonical enhancers in published STARR-seq datasets from LNCaP cells, K562 cells, HepG2 cells, HeLa cells, mouse ESCs, and *Drosophila melanogaster* (D.m.) S2 cells.

We next asked whether the putative novel enhancer class our data have revealed is only found in hPSCs. Reanalyzing publicly available genome-wide STARR-seq datasets (*33*, *43*, *46*, *47*), we found that putative noncanonical enhancers (H3K27ac−, ATAC-seq+, and STARR-seq+) can also readily be identified in human cancer cell lines (LNCaP, K562, HepG2, and HeLa) and, to a lesser extent, in mouse ESCs and *Drosophila* S2 cells (Fig. 6D). This observation cannot be explained by the number of H3K27ac peaks called in the respective datasets (fig. S6A). Furthermore, the genomic locations of the putative noncanonical enhancers are consistent with their function as transcriptional enhancers (fig. S6B). Although targeted enhancer perturbation experiments are currently lacking to directly test this hypothesis in cell types other than hPSCs, these findings suggest that noncanonical enhancers devoid of the active enhancer mark H3K27ac may be widespread in the human genome across a broad range of cell types.

## DISCUSSION

Here, we present a genome-scale functional annotation of enhancers in hPSCs using a combination of massively parallel reporter gene assays and functional genomics profiling approaches, supported by live-cell imaging and CRISPR epigenome editing. Among our key findings, we show that enhancers lacking the active enhancer mark H3K27ac can function as bona fide enhancers in hPSCs. This class of enhancers (ATAC-seq+, H3K4me1+, and H3K27ac−) has previously been described as primed, neutral, or inactive enhancers (*19*–*21*, *67*). The current model of primed/neutral/inactive enhancer function predicts that this class of enhancers is transcriptionally inert and only controls target gene expression upon acquisition of the active mark H3K27ac in more specialized cell types. Our findings directly challenge this model, as we demonstrate that primed enhancer perturbation or deletion results in reduced transcriptional output from target genes in iPSCs. Underscoring the functional importance of this enhancer class for human pluripotency, we find that CRISPRi targeting of one primed enhancer results in impaired cell viability and loss of pluripotency features, including changes in stem cell colony morphology, reduced pluripotency marker expression levels, and aberrant expression of differentiation markers. At the gene level, CRISPR inactivation of this enhancer leads to overall subtle expression changes of several genes in the locus, with only *NUP205* and *SLC13A4* displaying reduced expression. *NUP205* encodes an inner nucleoporin subunit of the nuclear pore complex, and mutations in *NUP205* have been shown to cause steroid-resistant nephrotic syndrome (*68*) and abnormal cardiac left-right patterning (*69*). *SLC13A4* encodes a transmembrane protein that controls the transport of sulfate, which is essential for various metabolic pathways. Conditional knockout of *SLC13A4* in the placenta causes embryonic lethality, suggesting that the control of sulfate transport to the fetus is critical for development (*70*, *71*). Furthermore, *Slc13a4* haploinsufficient mice show impaired postnatal brain development (*72*). In hiPSCs, *SLC13A4* is expressed at relatively low levels, which, at first glance, makes it an unlikely candidate for a gene whose misexpression causes a pronounced effect on hiPSC viability and morphology. However, we note that, similarly, lowly expressed genes such as *FANCB* and *FAM71F1* are essential for human pluripotency (*73*). Thus, it is conceivable that subtle changes in the expression levels of *NUP205* and *SLC13A4* are causal for the impaired iPSC viability we report here. An alternative explanation is that the phenotype we observe is the combined result of a transcriptional imbalance across multiple genes in the locus upon enhancer perturbation. The precise mechanisms of action of this enhancer remain to be elucidated in future studies.

In this study, we report the discovery of a class of noncanonical enhancers in hPSCs, which lack the two most commonly used markers to identify active enhancers: H3K27ac and eRNAs. This raises questions as to how universally applicable these marks are for active enhancer discovery. Acetylation of H3K27 is mediated by the transcriptional coactivator and histone acetyltransferase CBP/EP300, neutralizing the positive charge of histone H3. This is thought to lead to chromatin decondensation to increase accessibility for binding of transcription factors to activate enhancers (*74*). Notably, however, the chromatin accessibility landscape is largely unaffected upon genome-wide hypoacetylation by p300/CBP inhibition (*75*). Moreover, H3K27ac enrichment at enhancers does not necessarily correlate with their activity, as most H3K27ac-enriched enhancers tested in human ESCs (hESCs) or in mouse embryos do not drive reporter gene expression (*18*, *23*, *26*, *27*). A mutation of the H3K27 residue (H3K27R) in flies or mouse ESCs does not interfere with enhancer activity and gene induction during differentiation, suggesting that acetylation of H3K27 is dispensable for transcription (*28*, *29*, *76*). The concomitant lack of H3K27 methylation in H3K27R mutant cells results in transcriptional derepression of PRC2 target genes, indicating that H3K27ac might serve as a “placeholder” to prevent H3K27 methylation (*28*, *76*). These studies suggest that H3K27ac may not be essential for enhancer activation, which could also be due to a degree of redundancy as other histone residues can also be acetylated. In mouse ESCs, active noncanonical enhancers are enriched for H3K122ac and H3K64ac (*30*). Similarly, histone H2B acetylation was found at active enhancers in mouse ESCs and human cells (*65*). The active noncanonical enhancers identified in this study lack H3K27ac but show modest enrichment for several other histone acetylation marks. These marks are generally present at lower levels than in canonical active enhancers, with only H4K16ac showing a modest enrichment at noncanonical enhancers compared with canonical enhancers. Future studies are therefore needed to determine whether the class of noncanonical enhancers described here harbors an as-yet undefined specific signature of posttranslational histone modifications.

We found that a substantial fraction of STARR-seq enhancers in hiPSCs (25% for active enhancers and 21.3% for noncanonical enhancers) overlap with CTCF binding. While recent research has focused on the role of CTCF as a key architectural and insulator protein in 3D genome organization (*77*–*81*), earlier studies have attributed pleiotropic functions to CTCF, including those of a transcriptional repressor, activator, and enhancer-binding protein (*82*). For example, CTCF binds to developmental enhancers that promote adipocyte differentiation (*83*). Furthermore, a positive correlation between CTCF binding and enhancer activity has been demonstrated (*84*). Notably, a recent study reports that 17% of enhancers across cell types correspond to CTCF binding sites with no or very low H3K27ac enrichment (*85*). Together with our findings, this points to a hitherto underappreciated role for CTCF-bound enhancers in genome regulation and developmental gene control. However, we also note that most primed enhancers (including the *APLN*, *PDLIM1*, and *SOX4* enhancers that were deleted in this study; Fig. 5, C and D) are not occupied by CTCF. Therefore, CTCF binding is not a defining feature of noncanonical enhancers.

Active canonical enhancers are bound by EP300/CBP and RNAPII and are bidirectionally transcribed into noncoding eRNAs. In contrast, our data suggest that the noncanonical enhancers are bound by EP300/CBP but lack RNAPII occupancy and eRNA expression. This is consistent with the absence of H3K27ac at noncanonical enhancers, given that the histone acetyltransferase activity of CBP is enhanced by the interaction with eRNAs. The level of eRNA expression at active canonical enhancers is highly variable (*86*). Functionally, they can promote target gene expression and chromatin looping and enhance transcription factor occupancy (*22*). Thus, the lack of eRNA transcription at noncanonical enhancers may be related to the lower levels of target gene expression as compared to canonical active enhancer target genes.

This study has limitations that should be acknowledged. First, STARR-seq does not measure the activity of enhancers in their endogenous chromatin environments, and a previous study has reported pronounced differences in enhancer activities measured through episomal versus chromatin-integration assays (*87*). Here, we mitigate this limitation by integrating enhancer activity measured by STARR-seq with a range of complementary functional genomics assays that directly assess chromatin accessibility, posttranslational histone modifications, and transcriptional activity at the endogenous enhancer loci. Nonetheless, the number of enhancers that we have interrogated directly by CRISPR perturbation is limited, and future studies will address on a more comprehensive scale how widespread genuine enhancer activity is among hiPSC primed enhancers. Second, we have determined enhancer activity across the human pluripotent genome using only one promoter in our STARR-seq approach, namely the origin of replication (ORI) core promoter. Even though ORI has been thoroughly tested and is established as a highly inducible promoter that responds to human enhancers across a range of human cell types (*40*), *Drosophila* enhancers have been shown to exhibit marked specificity to different promoters (*88*), and we cannot, with certainty, exclude the possibility that some human pluripotent enhancers may not be functionally compatible with the ORI promoter.

Notably, we found a large number of intronic and intergenic H3K27ac-marked regions in pluripotent open chromatin (72.1%) that display no or very little STARR-seq enhancer activity, in line with a previous study (*23*). The corresponding enhancer class (STARR−, ATAC+, and H3K27ac+) in GP5d and HepG2 cells has recently been termed “chromatin-dependent,” and it has been suggested that these enhancers cannot be effectively detected using STARR-seq (*89*). While this remains a plausible explanation for at least some enhancers, biochemical assays such as H3K27ac chromatin immunoprecipitation followed by sequencing (ChIP-seq) (or H3K27ac CUT&Tag) profile a chromatin-associated mark that is correlated with enhancer activity, rather than directly demonstrating enhancer function. Notably, CRISPR deletion of H3K27ac+ STARR+ candidate enhancers resulted in reduced transcriptional output of target genes, whereas deletion of H3K27ac+ STARR− putative enhancers did not (*23*). Thus, enhancer function cannot be inferred from chromatin signatures alone, and large-scale enhancer perturbation approaches will be required to determine how many H3K27ac-marked putative enhancers have genuine enhancer activity and in which cellular contexts.

In summary, our data identify a novel class of noncanonical enhancers with essential functions in hPSCs. Our findings, along with those of others (*23*, *24*, *26*–*28*, *30*–*32*, *90*), indicate that the classification of enhancers into only three functional categories (active, primed, and poised) based on their posttranslational histone modification signatures does not accurately reflect the complexity of enhancers and their associated gene regulatory landscapes in mammalian cells. Future studies will aim to refine how this “enhancer histone code” translates signaling inputs into regulatory information relayed to target genes and how cell type and developmental context dependencies shape the activities of the diverse enhancer functional subcategories on the transcriptional outputs of their target genes.

## MATERIALS AND METHODS

### Cell culture

hiPSC lines in this study were generated using CytoTune-iPS from Life Technologies and purchased from the Human Induced Pluripotent Stem Cells Initiative (www.hipsci.org). HPSI0215i-yoch_6 and HPSI0214i-wibj_2 were used for STARR-seq; HPSI0215i-yoch_6 was used to generate TT-seq, CUT&Tag, luciferase assays, ATAC-seq, and RNA-seq; and HPSI0215i-yoch_6 and HPSI0114i-kolf_2 were used for CRISPR. All cell lines were cultured in TeSR-E8 media with supplement (Stemcell Technologies, 05990) on vitronectin (Thermo Fisher Scientific, A14700)–coated surfaces at 37°C under 5% CO_2_ and normal O_2_ levels. Media with supplement were refreshed every 24 hours. Rho-associated protein kinase (ROCK) inhibitor (10 μM; Cell Guidance Systems, Y-27632) was added to the culture for the first day after revival. Cells were passaged at ratios ranging from 1/6 to 1/8 using 0.5 mM EDTA when they were ∼70% confluent. For electroporation transfections in STARR-seq screening and luciferase assays, cells were cultured in TeSR-E8 media with supplement (Stemcell Technologies, 05990) and 10 μM ROCK inhibitor for 24 hours before electroporation and 6 hours (STARR-seq candidates) or 24 hours (STARR-seq controls and luciferase assay) after electroporation and recovery. After the first 24 hours, cells in STARR-seq controls were cultured for an additional 24 hours without ROCK inhibitor. Before experiments, cells were harvested and detached using Accutase solution (Sigma-Aldrich, A6964). Cell number and viability were analyzed using Countess Cell Counting Chamber Slides (Invitrogen, C10228) on the Countess II Automated Cell Counter (Invitrogen). Once revived, only the cells with no less than three passages and no more than 10 passages were harvested for experiments.

### ATAC-seq

ATAC-seq libraries were generated as described (*44*), with minor modifications. Fifty thousand cells were harvested with Accutase and centrifuged at 500*g* for 5 min at 4°C in a swing arm rotor centrifuge. The cells were lysed in 50 μl of cold ATAC Resuspension Buffer [10 mM tris-HCl (pH 7.5), 10 mM NaCl, and 3 mM MgCl_2_] containing 0.1% IGEPAL-630, 0.1% Tween 20, and 0.01% digitonin and incubated on ice for 3 min. The lysate was topped up with 1 ml of cold ATAC Resuspension Buffer containing 0.1% Tween 20 and underwent centrifugation at 500*g* for 10 min at 4°C in a swing arm rotor centrifuge. The pellet was resuspended in 50 μl of transposition mixture [1× Illumina Tagment DNA Buffer and 100 nM Illumina TDE1 Tagment DNA Enzyme transposase (Illumina; 20034197), 0.33× phosphate-buffered saline (PBS), 0.1% Tween 20, and 0.01% digitonin] and incubated at 37°C for 30 min with 1000-rpm mixing. After undergoing purification with the Zymo DNA Clean & Concentrator-5 kit (Zymo Research; D4003), samples were eluted in 21 μl of elution buffer and combined with 2.5 μl of 25 μM i5 primer, 2.5 μl of 25 μM i7 primer (table S7), and 25 μl of 2× NEBNext High-Fidelity 2X PCR Master Mix (NEB; M0541S). A polymerase chain reaction (PCR) was performed with the following conditions: 72°C for 5 min, 98°C for 30 s, and eight cycles of 98°C for 10 s, 63°C for 30 s, and 72°C for 1 min. The samples underwent a second Zymo DNA Clean & Concentrator-5 purification and were eluted in 30 μl of elution buffer before a 1.2× AMPure XP bead (Beckman Coulter; A63881) cleanup. Following QC (quality control) on a Bioanalyzer, libraries were multiplexed and sequenced [paired-end 50 base pairs (bp)] using a HiSeq 2000 instrument (Illumina).

### CUT&Tag

CUT&Tag was performed as described previously (*36*) with minor modifications using the EpiCypher protocol. hiPSCs were washed once with Dulbecco’s PBS (DPBS; Life Technologies; 14190144) and incubated with Accutase (Stemcell Technologies; 07922) for 5 min at 37°C. The cells were then centrifuged for 3 min at 300*g* at room temperature (RT), resuspended in 1× DPBS, and counted. A total of 2 × 10^5^ cells per CUT&Tag reaction were centrifuged for 3 min at 300*g* at RT and then resuspended in 100 μl per sample of cold NE Buffer [20 mM Hepes-KOH (pH 7.9), 10 mM KCl, 0.1% Triton X-100, 20% glycerol, 0.5 mM spermidine (Sigma-Aldrich, 05292), and 1× Roche cOmplete, Mini, EDTA-free Protease Inhibitor] and incubated for 10 min on ice. The nuclei were centrifuged for 3 min at 600*g* at RT and resuspended in cold NE Buffer to achieve a final concentration of 1 × 10^6^ nuclei/ml. A total of 1 × 10^5^ nuclei per reaction were subjected to CUT&Tag.

The total volume of concanavalin A beads (EpiCypher, 21-1401) of 11 μl per sample was transferred to a 1.5-ml tube for batch processing. Concanavalin A beads were washed twice on a magnetic stand with 100 μl per sample of Bead Activation Buffer [20 mM Hepes (pH 7.9), 10 mM KCl, 1 mM CaCl_2_, and 1 mM MnCl_2_] and resuspended in a final volume of 11 μl per sample. Ten microliters of beads was aliquoted into separate tubes for each sample and incubated with 100 μl of nuclei for 10 min at RT. The tubes were placed on a magnet separator (DynaMag-PCR; Thermo Fisher Scientific), and the supernatant was discarded. Beads were then resuspended in 50 μl of cold Antibody150 Buffer [20 mM Hepes (pH 7.5); 150 mM NaCl; 0.5 mM spermidine; 1× Roche cOmplete, Mini, EDTA-free Protease Inhibitor; 0.01% digitonin (Sigma-Aldrich, D141); and 2 mM EDTA] containing a primary antibody in a 1:50 dilution (anti-H3K9me3, Active Motif 39062; anti-CTCF, Active Motif 61932; anti-H3K122ac, Abcam ab33309; anti-H4K12ac, Diagenode C15410331-10). Samples were incubated overnight at 4°C on an orbital shaker.

The following day, the tubes were placed on a magnet separator, and the supernatant was discarded. The beads were incubated in Digitonin150 Buffer [20 mM Hepes (pH 7.5); 150 mM NaCl; 0.5 mM spermidine; 1× Roche cOmplete, Mini, EDTA-free Protease Inhibitor; and 0.01% digitonin] containing 0.5 μg of secondary antibody (anti-rabbit Epicypher; 13-0047) for 1 hour at RT. The beads were then washed twice with Digitonin150 Buffer and then resuspended in 50 μl of Digitonin300 Buffer [20 mM Hepes (pH 7.5); 300 mM NaCl; 0.5 mM spermidine; 1× Roche cOmplete, Mini, EDTA-free Protease Inhibitor; and 0.01% digitonin], and 1.25 μl of CUTANA pAG-TN5 (EpiCypher, 15-1017) was added. After incubation at RT for 1 hour, the tubes were placed on a magnet and washed twice with Digitonin300 Buffer.

The beads were then resuspended in 50 μl of chilled Tagmentation Buffer (Digitonin300 Buffer, 10 mM MgCl_2_) and incubated for 1 hour at 37°C in a thermocycler. The tube was placed on a magnetic separator, and the supernatant was discarded. The beads were washed once with 50 μl of RT TAPS Buffer [10 mM TAPS (pH 8.5) and 0.2 mM EDTA]. The beads were resuspended in 5 μl of RT SDS Release Buffer [10 mM TAPS (pH 8.5) and 0.1% SDS] and vortexed at maximum speed for 10 s, followed by a brief centrifugation. The beads were then incubated for 1 hour at 58°C in a thermocycler. Following incubation, 15 μl of RT SDS Quench Buffer (0.67% Triton-X 100 in molecular grade H_2_O) was added to the beads and vortexed at maximum speed for 20 s, followed by a brief centrifugation.

For library amplification, 1 μl of each barcoded i5 and i7 primer (10 μM stock) (table S7) was used, and PCR was performed using CUTANA High Fidelity PCR mix (EpiCypher, 15-1018). The following PCR program was used for all libraries: 58°C for 5 min, 72°C for 5 min, 98°C for 45 s, and 11 to 13 cycles of 98°C for 15 s, 60°C for 10 s, and 72°C for 1 min. Libraries were purified twice using 1× volume of AMPure XP Beads (Beckman Coulter; A63881) and resuspended in 15 μl of 0.1× tris-EDTA buffer. Following QC on a Bioanalyzer, libraries were multiplexed and sequenced (paired-end 150 bp) using a NovaSeq 6000 instrument (Illumina).

### STARR-seq plasmid library preparation

Genomic DNA was purified from cultured cells (Monarch Genomic DNA Purification Kit, NEB, T3010S). Two micrograms of purified DNA was sonicated, targeting an 800-bp peak (E220 Focused-ultrasonicator, Covaris), and subjected to double-sided AMPure XP bead (Beckman Coulter, A63881) size selection between ratios of 0.5× and 0.6×, aiming at a fragment size range between 700 and 900 bp. Fragment sizes before and after selection were analyzed on an agarose gel. Size-selected DNA was end repaired and ligated with the NEBNext hairpin adaptor (NEBNext Ultra II DNA Library Prep Kit for Illumina, NEB, E7645S, and NEBNext Multiplex Oligos for Illumina, NEB, E7335S). After adaptor ligation, the DNA was purified by 0.8× AMPure XP bead cleanup (Beckman Coulter, A63881) and eluted in 100 μl of buffer EB (QIAGEN, 19086) in total. To generate the insert library for cloning, the adaptor-ligated DNA was amplified with the STARR-seq library cloning forward (TAGAGCATGCACCGGACACTCTTTCCCTACACGACGCTCTTCCGATCT) and reverse (GGCCGAATTCGTCGAGTGACTGGAGTTCAGACGTGTGCTCTTCCGATCT) primers (*33*), which added a 15-bp homology sequence to both 3′ and 5′ ends. Twenty PCRs (program: 45 s at 98°C; eight cycles of 15 s at 98°C, 30 s at 65°C, and 45 s at 72°C; and 120 s at 72°C) were performed in total for all the adaptor-ligated DNA, where each 50-μl reaction contained 5 μl of the DNA, 2.5 μl of library cloning forward primer (10 μM), 2.5 μl of library cloning reverse primer (10 μM), 25 μl of KAPA 2× HiFi HotStart Ready Mix (Roche, KK2602), and 15 μl of nuclease-free water (Invitrogen, AM9938). The reactions were pooled, purified by 0.8× AMPure XP beads (Beckman Coulter, A63881), and eluted in 100 μl of buffer EB (QIAGEN, 19086) in total. Forty micrograms of hSTARR-seq_ORI vector (Addgene, 99296) was digested with 800 units of AgeI-HF (NEB, R3552L) and 800 units of SalI-HF (NEB, R3138L) in 16 of 50-μl reactions for 2 hours at 37°C, followed by deactivation for 20 min at 65°C. The reactions were size selected on an agarose gel for the ∼3000-bp band, and the selected plasmids were eluted from the gel using the QIAquick Gel Extraction kit (QIAGEN, 28704) into 400 μl of buffer EB (QIAGEN, 19086) in total. The eluted plasmids were pooled, purified by 1.8× AMPure XP beads (Beckman Coulter, A63881), and eluted in 50 μl of buffer EB (QIAGEN, 19086). To clone the insert library into the digested plasmid vector, 0.0667 pmol of vector and 0.1333 pmol of insert library were processed by each NEBuilder HiFi DNA Assembly (NEB, E2621L) reaction, where 40 reactions were performed in total. The reactions were pooled, purified by 1.8× AMPure XP beads (Beckman Coulter, A63881), and eluted in 30 μl of nuclease-free water (Invitrogen, AM9938). The cloned plasmid concentration was measured by a NanoDrop Spectrophotometer (Thermo Fisher Scientific). If the concentration was lower than 100 ng/μl, more cloning reactions were performed, and the products were pooled and concentrated by 1.8× AMPure XP bead (Beckman Coulter, A63881) purification. To transform MegaX DH10B T1R Electrocomp Cells (Invitrogen, C640003) with the cloned plasmids, 20 electroporation reactions were performed on the Gene Pulser II Electroporation System (Bio-Rad). One day before electroporation, 12 liters of LB media was prewarmed at 37°C; 10- and 20-μl pipette tips, 1.5-ml DNA LoBind tubes (Eppendorf, 0030108051), and Gene Pulser/MicroPulser Electroporation Cuvettes with a 0.1-cm gap (Bio-Rad, 1652083) were precooled to 4°C. One hour before electroporation, at least 24 ml of Recovery Media (provided in the MegaX DH10B T1R Electrocomp Cells kit, Invitrogen, C640003) was warmed up to 37°C. The following preparations were done in a 4°C cold room. At least 460 μl of bacteria was thawed on ice, and only bacteria with at most one freeze-thaw cycle were accepted for use. For each reaction, 100 ng of plasmids in less than 1 μl of nuclease-free water was added to 20 μl of bacteria, mixed by gently flicking the bottom of the tube a few times, and carefully transferred to a Gene Pulser/MicroPulser Electroporation Cuvette with a 0.1-cm gap (Bio-Rad, 1652083). The cuvette was tapped on the bench a few times to remove any air bubbles down the bottom of the cuvette and left on ice until electroporation. One pUC19 control reaction using 1 μl of pUC19 DNA (provided in the MegaX DH10B T1R Electrocomp Cells kit, Invitrogen, C640003) and 20 μl of bacteria was prepared in addition to 20 STARR-seq reactions. The cuvettes were then subjected to electroporation under the following settings on the Gene Pulser II Electroporation System (Bio-Rad; 2 kV, 25 μF, 200 Ω, 1 mm). Any result with a measured actual voltage of 2 ± 0.1 kV and a measured time constant of 4.9 ± 0.2 ms was regarded as a successful electroporation. After each electroporation reaction, 1 ml of prewarmed Recovery Medium (provided in the MegaX DH10B T1R Electrocomp Cells kit, Invitrogen, C640003) was immediately added to the cuvette. Electroporated bacteria were resuspended into the medium and transferred to a 14-ml polypropylene round-bottom tube (Falcon, 352059). The bacteria were recovered for 1 hour at 37°C with constant shaking at 200 rpm. All STARR-seq transformed bacteria were pooled. To estimate the transformation efficiency, 100 μl of 1:50, 1:500, and 1:5000 diluted STARR-seq transformed bacteria and 1:50 and 1:500 diluted pUC19 transformed bacteria was plated on selective LB agar plates containing ampicillin (100 μg/ml). After 16-hour incubation at 37°C, more than 50 colonies found on both the 1:5000 diluted STARR-seq plate and 1:500 diluted pUC19 plate indicated successful transformation. The rest of the pooled STARR-seq transformed bacteria were evenly added to 12 liters of prewarmed LB with ampicillin (100 μg/ml) and incubated for 12 to 14 hours at 37°C with constant shaking at 200 rpm. The bacterial culture was harvested by centrifugation, and the pellets were stored at −20°C. The pellets were purified using the QIAGEN Plasmid Plus Giga Kit (QIAGEN, 12991), eluted in 3 ml of buffer EB (QIAGEN, 19086), further purified and concentrated by 1.8× AMPure XP beads (Beckman Coulter, A63881), and eluted in nuclease-free water (Invitrogen, AM9938) at a final concentration of 5 μg/μl. To generate a sequencing library for the STARR-seq plasmid library, 10 PCRs (program: 45 s at 98°C; nine cycles of 15 s at 98°C, 30 s at 65°C, and 45 s at 72°C; 120 s at 72°C) were performed, where each 50-μl reaction contained 100 ng of plasmid library, 2.5 μl of Illumina i5 primer (10 μM), 2.5 μl of Illumina i7 primer (10 μM), 25 μl of KAPA 2× HiFi HotStart Ready Mix (Roche, KK2602), and nuclease-free water (Invitrogen, AM9938). The amplified sequencing libraries were pooled, purified by 0.8× AMPure XP beads (Beckman Coulter, A63881), and eluted in 20 μl of buffer EB (QIAGEN, 19086). Library concentration and quality were analyzed using the Agilent High Sensitivity DNA Kit (Agilent, 5067-4626) on a 2100 Bioanalyzer (Agilent).

### STARR-seq enhancer annotation

At least 250 million hiPSCs (HPSI0215i-yoch_6 or HPSI0214i-wibj_2) were prepared for each biological replicate. Cells were transfected in the MaxCyte R-1000 Processing Assemblies (MaxCyte, ER001M1-10) for the STARR-seq samples and in the R-50×3 Processing Assemblies (MaxCyte, ER050U3-10) for the control samples, both on the MaxCyte ATx electroporation platform (MaxCyte). Cells were harvested, washed with DPBS (Cytiva HyClone, SH30028.02), and counted for cell number and viability. Two hundred million cells were taken for the STARR-seq sample, and 10 million cells were taken for each of the three control samples: non-electroporated negative control, electroporated without DNA negative control, and green fluorescent protein (GFP)–transfected positive control (GFP-expressing plasmid; MaxCyte). Cells were centrifuged for 3 min at 300*g*, and the supernatant was removed. Cells of the non-electroporated negative control were directly plated in culture. Forty microliters of STARR-seq plasmid library at a concentration of 5 μg/μl was added to the STARR-seq sample cell pellet; 2 μl of GFP plasmid at a concentration of 5 μg/μl was added to the positive control cell pellet. Cells with or without plasmid DNA were then resuspended in MaxCyte Electroporation Buffer (provided in the Processing Assembly kits) to a final volume of 1 ml for the STARR-seq sample and 50 μl for the controls. The samples were then transferred to the corresponding Processing Assemblies (MaxCyte, ER001M1-10, and MaxCyte, ER050U3-10) and subjected to electroporation under the “optimization 7” program of ATx electroporation platform controlling software (MaxCyte). After electroporation, cells were transferred to uncoated culture vessels with excess surface area, aiming for maximum exposure to air, and recovered for 30 min at 37°C and 5% CO_2_. Recovered cells were then diluted to 1 million cells per ml of media with supplement and 10 μM ROCK inhibitor (Cell Guidance Systems, Y-27632) and cultured on vitronectin-coated (Thermo Fisher Scientific, A14700) vessels. For the STARR-seq sample, cells were cultured for 6 hours before being harvested for total RNA purification. For the control samples, cells were cultured for 24 hours and then in fresh media with supplement without ROCK inhibitor for another 24 hours before being harvested for flow cytometry analysis on GFP expression to determine the transfection efficiency of the experiment. For the STARR-seq sample, total RNA was purified from the harvested cells using the QIAGEN RNeasy Maxi Kit (QIAGEN, 75162) and eluted in three consecutive 1 ml of elutions in ribonuclease (RNase)–free water (provided in the RNeasy Maxi Kit). An aliquot of the total RNA was taken for integrity analysis using the Agilent RNA 6000 Pico Kit (Agilent, 5067-1513) on a 2100 Bioanalyzer (Agilent). The rest of the total RNA was stored at −80°C until sequencing library preparation. Any elution fractions with concentrations greater than 100 ng/μl were used for sequencing library preparation. mRNA was isolated from the total RNA using Dynabeads Oligo(dT)_25_ (Invitrogen, 61002) and eluted in 200 μl of buffer EB (QIAGEN, 19086). The isolated mRNA concentration was measured by a NanoDrop Spectrophotometer (Thermo Fisher Scientific). The mRNA was subjected to DNA digestion by TURBO deoxyribonuclease (DNase) (Invitrogen, AM2238). Each 100-μl reaction contained at most 20 μg of mRNA, 2.4 μl of TURBO DNase, and 1× TURBO DNase buffer (provided with the TURBO DNase). The reactions were pooled, purified by 1.8× RNAClean XP beads (Beckman Coulter, A63987), and eluted in 200 μl of nuclease-free water (Invitrogen, AM9938) in total. cDNA was synthesized from the mRNA by SuperScript IV Reverse Transcriptase (Invitrogen, 18090200), supplemented with Murine RNase Inhibitor (40,000 U/ml, NEB, M0314L). The number of reactions was determined by the mRNA yield according to 500 ng of mRNA per 20-μl reaction. The reverse transcriptase reactions were followed by RNase A treatment, where 0.2 μl of RNase A (10 mg/ml; Thermo Fisher Scientific, EN0531) was added to each reverse transcriptase reaction, and the mixture was incubated for 1 hour at 37°C. The reactions were purified by 1.8× AMPure XP beads (Beckman Coulter, A63881), eluted in 2 μl of buffer EB (QIAGEN, 19086) per reverse transcriptase reaction, and pooled. To enrich STARR-seq–specific cDNA, the purified cDNA was subjected to junction PCR, where one reaction amplified up to 20 μl of purified cDNA. Each 50-μl reaction (program: 45 s at 98°C; 16 cycles of 15 s at 98°C, 30 s at 65°C, and 60 s at 72°C; and 120 s at 72°C) contained the cDNA, 2.5 μl of junction forward primer (10 μM; TCGTGAGGCACTGGGCAG*G*T*G*T*C), 2.5 μl of junction reverse primer (10 μM; CTTATCATGTCTGCTCGA*A*G*C) (*33*), 25 μl of KAPA 2× HiFi HotStart Ready Mix (Roche, KK2602), and buffer EB (QIAGEN, 19086). The junction PCRs were purified by 0.8× AMPure XP beads (Beckman Coulter, A63881), eluted in 20 μl of buffer EB (QIAGEN, 19086) per reaction, and pooled. To generate the sequencing library, the junction PCR product was further subjected to sequencing-ready PCR. Each biological replicate used a unique set of 16 Illumina i5 and i7 dual-indexing combinations, and the junction PCR product was evenly split into 16 according to one reaction per dual-indexing combination. Each 50-μl reaction (program: 45 s at 98°C; eight cycles of 15 s at 98°C, 30 s at 65°C, and 45 s at 72°C; and 120 s at 72°C) contained the purified junction PCR product, the corresponding 2.5 μl of Illumina i5 primer (10 μM) and 2.5 μl of Illumina i7 primer (10 μM), 25 μl of KAPA 2× HiFi HotStart Ready Mix (Roche, KK2602), and buffer EB (QIAGEN, 19086). The reactions were pooled, purified by 0.8× AMPure XP beads (Beckman Coulter, A63881), and eluted in 40 μl of buffer EB (QIAGEN, 19086) in total. Library concentration and quality were analyzed using the Agilent High Sensitivity DNA Kit (Agilent, 5067-4626) on a 2100 Bioanalyzer (Agilent).

### Promoter Capture Hi-C

PCHi-C was performed as described (*38*) in the HipSci lines HPSI1113i-bima_1, HPSI0114i-kolf_2, HPSI0214i-kucg_2, HPSI1113i-podx_1, and HPSI1113i-qolg_1 (all Dpn II) and HPSI0114i-kolf_2, HPSI1113i-podx_1, and HPSI1113i-qolg_1 (all Hind III) with the following modifications: After Klenow-mediated fill-in of restriction fragment overhang (with biotin-2′-deoxyadenosine 5′-triphosphate, 2′-deoxycytidine 5′-triphosphate, 2′-deoxyguanosine 5′-triphosphate, and 3′-deoxythymidine 5′-triphosphate), nuclei were spun down for 5 min (2500 rpm at 4°C). The supernatant was removed, and the nucleus pellet was resuspended in 950 μl of 1× ligation buffer (supplied with T4 DNA ligase Invitrogen 15224-025) before 50 μl of T4 DNA ligase (Invitrogen 15224-025) was added for the ligation for 4 hours at 16°C.

### Luciferase assay plasmid preparation

Twenty-base homology sequences were added to both ends of the selected candidate sequences, positive and negative enhancer controls for cloning purposes (upstream: CTGGCCTAACTGGCCGGTAC; downstream: CCTCGAGGATATCAAGATCT). The combined sequences were artificially synthesized as gBlocks Gene Fragments (Integrated DNA Technologies). Minimal Promoter-Driven Firefly Luciferase Vector (pGL4.23[luc2/minP] Vector, Promega, E8411) and Promoterless Renilla Luciferase Basic Vectors (pGL4.70[hRluc] Vector, Promega, E6881) were amplified by transformation into NEB 5-alpha Competent *Escherichia coli* (High Efficiency) (NEB, C2987H) and minipreps using the QIAprep Spin Miniprep Kit (QIAGEN, 27104). For each candidate and positive and negative enhancer controls, 500 ng of empty firefly luciferase vector (pGL4.23) was restricted by KpnI-HF (NEB, R3142S) and NheI-HF (NEB, R3131S), purified by 0.8× AMPure XP beads (Beckman Coulter, A63881), and eluted in buffer EB (QIAGEN, 19086). To clone the gBlocks fragment insert into the digested plasmid vector, for each candidate, 0.0667 pmol of vector and 0.1333 pmol of insert were processed by NEBuilder HiFi DNA Assembly (NEB, E2621L) reaction. After the reaction was completed, 1 of the 20-μl reaction mixture was used to transform the cloned plasmid into 50 μl of NEB 5-alpha Competent *E. coli* (High Efficiency) (NEB, C2987H) by heat shock at 42°C for 30 s. After 5-min incubation on ice, the transformed bacteria were diluted in 1 ml of LB medium in a 14-ml polypropylene round-bottom tube (Falcon, 352059) and recovered for 1 hours at 37°C with constant shaking at 200 rpm. One microliter of the recovery mixture was then diluted in 100 μl of LB media and plated on a selective LB agar plate containing ampicillin (100 μg/ml). After 16-hour incubation at 37°C, a single colony was inoculated into 2 ml of LB media containing ampicillin (100 μg/ml) and incubated in a 14-ml polypropylene round-bottom tube (Falcon, 352059) for 12 to 14 hours at 37°C with constant shaking at 200 rpm. The amplified cloned plasmid was purified from the bacteria using the QIAprep Spin Miniprep Kit (QIAGEN, 27104) and eluted in 50 μl of buffer EB (QIAGEN, 19086). The plasmid was further purified and concentrated by 1.8× AMPure XP beads (Beckman Coulter, A63881) and eluted in nuclease-free water (Invitrogen, AM9938) at a final concentration of 1 μg/μl. To check the plasmid integrity, 100 ng of the empty firefly, *Renilla*, and all cloned candidate and control plasmids was restricted by ClaI (NEB, R0197S) and analyzed on an agarose gel.

### Luciferase assay

The minimum number of cells required for each biological replicate experiment was calculated as follows: 1 million hiPSCs per sample * total number of samples (the number of candidates + 4 controls). The four controls were no-DNA control, firefly luciferase empty plasmid control, positive enhancer control, and negative enhancer control. Cells were harvested, washed with DPBS (Cytiva HyClone, SH30028.02), and counted for cell number and viability. One million cells were transferred to an individual 14-ml polypropylene round-bottom tube (Falcon, 352059) for each of the samples and controls. Cells were centrifuged for 3 min at 300*g*, and the supernatant was removed. One microliter of candidate/control plasmid (1 μg/μl) and 1 μl of *Renilla* luciferase plasmid (1 μg/μl) were added to the corresponding cell pellet, except the no-DNA control. To transfect cells using the 4D-Nucleofector (Lonza) core and X units and the P3 Primary Cell 4D-Nucleofector X Kit L (Lonza, V4XP-3024), for each sample, cells and plasmid DNA were mixed (cells only for the no-DNA control) and resuspended in 100 μl of Nucleofection Solution (provided in the P3 kit, Lonza, V4XP-3024) and transferred to a Single Nucleocuvette (100 μl, provided in the P3 kit, Lonza, V4XP-3024). The cells were then subjected to the 4D-Nucleofector X Unit (Lonza) and electroporated using the CA137 program. After nucleofection, the cells were recovered within the Nucleocuvette at RT for 10 min, diluted in 1 ml of media with supplement and 10 μM ROCK inhibitor (Cell Guidance Systems, Y-27632), and cultured on one well of a vitronectin (Thermo Fisher Scientific, A14700)–coated 12-well plate for 24 hours. The media were then removed, and cells were washed on-plate using DPBS (Cytiva HyClone, SH30028.02) and lysed on-plate by adding 250 μl of 1× Passive lysis Buffer (provided in Dual-Luciferase Reporter Assay System, Promega, E1910) and incubating at RT for 15 min under constant rocking. The firefly and *Renilla* luciferase expression levels were measured by luminescence using the Dual-Luciferase Reporter Assay System (Promega, E1910) in wells of 96-well plates (96-well EIA/RIA Clear Flat Bottom Polystyrene High Bind Microplate, Individually Wrapped, without Lid, Nonsterile, Corning, 3590) in a FLUOstar Omega microplate reader (BMG Labtech).

### RNA-seq

One to 5 million cells were prepared for each biological replicate. Cells were harvested and washed with DPBS (Cytiva HyClone, SH30028.02). Total RNA was purified from the cell pellet using the Monarch Total RNA Miniprep Kit (NEB, T2010S). An aliquot of the total RNA was taken for integrity analysis using the Agilent RNA 6000 Pico Kit (Agilent, 5067-1513) on a 2100 Bioanalyzer (Agilent). The rest of the total RNA was stored at −80°C until sequencing library preparation. RNA-seq libraries were prepared using the NEBNext Ultra II Directional RNA Library Prep Kit for Illumina (NEB, E7760L), NEBNext Poly(A) mRNA Magnetic Isolation Module (NEB, E7490L), and NEBNext Multiplex Oligos for Illumina (Index Primers Set 1, 2, 3 and 4, NEB, E7335S, E7500S, E7710S, and E7730S), where, for each preparation, 500 ng of total RNA was used as the starting material and eight cycles were used for the final sequencing-ready PCR. Library concentration and quality were analyzed using the Agilent High Sensitivity DNA Kit (Agilent, 5067-4626) on a 2100 Bioanalyzer (Agilent). The STARR-seq sequencing libraries were sequenced in NextSeq500 HighOutput 75-bp Paired End runs and HiSeq2500-RapidRun 50-bp Paired End runs on corresponding sequencing platforms (Illumina). The RNA-seq sequencing libraries were sequenced in HiSeq2500-RapidRun 50-bp Paired End runs on the HiSeq sequencing platform (Illumina).

### H3K27ac ChIP-seq

ChIP-seq was performed as previously described (*91*) with minor modifications. Experiments were carried out in Yoch6 and KOLF2 iPSC lines using two biological replicates. Approximately 5 × 10^6^ cells were cross-linked with 1% formaldehyde for 10 min at RT, and the reaction was quenched by the addition of glycine to a final concentration of 125 mM. Cells were washed twice with ice-cold PBS, collected by centrifugation, and stored at −80°C until further processing.

Cross-linked chromatin was isolated by sequential lysis and fragmented using a Diagenode Bioruptor Plus Sonication System maintained at 4°C. Samples were sonicated for 30 cycles consisting of 30-s ON and 30-s OFF intervals, generating chromatin fragments predominantly between 200 and 500 bp. Following centrifugation, the soluble chromatin fraction was collected and used for immunoprecipitation.

For each immunoprecipitation, fragmented chromatin was incubated overnight at 4°C with 5 μg of anti-H3K27ac antibody (Abcam, ab4729) prebound to Dynabeads Protein G magnetic beads (Thermo Fisher Scientific, 10008D). Normal rabbit immunoglobulin G (Abcam, ab171870) was included as a negative control. Following immunoprecipitation, bead-bound complexes were washed with high-stringency radioimmunoprecipitation assay buffer and tris-buffered saline before elution. Cross-links were reversed by overnight incubation at 65°C, followed by RNase A and proteinase K treatment. DNA was purified using the DNA Clean & Concentrator kit (Zymo Research, D4004) and quantified using the Qubit High Sensitivity dsDNA assay (Thermo Fisher Scientific, Q32854).

Sequencing libraries were prepared using the NEBNext Ultra II DNA Library Prep Kit and NEBNext Multiplex Oligos for Illumina (New England Biolabs, E7645S and E7335S) according to the manufacturer’s instructions. Library quality and fragment size distribution were assessed using a High Sensitivity DNA assay (Agilent Technologies, 5067-4626) before paired-end sequencing on the Illumina NextSeq 2000 platform.

### Transient transcriptome sequencing (TT-seq)

TT-seq was performed as previously described (*92*) with minor modifications. hiPSCs were labeled with 500 μM 4-thiouridine (Sigma-Aldrich) for 20 min. Cells were counted and collected in TRIzol. RNA was chloroform extracted, DNase treated, and chloroform extracted. To 60 μg of total RNA was added 2× fragmentation buffer [final concentration: 75 mM tris-Cl (pH 8.3), 112.5 mM KCl, and 4.5 mM MgCl_2_] and heated to 95°C for 5 min. Fragmentation was stopped by adding EDTA to 50 mM and placing samples on ice. RNA was ethanol precipitated and resuspended in H_2_O. Fragment sizes were checked on the Bioanalyzer (peak size of ∼800 nucleotides). The biotinylation reaction was performed with MTSEA-Biotin XX (0.025 mg/ml; Biotum) in reaction buffer [20% *N*,*N*-dimethyl formamide, 1 mM EDTA, and 10 mM Hepes (pH 8)] for 45 min in the dark. Labeled RNA was chloroform extracted, ethanol precipitated, and resuspended in 90 μl of H_2_O. Seventy-five microliters of Dynabeads M-280 streptavidin (Thermo Fisher Scientific) was prewashed with Decon solution (0.1 M NaOH + 50 mM NaCl), 2× 100 mM NaCl, and 2× high-salt buffer [100 mM tris-Cl (pH 7.4), 10 mM EDTA (pH 8), 1 M NaCl, and 0.05% (v/v) Tween 20] and resuspended in high-salt buffer. Labeled RNA was denatured by heating it to 65°C for 5 min and placing it on ice for 2 min. Ten microliters of high-salt buffer was added to labeled RNA. Prewashed beads were placed on the magnet, the supernatant was discarded, and then beads were resuspended in the labeled RNA. Beads and RNA were rotated for 30 min in the dark. Beads were then washed 4× for 1 min with high-salt buffer. Labeled RNA was eluted 2× with 100 mM 1,4-dithiothreitol. RNA was cleaned with a RNeasy MinElute Clean-up Kit (Qiagen cat. no 74204). Two hundred fifty nanograms of RNA was used for library preparation using the CORALL total RNA-seq library preparation kit (Lexogen cat. no. 147.24) using eight cycles of PCR amplification. Libraries were sequenced in a NovaSeq 6000 (Novogene) with 150-bp paired-end reads.

### Immunofluorescence

HiPSC colonies were cultured on 16-mm glass coverslips (Fisherbrand, 12323148) placed in 12-well plates. After the induction period, the colonies were washed with PBS and then fixed in 4% paraformaldehyde for 20 min at RT. Following three washes with 1× PBS, the colonies were incubated in a permeabilization and blocking buffer (PB; 1% bovine serum albumin and 0.1% Triton X-100 in PBS) for 30 min at RT. The cells were incubated overnight at 4°C with primary antibodies against SOX2 (Santa Cruz, sc-365823, 1:500) and NANOG (R&D, AF1997, 1:100) in PB. The following day, they were incubated for 1 hour at RT with fluorescence-conjugated secondary antibodies (Thermo Fisher Scientific, A32787 and A11057, 1:400), followed by three washes with 1× PBS. Last, the colonies were mounted with ProLong Glass Antifade Mountant with NucBlue Stain (Thermo Fisher Scientific, P36983) and allowed to dry. The samples were imaged using a Leica Stellaris 8 confocal microscope with LAS X software and analyzed with CellProfiler 4.2.6 and ImageJ 1.54f software.

### Live-cell imaging

Live-cell imaging was performed using the Sartorius Incucyte S5 system, and time-lapse images and videos were analyzed using the Sartorius Incucyte 2022B Rev2 software interface.

### qRT-PCR

Total RNA was extracted using the Monarch Total RNA Miniprep Kit (NEB, T2010S), and 1 μg of RNA was used for complementary DNA synthesis with the RevertAid First Strand cDNA Synthesis Kit (Thermo Fisher Scientific, K1622) according to the manufacturer’s protocols. Quantitative PCR (qPCR) was performed on a CFX Opus 96 Real-Time PCR System (Bio-Rad) using specific primers for target genes (table S7) and quantitative reverse transcription PCR (qRT-PCR) Brilliant III SYBR Master Mix (Agilent, 600886). The qPCR cycle conditions were as follows: 40 cycles of 10 s at 95°C, 30 s at 55°C, and 30 s at 72°C. Primers were designed using NCBI Primer3 and synthesized by Integrated DNA Technologies. Quantifications were calculated and normalized to the endogenous glyceraldehyde-3-phosphate dehydrogenase (*GAPDH*) internal control. The ΔΔCt method was used for relative quantification of each target gene compared to an internal control.

### CRISPRa

We designed single guide RNA (sgRNA) and used CRISPRa plasmids on the basis of the DOX-inducible catalytically inactive Cas9 (dCas9), as described previously (*16*). Three sgRNAs targeting the enhancer regions were designed using the gRNA design tool of Integrated DNA Technologies (table S8). A total of 1.5 μg of each of the piggyBac-based CRISPRa (puromycin-resistant cassette; Addgene, 183409), sgRNA plasmids (neomycin-resistant cassette; Addgene, 183411), and a hyperactive piggyBac transposase plasmid was cotransfected into hiPSC lines using the Invitrogen Neon Transfection System. An increasing concentration of puromycin (up to 1 μg/ml) and neomycin (up to 200 μg/ml) was used for selection of transgenic cells for 7 to 10 days. To induce the CRISPRa system and activation of the enhancers, DOX was used at a 0.5 μg/ml concentration in TeSR-E8 medium for 4 days, and cells were collected for further analysis after the induction period. Noninduced cells (without DOX) were used as a negative control.

### CRISPRi

CRISPRi was carried out as previously described (*16*). A total of 1.5 μg of piggyBac-based CRISPRi plasmid (Addgene, 183410), three sgRNA plasmids for each region (table S8), and piggyBac-transposase plasmid was cotransfected into hiPSCs using the Invitrogen Neon Transfection System. Stable cell lines were then selected with increasing concentrations of puromycin and neomycin for 7 to 10 days after transfection. To test the effect of enhancer perturbation on the expression of target gene(s), CRISPRi hiPSC lines were induced with DOX (0.5 μg/ml) and 10 μM trimethoprim (TMP) to induce and stabilize dCas9-KRAB for 4 days and collected for further analysis. Noninduced cells (without DOX and TMP) were used as a negative control.

### Generation of enhancer knockout hiPSC lines

To test the functionality of selected primed enhancers, we deleted a segment within an annotated ATAC+/STARR+ region. We cloned sgRNAs targeting two regions of each enhancer tested (table S9) into the pSpCas9(BB)-2A-Puro (PX459) plasmid (Addgene plasmid no. 48139), which expresses SpCas9 and contains a puromycin resistance cassette. The sgRNA plasmids were introduced into the Yoch6 hiPSC line through lipofection. Before transfection, hiPSCs were cultured in StemFlex medium (Gibco, A33494-01) for at least two passages. A day before lipofection, 100,000 cells were seeded per well of a Geltrex-coated (Gibco, A14133-02) 12-well plate. On the day of transfection, the spent medium was aspirated and replaced with OptiMEM medium (Gibco, 11058-021) supplemented with 10 μM ROCK inhibitor Y27632 (Tocris Bioscience, 1254). Lipofectamine STEM (Thermo Fisher Scientific, STEM00001) was mixed with 500 ng of each sgRNA plasmid construct, added onto cells, and allowed to incubate at 37°C for 4 hours. The wells were then topped up with StemFlex medium and left to incubate at 37°C overnight. Transfected cells were selected by adding puromycin (0.25 μg/ml) for ∼24 to 48 hours. Surviving cells were allowed to grow into hiPSC colonies, picked out with a wide-bore pipette tip, seeded into Geltrex-coated 96-well plates, and genotyped using the Phusion polymerase (NEB, M0530) with specific primers (table S9). To quantify enhancer target gene expression, total RNA was extracted from snap-frozen pellets using the Quick-RNA Tissue MicroPrep kit (Zymo Research, R2030) or the QIAGEN RNeasy Micro Kit (QIAGEN, 74004) according to the manufacturer’s instructions. After assessing RNA quality and concentration (NanoDrop), cDNA was synthesized with the QuantiTect Reverse Transcription Kit (QIAGEN, 205313). qPCR was then performed on a CFX Opus System (Bio-Rad) using the KAPA SYBR FAST Mix (Roche, SFUKB) and specific primers (table S7). Gene expression levels were quantified using the ΔΔCt method, and statistical significance was assessed using Student’s *t* test.

### ddPCR assay

Copy number variation and knockout efficiency were measured by ddPCR and performed as described previously (*93*) with minor modifications. Genomic DNA of different timelines of knockout cells was added to the PCR mixture containing 2× no dUTP ddPCR Supermix for probes (Bio-Rad Laboratories, 1863023), 900 nM target-specific PCR primers (table S7), and 250 nM LNA TaqMan probes at a total volume of 20 μl. The probes were designed and purchased from Integrated DNA Technologies Inc. (US) and labeled with 6-carboxyfluorescein on the SLC13A4 enhancer region and hexachloro-6-carboxyfluorescein on the 5′ end of SRY gene and Iowa Black FQ quencher at the 3′ end. Droplet generation was performed by adding the PCR mixture to 70 μl of droplet generation oil for probes (Bio-Rad Laboratories, 1863005). Droplets were produced with a QX200 Droplet Generator (Bio-Rad Laboratories, US) according to the manufacturer’s manual. The droplet emulsion was thermally cycled as follows: denaturing at 95°C for 10 min, 40 cycles of PCR at 94°C for 30 s, 60°C for 1 min, and a final extension at 98°C for 10 min. The products were scanned on a QX200 Droplet Reader (Bio-Rad Laboratories, US), and the data were analyzed in QuantaSoft version 1.7.4.0917 software (Bio-Rad Laboratories, US). Thresholds between positive and negative droplet signals were manually adjusted to exclude false-positive droplets and normalized to the SRY gene, of which there is only one copy in the genome.

### RNA-seq data processing and analyses

RNA-seq data analysis and quality check of the raw reads were done by FastQC version 0.11.8 (www.bioinformatics.babraham.ac.uk/projects/fastqc/), and subsequently, reads were aligned to the human reference genome GRCh38 using STAR version 2.5.2b2 (*94*). Duplicates were removed using Picard (version 2.9.0) (https://broadinstitute.github.io/picard/). Aligned reads were assigned to gene annotation using HTSeq-count version 0.10 (*95*). Differential analysis and normalization were performed using DESeq2 (*96*). Genes were considered differentially expressed if they had an absolute log_2_ fold change exceeding 1 and a *P*_adj_ of less than 0.01. Principal components analysis was performed by the prcomp function and plotted by ggplot2 in R (*97*). Heatmaps were generated using the pheatmap R package (https://rdrr.io/cran/pheatmap/). Gene Ontology enrichment analysis was performed using clusterProfiler (*98*). Differences in RNA-seq signal between enhancer categories were assessed using two-sided Mann-Whitney *U* tests, and *P* values were corrected for multiple testing using the Benjamini-Hochberg procedure.

### TT-seq data processing and analyses

TT-seq data analysis and quality check of the raw reads were done by FastQC version 0.11.8 (www.bioinformatics.babraham.ac.uk/projects/fastqc/), and subsequently, reads were aligned to the human reference genome GRCh38 using STAR version 2.5.2b2 (*94*). The BAM files generated for biological replicates were merged with SAMtools and merged and filtered for only uniquely mapped reads using the SAMtools flag -q 255, followed by sorting and indexing. Coverage tracks requiring bigWig files and heatmaps were generated using deepTools version 3.3.1 (*99*).

### ATAC-seq data processing and analyses

ATAC-seq data analysis and quality inspection were performed using FastQC version 0.11.5 (www.bioinformatics.babraham.ac.uk/projects/fastqc/). Nextera Transposase adapter and low-quality bases were eliminated using Cutadapt version 1.17 (*100*). After trimming, reads were mapped to human genome GRCh38 assembly using bowtie2 version 2.3.4.2 (*101*). Mapped pairs were further filtered to maintain the mapping quality above 20 using custom scripts and SAMtools version 1.9 (*102*). PCR duplicates and mitochondrial reads were removed by Picard (https://broadinstitute.github.io/picard/) and SAMtools as well as reads blacklisted by The Encyclopedia of DNA Elements (ENCODE) project (*103*). Peaks of accessible chromatin were identified for each sample using MACS2 version 2.1.2 (*104*) with the following settings: -f BEDPE --keep-dup (51). Coverage tracks requiring bigWig files and heatmaps were generated using deepTools version 3.3.1 (*99*).

### CUT&Tag data processing and analyses

CUT&Tag datasets were processed and mapped to the human reference genome GRCh38, as described previously (*36*). After mapped reads were further filtered to maintain the mapping quality above 30 using custom scripts and SAMtools version 1.9 (*102*), peaks were called using MACS2 version 2.1.2 (*104*). H3K9me3 peaks were called using the following settings: -broad -f BAMPE --keepdup all -p 1e-2. BigWig files required for coverage tracks and heatmaps were generated using deepTools version 3.3.1 (*99*).

### ChIP-seq data processing and analyses

ChIP-seq datasets were mapped to the human reference genome GRCh38 using bowtie2 version 2.3.4.2 (*101*). After mapped reads were further filtered to maintain the mapping quality above 30 using custom scripts and SAMtools version 1.9 (*102*), peaks were called using MACS2 version 2.1.2 (*104*) with the following settings: narrowPeak representation was used for histone marks H3K4me3 and H3K27ac with the following settings: -f BAMPE --keepdup all -p 1e-2. H3K27me3 and H3K9me3 peaks were called using the following settings: -broad -f BAMPE --keepdup all -p 1e-2. BigWig files required for coverage tracks and heatmaps were generated using deepTools version 3.3.1 (*99*). To separate enhancers into lineage-restricted and multilineage categories, we used a master set of peaks combined from several published ChIP-seq H3K27ac datasets (ME, TBL, MSC, and NPC) from SRP000941. We called the peaks separately on the merged replicates in the respective studies. BigWig files required for coverage tracks and heatmaps were generated using deepTools version 3.3.1 (*99*). H3K27ac hiPSC Yoch6 peaks and H3K27ac hESC peaks (*56*) were combined, and overlapping or adjacent intervals merged into a consensus peak set using reduce() from the GenomicRanges package in R.

### STARR-seq data processing and analyses

Reads of input and output STARR-seq libraries were aligned to human genome GRCh38 by bowtie2. Nonunique alignments and alignments with mapping quality less than 30 were filtered out. Duplicates were removed using Picard (version 2.9.0) (https://broadinstitute.github.io/picard/). For each output replicate, uniquely mapped distinct fragments from 16 different indexing libraries were pooled and included in the downstream analysis. Duplicated fragments from different indexing libraries were treated as biological duplicates. STARR-seq biological replicates were merged using SAMtools merge before peak calling. STARR-seq peaks were called using STARRpeaker using default settings. BigWig files required for coverage tracks and heatmaps were generated using deepTools version 3.3.1 (*99*). Differences in STARR-seq enhancer activity (enhancer strength) between enhancer categories were assessed using two-sided Mann-Whitney *U* tests with Benjamini-Hochberg correction for multiple testing. Enhancers were annotated on the basis of the center of each called peak.

### PCHi-C data processing and analyses

Dpn II and Hind III PCHi-C data were mapped and filtered using HiCUP (*51*) with the GRCh38 human genome build. BAM files were converted to a CHiCAGO-compatible format (.chinput) using the bam2chicago script. CHiCAGO (*52*) was used to define significant promoter interactions at the level of individual fragments. CHiCAGO uses a convolution background model for the background level of interactions for pooled baited and other regions and a weighted distance–dependent multiple testing correction.

Two biological replicates for each of five different cell lines were normalized and combined as part of the CHiCAGO pipeline. CHiCAGO interaction scores correspond to –log-transformed, weighted *P* values for each fragment read pair. A CHiCAGO interaction score of 5 or above was considered significant on the basis of previous empirical observations (*52*). We used default CHiCAGO weights for Hind III data processing. To process Dpn II data, we applied the fitdistcurve function from the CHiCAGO package to measure optimal weight parameters. PCHi-C contacts were visualized using WashU Browser (https://epigenomegateway.wustl.edu).

### Identification of interallelic enhancer variants

SNP calling for allelic imbalance in STARR-seq data was performed following the GATK pipeline as recommended by the GATK Best Practices (software.broadinstitute.org/gatk/best-practices/). To begin with, all raw FASTQ files for each of the STARR-seq replicate were first converted to BAM format using the FastqToSam program. The BAM files were combined using SAMtools merge and sorted by query name. MarkIlluminaAdapters was used to mark PCR duplicates, and marked BAM files were converted to interleaved FASTQ format using the SamToFastq program. FASTQ-to-BAM conversion was then performed using BWA-MEM software and recalibrated after removing PCR duplicates. Haplotype calling was performed on each of the recalibrated BAM files using HaplotypeCaller in discovery mode and combined using the CombineGVCFs program. Next, recalibration of SNPs and indels was carried out using the VariantRecalibrator function using HapMap version 3.3 (priority = 15), 1000G_omni version 2.5 (priority = 12), Broad Institute 1000G high-confidence SNP list phase 1 (priority = 10), Mills 1000G golden standard INDEL list (priority = 12), and dbSNP version 150 (priority = 2) databases. Postidentification of SNPs that show allelic imbalance, the N-masked genome was generated using the SNPsplit_genome_preparation program. FASTQ files were then aligned to the N-masked genome, and SNPsplit was used to separate reads into Allele1 and Allele2. SAMtools pileup was further used to count read pileup at heterozygous SNP sites. Binomial testing was used to identify regions that show a significant (*P*_adj_ < 0.05) difference in pileups between Allele1 and Allele2.

### Motif discovery and enrichment analysis

The JASPAR 2022 core nonredundant motifs were used as the initial motif candidate list. Only transcription factor motifs that had at least 10 TPM (transcripts per million) levels of expression were selected for motif discovery analysis. AME (*105*) from MEME Suite (version 5.5.1) (*106*) was used to perform motif discovery and enrichment analysis. AME identifies known motifs that are relatively enriched in given sequences compared with control sequences.

### Prediction of enhancer variant effects on histone marks

AlphaGenome (Google DeepMind) (*61*) was used to predict the effect of each SNP on histone modification signal in H1 hESCs. For each variant, predictions were generated within a 16-kb window centered on the SNP, comparing reference and alternative alleles across histone modifications. For each variant-mark combination, a raw effect score was computed as the difference in predicted signal between alleles, and a corresponding signed quantile score was derived to indicate the extremity of the predicted effect relative to a background distribution of variants. Results are visualized as a heatmap, with tile color representing the raw score and text annotations showing both the raw score and signed quantile score per cell.

### DeepSTARR

DeepSTARR is a deep-learning model that directly predicts the activity of enhancers in DNA sequences (*49*). To train and apply the model, we performed the pipeline as described previously (*49*), with minor changes. We only included bins with more than five reads in the input and at least one read in the STARR-seq output. The genome was binned into regions of 499 bp with a stride of 100 bp. The included bins came from the following:

1) Random regions of accessible chromatin with low STARR-seq signal.

2) Random regions of accessible chromatin with high STARR-seq signal.

3) Random regions of inaccessible chromatin with various STARR-seq signals.

We augmented our dataset by adding the reverse complement of each original sequence with the same output. The model and performance evaluation were conducted as previously described (*49*), with the exception of changing the input DNA sequence length to 499 bp.

### Published ChIP-seq data

All published data used in the analysis are available to download using following accession numbers: SRP000941 and GSE69647 for hESC H3K27ac; GSE59434 for hESC H3K27me3 and H3K4me3; GSE75868 for hESC H3K9me3; GSE19465 for hESC H3K4me1; GSE51334 for hESC P300 and POLR2A; GSE16256 for hESC, MSC, NPC, TBL, and ME H3K27ac, hESC H2BK5ac, H2BK12ac, and H4K91ac; GSE290515 for hiPSC ATAC-seq data; and GSE314408 for hiPSC RNA-seq data.

## Supplementary Materials

### The PDF file includes:

Figs. S1 to S6 Legends for tables S1 to S9 Legends for movies S1 to S6 References

## References

[1] D. Shlyueva, G. Stampfel, A. Stark, Transcriptional enhancers: From properties to genome-wide predictions. Nat. Rev. Genet. 15, 272–286 (2014). 10.1038/nrg3682 24614317

[2] S. Kim, J. Wysocka, Deciphering the multi-scale, quantitative cis-regulatory code. Mol. Cell 83, 373–392 (2023). 10.1016/j.molcel.2022.12.032 PMC9898153 36693380

[3] E. E. M. Furlong, M. Levine, Developmental enhancers and chromosome topology. Science 361, 1341–1345 (2018). 10.1126/science.aau0320 PMC6986801 30262496

[4] S. Schoenfelder, P. Fraser, Long-range enhancer–promoter contacts in gene expression control. Nat. Rev. Genet. 20, 437–455 (2019). 10.1038/s41576-019-0128-0 31086298

[5] ENCODE Project Consortium, An integrated encyclopedia of DNA elements in the human genome. Nature 489, 57–74 (2012). 10.1038/nature11247 PMC3439153 22955616

[6] T. Sagai, M. Hosoya, Y. Mizushina, M. Tamura, T. Shiroishi, Elimination of a long-range cis-regulatory module causes complete loss of limb-specific Shh expression and truncation of the mouse limb. Development 132, 797–803 (2005). 10.1242/dev.01613 15677727

[7] D. G. Lupiáñez, K. Kraft, V. Heinrich, P. Krawitz, F. Brancati, E. Klopocki, D. Horn, H. Kayserili, J. M. Opitz, R. Laxova, F. Santos-Simarro, B. Gilbert-Dussardier, L. Wittler, M. Borschiwer, S. A. Haas, M. Osterwalder, M. Franke, B. Timmermann, J. Hecht, M. Spielmann, A. Visel, S. Mundlos, Disruptions of topological chromatin domains cause pathogenic rewiring of gene-enhancer interactions. Cell 161, 1012–1025 (2015). 10.1016/j.cell.2015.04.004 PMC4791538 25959774

[8] N. Gonen, C. R. Futtner, S. Wood, S. A. Garcia-Moreno, I. M. Salamone, S. C. Samson, R. Sekido, F. Poulat, D. M. Maatouk, R. Lovell-Badge, Sex reversal following deletion of a single distal enhancer of Sox9. Science 360, 1469–1473 (2018). 10.1126/science.aas9408 PMC6034650 29903884

[9] M. T. Maurano, R. Humbert, E. Rynes, R. E. Thurman, E. Haugen, H. Wang, A. P. Reynolds, R. Sandstrom, H. Qu, J. Brody, A. Shafer, F. Neri, K. Lee, T. Kutyavin, S. Stehling-Sun, A. K. Johnson, T. K. Canfield, E. Giste, M. Diegel, D. Bates, R. S. Hansen, S. Neph, P. J. Sabo, S. Heimfeld, A. Raubitschek, S. Ziegler, C. Cotsapas, N. Sotoodehnia, I. Glass, S. R. Sunyaev, R. Kaul, J. A. Stamatoyannopoulos, Systematic localization of common disease-associated variation in regulatory DNA. Science 337, 1190–1195 (2012). 10.1126/science.1222794 PMC3771521 22955828

[10] E. Smith, A. Shilatifard, Enhancer biology and enhanceropathies. Nat. Struct. Mol. Biol. 21, 210–219 (2014). 10.1038/nsmb.2784 24599251

[11] A. Claringbould, J. B. Zaugg, Enhancers in disease: Molecular basis and emerging treatment strategies. Trends Mol. Med. 27, 1060–1073 (2021). 10.1016/j.molmed.2021.07.012 34420874

[12] N. A. Kearns, H. Pham, B. Tabak, R. M. Genga, N. J. Silverstein, M. Garber, R. Maehr, Functional annotation of native enhancers with a Cas9–histone demethylase fusion. Nat. Methods 12, 401–403 (2015). 10.1038/nmeth.3325 PMC4414811 25775043

[13] B. Bonev, N. Mendelson Cohen, Q. Szabo, L. Fritsch, G. L. Papadopoulos, Y. Lubling, X. Xu, X. Lv, J.-P. Hugnot, A. Tanay, G. Cavalli, Multiscale 3D genome rewiring during mouse neural development. Cell 171, 557–572.e24 (2017). 10.1016/j.cell.2017.09.043 PMC5651218 29053968

[14] Z. Chen, V. Snetkova, G. Bower, S. Jacinto, B. Clock, A. Dizehchi, I. Barozzi, B. J. Mannion, A. Alcaina-Caro, J. Lopez-Rios, D. E. Dickel, A. Visel, L. A. Pennacchio, E. Z. Kvon, Increased enhancer–promoter interactions during developmental enhancer activation in mammals. Nat. Genet. 56, 675–685 (2024). 10.1038/s41588-024-01681-2 PMC11203181 38509385

[15] P. Liu, M. Chen, Y. Liu, L. S. Qi, S. Ding, CRISPR-based chromatin remodeling of the endogenous Oct4 or Sox2 locus enables reprogramming to pluripotency. Cell Stem Cell 22, 252–261.e4 (2018). 10.1016/j.stem.2017.12.001 29358044

[16] W. W. C. Tang, A. Castillo-Venzor, W. H. Gruhn, T. Kobayashi, C. A. Penfold, M. D. Morgan, D. Sun, N. Irie, M. A. Surani, Sequential enhancer state remodelling defines human germline competence and specification. Nat. Cell Biol. 24, 448–460 (2022). 10.1038/s41556-022-00878-z PMC7612729 35411086

[17] J. Banerji, S. Rusconi, W. Schaffner, Expression of a beta-globin gene is enhanced by remote SV40 DNA sequences. Cell 27, 299–308 (1981). 10.1016/0092-8674(81)90413-x 6277502

[18] A. Visel, M. J. Blow, Z. Li, T. Zhang, J. A. Akiyama, A. Holt, I. Plajzer-Frick, M. Shoukry, C. Wright, F. Chen, V. Afzal, B. Ren, E. M. Rubin, L. A. Pennacchio, ChIP-seq accurately predicts tissue-specific activity of enhancers. Nature 457, 854–858 (2009). 10.1038/nature07730 PMC2745234 19212405

[19] N. D. Heintzman, G. C. Hon, R. D. Hawkins, P. Kheradpour, A. Stark, L. F. Harp, Z. Ye, L. K. Lee, R. K. Stuart, C. W. Ching, K. A. Ching, J. E. Antosiewicz-Bourget, H. Liu, X. Zhang, R. D. Green, V. V. Lobanenkov, R. Stewart, J. A. Thomson, G. E. Crawford, M. Kellis, B. Ren, Histone modifications at human enhancers reflect global cell-type-specific gene expression. Nature 459, 108–112 (2009). 10.1038/nature07829 PMC2910248 19295514

[20] M. P. Creyghton, A. W. Cheng, G. G. Welstead, T. Kooistra, B. W. Carey, E. J. Steine, J. Hanna, M. A. Lodato, G. M. Frampton, P. A. Sharp, L. A. Boyer, R. A. Young, R. Jaenisch, Histone H3K27ac separates active from poised enhancers and predicts developmental state. Proc. Natl. Acad. Sci. U.S.A. 107, 21931–21936 (2010). 10.1073/pnas.1016071107 PMC3003124 21106759

[21] A. Rada-Iglesias, R. Bajpai, T. Swigut, S. A. Brugmann, R. A. Flynn, J. Wysocka, A unique chromatin signature uncovers early developmental enhancers in humans. Nature 470, 279–283 (2011). 10.1038/nature09692 PMC4445674 21160473

[22] V. Sartorelli, S. M. Lauberth, Enhancer RNAs are an important regulatory layer of the epigenome. Nat. Struct. Mol. Biol. 27, 521–528 (2020). 10.1038/s41594-020-0446-0 PMC7343394 32514177

[23] T. S. Barakat, F. Halbritter, M. Zhang, A. F. Rendeiro, E. Perenthaler, C. Bock, I. Chambers, Functional dissection of the enhancer repertoire in human embryonic stem cells. Cell Stem Cell 23, 276–288.e8 (2018). 10.1016/j.stem.2018.06.014 PMC6084406 30033119

[24] A. Visel, S. Minovitsky, I. Dubchak, L. A. Pennacchio, VISTA Enhancer Browser—A database of tissue-specific human enhancers. Nucleic Acids Res. 35, D88–D92 (2007). 10.1093/nar/gkl822 PMC1716724 17130149

[25] D. U. Gorkin, I. Barozzi, Y. Zhao, Y. Zhang, H. Huang, A. Y. Lee, B. Li, J. Chiou, A. Wildberg, B. Ding, B. Zhang, M. Wang, J. S. Strattan, J. M. Davidson, Y. Qiu, V. Afzal, J. A. Akiyama, I. Plajzer-Frick, C. S. Novak, M. Kato, T. H. Garvin, Q. T. Pham, A. N. Harrington, B. J. Mannion, E. A. Lee, Y. Fukuda-Yuzawa, Y. He, S. Preissl, S. Chee, J. Y. Han, B. A. Williams, D. Trout, H. Amrhein, H. Yang, J. M. Cherry, W. Wang, K. Gaulton, J. R. Ecker, Y. Shen, D. E. Dickel, A. Visel, L. A. Pennacchio, B. Ren, An atlas of dynamic chromatin landscapes in mouse fetal development. Nature 583, 744–751 (2020). 10.1038/s41586-020-2093-3 PMC7398618 32728240

[26] B. J. Mannion, S. Tran, I. Plajzer-Frick, C. S. Novak, V. Afzal, J. A. Akiyama, I. Sospedra-Arrufat, S. Barton, E. Beckman, T. H. Garvin, P. Godfrey, J. Godoy, R. D. Hunter, M. Kato, M. Kosicki, A. N. Kronshage, E. A. Lee, E. M. Meky, Q. T. Pham, K. von Maydell, Y. Zhu, J. Lopez-Rios, D. E. Dickel, M. Osterwalder, A. Visel, L. A. Pennacchio, Uncovering hidden enhancers through unbiased in vivo testing. Nat. Commun. 16, 7313 (2025). 10.1038/s41467-025-62497-0 PMC12331988 40775209

[27] M. Kosicki, F. A. Baltoumas, G. Kelman, J. Boverhof, Y. Ong, L. E. Cook, D. E. Dickel, G. A. Pavlopoulos, L. A. Pennacchio, A. Visel, VISTA Enhancer browser: An updated database of tissue-specific developmental enhancers. Nucleic Acids Res. 53, D324–D330 (2025). 10.1093/nar/gkae940 PMC11701537 39470740

[28] A. Sankar, F. Mohammad, A. K. Sundaramurthy, H. Wang, M. Lerdrup, T. Tatar, K. Helin, Histone editing elucidates the functional roles of H3K27 methylation and acetylation in mammals. Nat. Genet. 54, 754–760 (2022). 10.1038/s41588-022-01091-2 35668298

[29] T. Zhang, Z. Zhang, Q. Dong, J. Xiong, B. Zhu, Histone H3K27 acetylation is dispensable for enhancer activity in mouse embryonic stem cells. Genome Biol. 21, 45 (2020). 10.1186/s13059-020-01957-w PMC7035716 32085783

[30] M. M. Pradeepa, G. R. Grimes, Y. Kumar, G. Olley, G. C. A. Taylor, R. Schneider, W. A. Bickmore, Histone H3 globular domain acetylation identifies a new class of enhancers. Nat. Genet. 48, 681–686 (2016). 10.1038/ng.3550 PMC4886833 27089178

[31] N. Rajagopal, S. Srinivasan, K. Kooshesh, Y. Guo, M. D. Edwards, B. Banerjee, T. Syed, B. J. M. Emons, D. K. Gifford, R. I. Sherwood, High-throughput mapping of regulatory DNA. Nat. Biotechnol. 34, 167–174 (2016). 10.1038/nbt.3468 PMC5108523 26807528

[32] Y. Diao, R. Fang, B. Li, Z. Meng, J. Yu, Y. Qiu, K. C. Lin, H. Huang, T. Liu, R. J. Marina, I. Jung, Y. Shen, K.-L. Guan, B. Ren, A tiling-deletion-based genetic screen for cis-regulatory element identification in mammalian cells. Nat. Methods 14, 629–635 (2017). 10.1038/nmeth.4264 PMC5490986 28417999

[33] C. D. Arnold, D. Gerlach, C. Stelzer, Ł. M. Boryń, M. Rath, A. Stark, Genome-wide quantitative enhancer activity maps identified by STARR-seq. Science 339, 1074–1077 (2013). 10.1126/science.1232542 23328393

[34] J. D. Buenrostro, P. G. Giresi, L. C. Zaba, H. Y. Chang, W. J. Greenleaf, Transposition of native chromatin for fast and sensitive epigenomic profiling of open chromatin, DNA-binding proteins and nucleosome position. Nat. Methods 10, 1213–1218 (2013). 10.1038/nmeth.2688 PMC3959825 24097267

[35] B. Schwalb, M. Michel, B. Zacher, K. Frühauf, C. Demel, A. Tresch, J. Gagneur, P. Cramer, TT-seq maps the human transient transcriptome. Science 352, 1225–1228 (2016). 10.1126/science.aad9841 27257258

[36] H. S. Kaya-Okur, S. J. Wu, C. A. Codomo, E. S. Pledger, T. D. Bryson, J. G. Henikoff, K. Ahmad, S. Henikoff, CUT&Tag for efficient epigenomic profiling of small samples and single cells. Nat. Commun. 10, 1930 (2019). 10.1038/s41467-019-09982-5 PMC6488672 31036827

[37] B. Mifsud, F. Tavares-Cadete, A. N. Young, R. Sugar, S. Schoenfelder, L. Ferreira, S. W. Wingett, S. Andrews, W. Grey, P. A. Ewels, B. Herman, S. Happe, A. Higgs, E. LeProust, G. A. Follows, P. Fraser, N. M. Luscombe, C. S. Osborne, Mapping long-range promoter contacts in human cells with high-resolution capture Hi-C. Nat. Genet. 47, 598–606 (2015). 10.1038/ng.3286 25938943

[38] S. Schoenfelder, M. Furlan-Magaril, B. Mifsud, F. Tavares-Cadete, R. Sugar, B.-M. Javierre, T. Nagano, Y. Katsman, M. Sakthidevi, S. W. Wingett, E. Dimitrova, A. Dimond, L. B. Edelman, S. Elderkin, K. Tabbada, E. Darbo, S. Andrews, B. Herman, A. Higgs, E. LeProust, C. S. Osborne, J. A. Mitchell, N. M. Luscombe, P. Fraser, The pluripotent regulatory circuitry connecting promoters to their long-range interacting elements. Genome Res. 25, 582–597 (2015). 10.1101/gr.185272.114 PMC4381529 25752748

[39] C. Neumayr, M. Pagani, A. Stark, C. D. Arnold, STARR-seq and UMI-STARR-seq: Assessing enhancer activities for genome-wide-, high-, and low-complexity candidate libraries. Curr. Protoc. Mol. Biol. 128, e105 (2019). 10.1002/cpmb.105 PMC9286403 31503413

[40] F. Muerdter, Ł. M. Boryń, A. R. Woodfin, C. Neumayr, M. Rath, M. A. Zabidi, M. Pagani, V. Haberle, T. Kazmar, R. R. Catarino, K. Schernhuber, C. D. Arnold, A. Stark, Resolving systematic errors in widely used enhancer activity assays in human cells. Nat. Methods 15, 141–149 (2018). 10.1038/nmeth.4534 PMC5793997 29256496

[41] I. Streeter, P. W. Harrison, A. Faulconbridge, HipSci Consortium, P. Flicek, H. Parkinson, L. Clarke, The human-induced pluripotent stem cell initiative—Data resources for cellular genetics. Nucleic Acids Res. 45, D691–D697 (2017). 10.1093/nar/gkw928 PMC5210631 27733501

[42] F.-J. Müller, B. M. Schuldt, R. Williams, D. Mason, G. Altun, E. P. Papapetrou, S. Danner, J. E. Goldmann, A. Herbst, N. O. Schmidt, J. B. Aldenhoff, L. C. Laurent, J. F. Loring, A bioinformatic assay for pluripotency in human cells. Nat. Methods 8, 315–317 (2011). 10.1038/nmeth.1580 PMC3265323 21378979

[43] D. Lee, M. Shi, J. Moran, M. Wall, J. Zhang, J. Liu, D. Fitzgerald, Y. Kyono, L. Ma, K. P. White, M. Gerstein, STARRPeaker: Uniform processing and accurate identification of STARR-seq active regions. Genome Biol. 21, 298 (2020). 10.1186/s13059-020-02194-x PMC7722316 33292397

[44] M. R. Corces, A. E. Trevino, E. G. Hamilton, P. G. Greenside, N. A. Sinnott-Armstrong, S. Vesuna, A. T. Satpathy, A. J. Rubin, K. S. Montine, B. Wu, A. Kathiria, S. W. Cho, M. R. Mumbach, A. C. Carter, M. Kasowski, L. A. Orloff, V. I. Risca, A. Kundaje, P. A. Khavari, T. J. Montine, W. J. Greenleaf, H. Y. Chang, An improved ATAC-seq protocol reduces background and enables interrogation of frozen tissues. Nat. Methods 14, 959–962 (2017). 10.1038/nmeth.4396 PMC5623106 28846090

[45] J. Eggenberger, D. Blanco-Melo, M. Panis, K. J. Brennand, B. R. tenOever, Type I interferon response impairs differentiation potential of pluripotent stem cells. Proc. Natl. Acad. Sci. U.S.A. 116, 1384–1393 (2019). 10.1073/pnas.1812449116 PMC6347712 30606801

[46] Y. Liu, S. Yu, V. K. Dhiman, T. Brunetti, H. Eckart, K. P. White, Functional assessment of human enhancer activities using whole-genome STARR-sequencing. Genome Biol. 18, 219 (2017). 10.1186/s13059-017-1345-5 PMC5694901 29151363

[47] T. Peng, Y. Zhai, Y. Atlasi, M. ter Huurne, H. Marks, H. G. Stunnenberg, W. Megchelenbrink, STARR-seq identifies active, chromatin-masked, and dormant enhancers in pluripotent mouse embryonic stem cells. Genome Biol. 21, 243 (2020). 10.1186/s13059-020-02156-3 PMC7488044 32912294

[48] L. Hofbauer, L.-M. Pleyer, F. Reiter, A. Schleiffer, A. Vlasova, L. Serebreni, A. Huang, A. Stark, A genome-wide screen identifies silencers with distinct chromatin properties and mechanisms of repression. Mol. Cell 84, 4503–4521.e14 (2024). 10.1016/j.molcel.2024.10.041 39571581

[49] B. P. de Almeida, F. Reiter, M. Pagani, A. Stark, DeepSTARR predicts enhancer activity from DNA sequence and enables the de novo design of synthetic enhancers. Nat. Genet. 54, 613–624 (2022). 10.1038/s41588-022-01048-5 35551305

[50] I. Hossain, P. Priam, S. C. Reynoso, S. Sahni, X. X. Zhang, L. Côté, J. Doumat, C. Chik, T. Fu, J. A. Lessard, W. A. Pastor, ZIC2 and ZIC3 promote SWI/SNF recruitment to safeguard progression towards human primed pluripotency. Nat. Commun. 15, 8539 (2024). 10.1038/s41467-024-52431-1 PMC11447223 39358345

[51] S. Wingett, P. Ewels, M. Furlan-Magaril, T. Nagano, S. Schoenfelder, P. Fraser, S. Andrews, HiCUP: Pipeline for mapping and processing Hi-C data. F1000Res 4, 1310 (2015). 10.12688/f1000research.7334.1 PMC4706059 26835000

[52] J. Cairns, P. Freire-Pritchett, S. W. Wingett, C. Várnai, A. Dimond, V. Plagnol, D. Zerbino, S. Schoenfelder, B.-M. Javierre, C. Osborne, P. Fraser, M. Spivakov, CHiCAGO: Robust detection of DNA looping interactions in Capture Hi-C data. Genome Biol. 17, 127 (2016). 10.1186/s13059-016-0992-2 PMC4908757 27306882

[53] J. M. Alexander, J. Guan, B. Li, L. Maliskova, M. Song, Y. Shen, B. Huang, S. Lomvardas, O. D. Weiner, Live-cell imaging reveals enhancer-dependent Sox2 transcription in the absence of enhancer proximity. eLife 8, e41769 (2019). 10.7554/eLife.41769 PMC6534382 31124784

[54] N. S. Benabdallah, I. Williamson, R. S. Illingworth, L. Kane, S. Boyle, D. Sengupta, G. R. Grimes, P. Therizols, W. A. Bickmore, Decreased enhancer-promoter proximity accompanying enhancer activation. Mol. Cell 76, 473–484.e7 (2019). 10.1016/j.molcel.2019.07.038 PMC6838673 31494034

[55] S. Cruz-Molina, P. Respuela, C. Tebartz, P. Kolovos, M. Nikolic, R. Fueyo, W. F. J. van Ijcken, F. Grosveld, P. Frommolt, H. Bazzi, A. Rada-Iglesias, PRC2 facilitates the regulatory topology required for poised enhancer function during pluripotent stem cell differentiation. Cell Stem Cell 20, 689–705.e9 (2017). 10.1016/j.stem.2017.02.004 28285903

[56] W. Xie, M. D. Schultz, R. Lister, Z. Hou, N. Rajagopal, P. Ray, J. W. Whitaker, S. Tian, R. D. Hawkins, D. Leung, H. Yang, T. Wang, A. Y. Lee, S. A. Swanson, J. Zhang, Y. Zhu, A. Kim, J. R. Nery, M. A. Urich, S. Kuan, C. Yen, S. Klugman, P. Yu, K. Suknuntha, N. E. Propson, H. Chen, L. E. Edsall, U. Wagner, Y. Li, Z. Ye, A. Kulkarni, Z. Xuan, W.-Y. Chung, N. C. Chi, J. E. Antosiewicz-Bourget, I. Slukvin, R. Stewart, M. Q. Zhang, W. Wang, J. A. Thomson, J. R. Ecker, B. Ren, Epigenomic analysis of multilineage differentiation of human embryonic stem cells. Cell 153, 1134–1148 (2013). 10.1016/j.cell.2013.04.022 PMC3786220 23664764

[57] P. Chovanec, A. J. Collier, C. Krueger, C. Várnai, C. I. Semprich, S. Schoenfelder, A. E. Corcoran, P. J. Rugg-Gunn, Widespread reorganisation of pluripotent factor binding and gene regulatory interactions between human pluripotent states. Nat. Commun. 12, 2098 (2021). 10.1038/s41467-021-22201-4 PMC8026613 33828098

[58] L. A. Lettice, S. J. H. Heaney, L. A. Purdie, L. Li, P. de Beer, B. A. Oostra, D. Goode, G. Elgar, R. E. Hill, E. de Graaff, A long-range Shh enhancer regulates expression in the developing limb and fin and is associated with preaxial polydactyly. Hum. Mol. Genet. 12, 1725–1735 (2003). 10.1093/hmg/ddg180 12837695

[59] C. D. Todd, J. Ijaz, F. Torabi, O. Dovgusha, S. Bevan, O. Cracknell, T. Lohoff, S. Clark, R. Argelaguet, J. Pierce, I. Kafetzopoulos, A. Santambrogio, J. Nichols, F. von Meyenn, U. Günesdogan, S. Schoenfelder, W. Reik, Epigenetic priming of mammalian embryonic enhancer elements coordinates developmental gene networks. Genome Biol. 26, 214 (2025). 10.1186/s13059-025-03658-8 PMC12272991 40682180

[60] A. M. Miangolarra, J. Kurlovich, F. Torabi, O. Dovgusha, Y. Cao, L. Lin, U. Günesdogan, S. Schoenfelder, M. Howard, Signatures of digital Polycomb regulation in functional iPSC heterogeneity between individuals. bioRxiv 666753 [Preprint] (2026). 10.1101/2025.07.25.666753.

[61] Ž. Avsec, N. Latysheva, J. Cheng, G. Novati, K. R. Taylor, T. Ward, C. Bycroft, L. Nicolaisen, E. Arvaniti, J. Pan, R. Thomas, V. Dutordoir, M. Perino, S. De, A. Karollus, A. Gayoso, T. Sargeant, A. Mottram, L. H. Wong, P. Drotár, A. Kosiorek, A. Senior, R. Tanburn, T. Applebaum, S. Basu, D. Hassabis, P. Kohli, Advancing regulatory variant effect prediction with AlphaGenome. Nature 649, 1206–1218 (2026). 10.1038/s41586-025-10014-0 PMC12851941 41606153

[62] C. P. Fulco, M. Munschauer, R. Anyoha, G. Munson, S. R. Grossman, E. M. Perez, M. Kane, B. Cleary, E. S. Lander, J. M. Engreitz, Systematic mapping of functional enhancer-promoter connections with CRISPR interference. Science 354, 769–773 (2016). 10.1126/science.aag2445 PMC5438575 27708057

[63] M. Gasperini, A. J. Hill, J. L. McFaline-Figueroa, B. Martin, S. Kim, M. D. Zhang, D. Jackson, A. Leith, J. Schreiber, W. S. Noble, C. Trapnell, N. Ahituv, J. Shendure, A genome-wide framework for mapping gene regulation via cellular genetic screens. Cell 176, 377–390.e19 (2019). 10.1016/j.cell.2018.11.029 PMC6690346 30612741

[64] D. Schraivogel, A. R. Gschwind, J. H. Milbank, D. R. Leonce, P. Jakob, L. Mathur, J. O. Korbel, C. A. Merten, L. Velten, L. M. Steinmetz, Targeted Perturb-seq enables genome-scale genetic screens in single cells. Nat. Methods 17, 629–635 (2020). 10.1038/s41592-020-0837-5 PMC7610614 32483332

[65] T. Narita, Y. Higashijima, S. Kilic, T. Liebner, J. Walter, C. Choudhary, Acetylation of histone H2B marks active enhancers and predicts CBP/p300 target genes. Nat. Genet. 55, 679–692 (2023). 10.1038/s41588-023-01348-4 PMC10101849 37024579

[66] M. M. Pradeepa, Causal role of histone acetylations in enhancer function. Transcription 8, 40–47 (2017). 10.1080/21541264.2016.1253529 PMC5279748 27792455

[67] G. E. Zentner, P. J. Tesar, P. C. Scacheri, Epigenetic signatures distinguish multiple classes of enhancers with distinct cellular functions. Genome Res. 21, 1273–1283 (2011). 10.1101/gr.122382.111 PMC3149494 21632746

[68] D. A. Braun, C. E. Sadowski, S. Kohl, S. Lovric, S. A. Astrinidis, W. L. Pabst, H. Y. Gee, S. Ashraf, J. A. Lawson, S. Shril, M. Airik, W. Tan, D. Schapiro, J. Rao, W.-I. Choi, T. Hermle, M. J. Kemper, M. Pohl, F. Ozaltin, M. Konrad, R. Bogdanovic, R. Büscher, U. Helmchen, E. Serdaroglu, R. P. Lifton, W. Antonin, F. Hildebrandt, Mutations in nuclear pore genes NUP93, NUP205 and XPO5 cause steroid-resistant nephrotic syndrome. Nat. Genet. 48, 457–465 (2016). 10.1038/ng.3512 PMC4811732 26878725

[69] W. Chen, Y. Zhang, S. Yang, Z. Shi, W. Zeng, Z. Lu, X. Zhou, Bi-allelic mutations in NUP205 and NUP210 are associated with abnormal cardiac left-right patterning. Circ. Genom. Precis. Med. 12, e002492 (2019). 10.1161/CIRCGEN.119.002492 31306055

[70] P. A. Dawson, J. Rakoczy, D. G. Simmons, Placental, renal, and ileal sulfate transporter gene expression in mouse gestation. Biol. Reprod. 87, 43 (2012). 10.1095/biolreprod.111.098749 22674389

[71] D. G. Simmons, J. Rakoczy, J. Jefferis, R. Lourie, H. D. McIntyre, P. A. Dawson, Human placental sulfate transporter mRNA profiling from term pregnancies identifies abundant *SLC13A4* in syncytiotrophoblasts and *SLC26A2* in cytotrophoblasts. Placenta 34, 381–384 (2013). 10.1016/j.placenta.2013.01.017 23453247

[72] T. J. Harvey, R. A. Davila, D. Vidovic, S. Sharmin, M. Piper, D. G. Simmons, Genome-wide transcriptomic analysis of the forebrain of postnatal *Slc13a4*^+/−^ mice. BMC. Res. Notes 14, 269 (2021). 10.1186/s13104-021-05687-5 PMC8276513 34256843

[73] A. Yilmaz, M. Peretz, A. Aharony, I. Sagi, N. Benvenisty, Defining essential genes for human pluripotent stem cells by CRISPR-Cas9 screening in haploid cells. Nat. Cell Biol. 20, 610–619 (2018). 10.1038/s41556-018-0088-1 29662178

[74] A. Eberharter, P. B. Becker, Histone acetylation: A switch between repressive and permissive chromatin. EMBO Rep. 3, 224–229 (2002). 10.1093/embo-reports/kvf053 PMC1084017 11882541

[75] S. J. Hogg, O. Motorna, L. A. Cluse, T. M. Johanson, H. D. Coughlan, R. Raviram, R. M. Myers, M. Costacurta, I. Todorovski, L. Pijpers, S. Bjelosevic, T. Williams, S. N. Huskins, C. J. Kearney, J. R. Devlin, Z. Fan, J. S. Jabbari, B. P. Martin, M. Fareh, M. J. Kelly, D. Dupéré-Richer, J. J. Sandow, B. Feran, D. Knight, T. Khong, A. Spencer, S. J. Harrison, G. Gregory, V. O. Wickramasinghe, A. I. Webb, P. C. Taberlay, K. D. Bromberg, A. Lai, A. T. Papenfuss, G. K. Smyth, R. S. Allan, J. D. Licht, D. A. Landau, O. Abdel-Wahab, J. Shortt, S. J. Vervoort, R. W. Johnstone, Targeting histone acetylation dynamics and oncogenic transcription by catalytic P300/CBP inhibition. Mol. Cell 81, 2183–2200.e13 (2021). 10.1016/j.molcel.2021.04.015 PMC8183601 34019788

[76] A. R. Pengelly, Ö. Copur, H. Jäckle, A. Herzig, J. Müller, A histone mutant reproduces the phenotype caused by loss of histone-modifying factor Polycomb. Science 339, 698–699 (2013). 10.1126/science.1231382 23393264

[77] J. E. Phillips-Cremins, V. G. Corces, Chromatin insulators: Linking genome organization to cellular function. Mol. Cell 50, 461–474 (2013). 10.1016/j.molcel.2013.04.018 PMC3670141 23706817

[78] N. Kubo, H. Ishii, X. Xiong, S. Bianco, F. Meitinger, R. Hu, J. D. Hocker, M. Conte, D. Gorkin, M. Yu, B. Li, J. R. Dixon, M. Hu, M. Nicodemi, H. Zhao, B. Ren, Promoter-proximal CTCF binding promotes distal enhancer-dependent gene activation. Nat. Struct. Mol. Biol. 28, 152–161 (2021). 10.1038/s41594-020-00539-5 PMC7913465 33398174

[79] N. Q. Liu, M. Magnitov, M. M. G. A. Schijns, T. van Schaik, H. Teunissen, B. van Steensel, E. de Wit, Extrusion fountains are restricted by WAPL-dependent cohesin release and CTCF barriers. Nucleic Acids Res. 53, gkaf549 (2025). 10.1093/nar/gkaf549 PMC12207409 40586309

[80] N. J. Rinzema, K. Sofiadis, S. J. D. Tjalsma, M. J. A. M. Verstegen, Y. Oz, C. Valdes-Quezada, A.-K. Felder, T. Filipovska, S. van der Elst, Z. de Andrade dos Ramos, R. Han, P. H. L. Krijger, W. de Laat, Building regulatory landscapes reveals that an enhancer can recruit cohesin to create contact domains, engage CTCF sites and activate distant genes. Nat. Struct. Mol. Biol. 29, 563–574 (2022). 10.1038/s41594-022-00787-7 PMC9205769 35710842

[81] T. Narita, S. Kilic, Y. Higashijima, N. M. Scherer, G. Pappas, E. Maskey, C. Choudhary, Disentangling the architectural and non-architectural functions of CTCF and cohesin in gene regulation. Nat. Genet. 57, 3137–3151 (2025). 10.1038/s41588-025-02404-x PMC12695661 41254162

[82] L. Braccioli, E. de Wit, CTCF: A Swiss-army knife for genome organization and transcription regulation. Essays Biochem. 63, 157–165 (2019). 10.1042/EBC20180069 30940740

[83] J. Dubois-Chevalier, F. Oger, H. Dehondt, F. F. Firmin, C. Gheeraert, B. Staels, P. Lefebvre, J. Eeckhoute, A dynamic CTCF chromatin binding landscape promotes DNA hydroxymethylation and transcriptional induction of adipocyte differentiation. Nucleic Acids Res. 42, 10943–10959 (2014). 10.1093/nar/gku780 PMC4176165 25183525

[84] G. Ren, W. Jin, K. Cui, J. Rodrigez, G. Hu, Z. Zhang, D. R. Larson, K. Zhao, CTCF-mediated enhancer-promoter interaction is a critical regulator of cell-to-cell variation of gene expression. Mol. Cell 67, 1049–1058.e6 (2017). 10.1016/j.molcel.2017.08.026 PMC5828172 28938092

[85] J. Ray, E. Jagoda, M. U. Sheth, J. Galante, D. Amgalan, A. R. Gschwind, C. J. Munger, J. Huang, G. Munson, M. Murphy, E. Mattei, T. Barry, V. Singh, A. Baskaran, H. Kang, E. Katsevich, L. M. Steinmetz, J. M. Engreitz, A systematic survey of distal element-gene regulatory interactions with Direct-Capture Targeted Perturb-seq. bioRxiv 676677 [Preprint] (2025). 10.1101/2025.09.16.676677.

[86] O. Mikhaylichenko, V. Bondarenko, D. Harnett, I. E. Schor, M. Males, R. R. Viales, E. E. M. Furlong, The degree of enhancer or promoter activity is reflected by the levels and directionality of eRNA transcription. Genes Dev. 32, 42–57 (2018). 10.1101/gad.308619.117 PMC5828394 29378788

[87] F. Inoue, M. Kircher, B. Martin, G. M. Cooper, D. M. Witten, M. T. McManus, N. Ahituv, J. Shendure, A systematic comparison reveals substantial differences in chromosomal versus episomal encoding of enhancer activity. Genome Res. 27, 38–52 (2017). 10.1101/gr.212092.116 PMC5204343 27831498

[88] M. A. Zabidi, C. D. Arnold, K. Schernhuber, M. Pagani, M. Rath, O. Frank, A. Stark, Enhancer-core-promoter specificity separates developmental and housekeeping gene regulation. Nature 518, 556–559 (2015). 10.1038/nature13994 PMC6795551 25517091

[89] B. Sahu, T. Hartonen, P. Pihlajamaa, B. Wei, K. Dave, F. Zhu, E. Kaasinen, K. Lidschreiber, M. Lidschreiber, C. O. Daub, P. Cramer, T. Kivioja, J. Taipale, Sequence determinants of human gene regulatory elements. Nat. Genet. 54, 283–294 (2022). 10.1038/s41588-021-01009-4 PMC8920891 35190730

[90] R. M. Boileau, K. X. Chen, R. Blelloch, Loss of MLL3/4 decouples enhancer H3K4 monomethylation, H3K27 acetylation, and gene activation during embryonic stem cell differentiation. Genome Biol. 24, 41 (2023). 10.1186/s13059-023-02883-3 PMC9983171 36869380

[91] I. Olan, M. Ando-Kuri, A. J. Parry, T. Handa, S. Schoenfelder, P. Fraser, Y. Ohkawa, H. Kimura, M. Narita, M. Narita, HMGA1 orchestrates chromatin compartmentalization and sequesters genes into 3D networks coordinating senescence heterogeneity. Nat. Commun. 15, 6891 (2024). 10.1038/s41467-024-51153-8 PMC11319441 39134516

[92] H. Vlaming, C. A. Mimoso, A. R. Field, B. J. E. Martin, K. Adelman, Screening thousands of transcribed coding and non-coding regions reveals sequence determinants of RNA polymerase II elongation potential. Nat. Struct. Mol. Biol. 29, 613–620 (2022). 10.1038/s41594-022-00785-9 PMC9672417 35681023

[93] A. D. Bell, C. L. Usher, S. A. McCarroll, Analyzing copy number variation with droplet digital PCR. Methods Mol. Biol. 1768, 143–160 (2018). 10.1007/978-1-4939-7778-9_9 29717442

[94] A. Dobin, C. A. Davis, F. Schlesinger, J. Drenkow, C. Zaleski, S. Jha, P. Batut, M. Chaisson, T. R. Gingeras, STAR: Ultrafast universal RNA-seq aligner. Bioinformatics 29, 15–21 (2013). 10.1093/bioinformatics/bts635 PMC3530905 23104886

[95] S. Anders, P. T. Pyl, W. Huber, HTSeq—A Python framework to work with high-throughput sequencing data. Bioinformatics 31, 166–169 (2015). 10.1093/bioinformatics/btu638 PMC4287950 25260700

[96] M. I. Love, W. Huber, S. Anders, Moderated estimation of fold change and dispersion for RNA-seq data with DESeq2. Genome Biol. 15, 550 (2014). 10.1186/s13059-014-0550-8 PMC4302049 25516281

[97] H. Wickham, *ggplot2: Elegant Graphics for Data Analysis* (Springer, Switzerland, ed. 1, 2009).

[98] G. Yu, L.-G. Wang, Y. Han, Q.-Y. He, clusterProfiler: An R package for comparing biological themes among gene clusters. OMICS 16, 284–287 (2012). 10.1089/omi.2011.0118 PMC3339379 22455463

[99] F. Ramírez, D. P. Ryan, B. Grüning, V. Bhardwaj, F. Kilpert, A. S. Richter, S. Heyne, F. Dündar, T. Manke, deepTools2: A next generation web server for deep-sequencing data analysis. Nucleic Acids Res. 44, W160–W165 (2016). 10.1093/nar/gkw257 PMC4987876 27079975

[100] M. Martin, Cutadapt removes adapter sequences from high-throughput sequencing reads. EMBnet J. 17, 10–12 (2011).

[101] B. Langmead, S. L. Salzberg, Fast gapped-read alignment with Bowtie 2. Nat. Methods 9, 357–359 (2012). 10.1038/nmeth.1923 PMC3322381 22388286

[102] H. Li, B. Handsaker, A. Wysoker, T. Fennell, J. Ruan, N. Homer, G. Marth, G. Abecasis, R. Durbin, 1000 Genome Project Data Processing Subgroup, The Sequence Alignment/Map format and SAMtools. Bioinformatics 25, 2078–2079 (2009). 10.1093/bioinformatics/btp352 PMC2723002 19505943

[103] H. M. Amemiya, A. Kundaje, A. P. Boyle, The ENCODE Blacklist: Identification of problematic regions of the genome. Sci. Rep. 9, 9354 (2019). 10.1038/s41598-019-45839-z PMC6597582 31249361

[104] J. Feng, T. Liu, B. Qin, Y. Zhang, X. S. Liu, Identifying ChIP-seq enrichment using MACS. Nat. Protoc. 7, 1728–1740 (2012). 10.1038/nprot.2012.101 PMC3868217 22936215

[105] R. C. McLeay, T. L. Bailey, Motif Enrichment Analysis: A unified framework and an evaluation on ChIP data. BMC Bioinformatics 11, 165 (2010). 10.1186/1471-2105-11-165 PMC2868005 20356413

[106] T. L. Bailey, J. Johnson, C. E. Grant, W. S. Noble, The MEME suite. Nucleic Acids Res. 43, W39–W49 (2015). 10.1093/nar/gkv416 PMC4489269 25953851

