# Supplementary material for: Noncanonical enhancers control gene expression and cell fate in human pluripotent stem cells
Source: Sci Adv. 2026 Nov 13;12(46):eadz3328. doi: 10.1126/sciadv.adz3328 (PMC13763835; doi:10.1126/sciadv.adz3328)
Supplement: Supplementary file 1 [file sciadv.adz3328_sm.pdf]

Science Advances
AAAS

Supplementary Materials for

Noncanonical enhancers control gene expression and cell fate in human pluripotent stem cells

Yang Cao *et al.*

Corresponding author: Ufuk Günesdogan, ufuk.guenesdogan@biologie.uni-goettingen.de;
Stefan Schoenfelder, stefan.schoenfelder@babraham.ac.uk

Sci. Adv. **12**, eadz3328 (2026)
DOI: 10.1126/sciadv.adz3328

The PDF file includes:

Figs. S1 to S6
Legends for tables S1 to S9
Legends for movies S1 to S6
References

Other Supplementary Material for this manuscript includes the following:

Tables S1 to S9
Movies S1 to S6

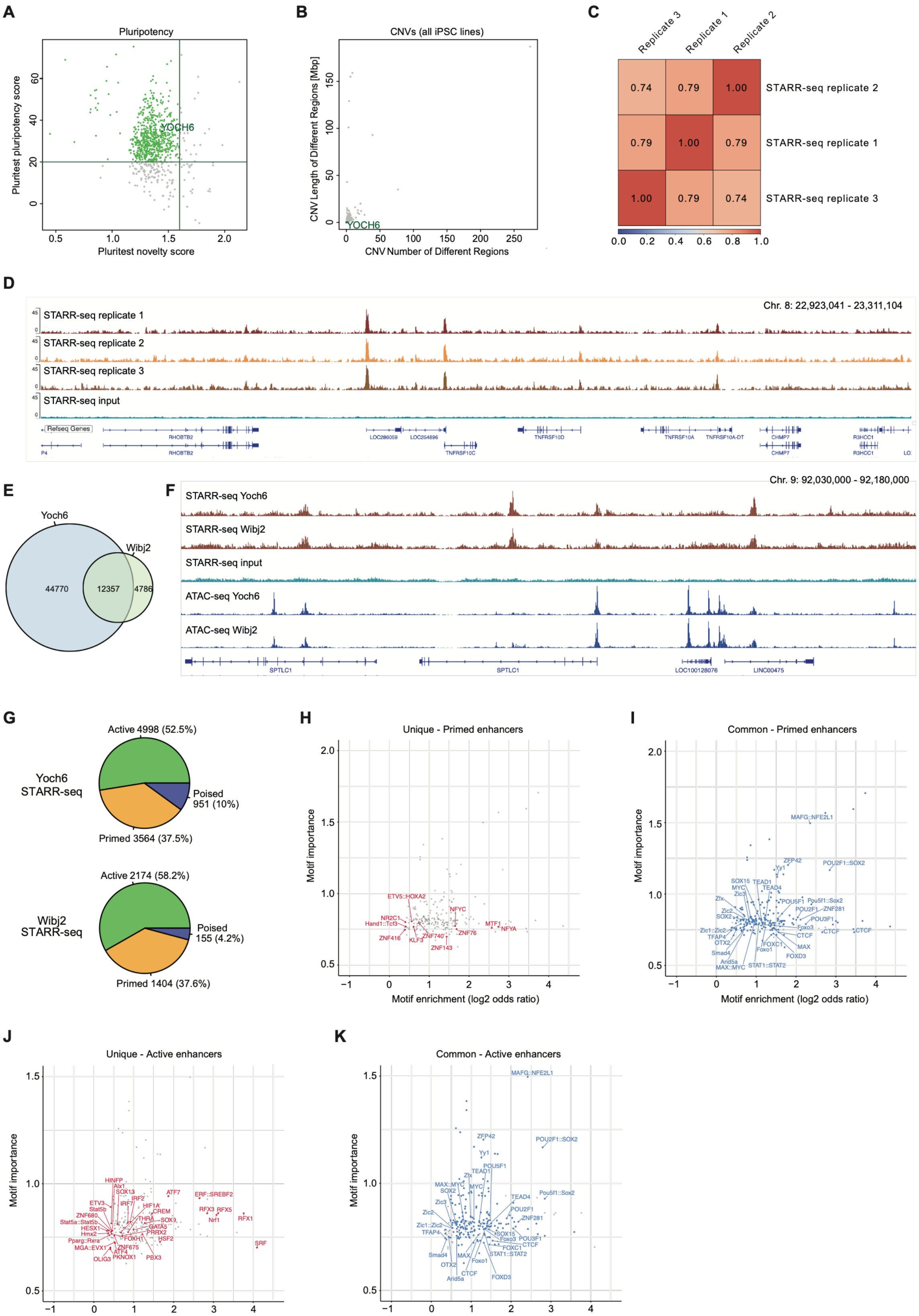
A
Pluripotency
Pluritest pluripotency score
Pluritest novelty score
YOCH6
B
CNVs (all iPSC lines)
CNV Length of Different Regions [Mbp]
CNV Number of Different Regions
YOCH6
C
Replicate 3
Replicate 1
Replicate 2
0.74
0.79
1.00
STARR-seq replicate 2
0.79
1.00
0.79
STARR-seq replicate 1
1.00
0.79
0.74
STARR-seq replicate 3
D
Chr. 8: 22,923,041 - 23,311,104
STARR-seq replicate 1
STARR-seq replicate 2
STARR-seq replicate 3
STARR-seq input
Refseq Genes
RHOBTB2
TNFRSF10C
TNFRSF10D
TNFRSF10A
TNFRSF10A-DT
CHMP7
R3HCC1
E
Yoch6
Wibj2
44770
12357
4786
F
Chr. 9: 92,030,000 - 92,180,000
STARR-seq Yoch6
STARR-seq Wibj2
STARR-seq input
ATAC-seq Yoch6
ATAC-seq Wibj2
SPTLC1
LOC100128076
LINC00475
G
Yoch6
STARR-seq
Active 4998 (52.5%)
Poised
951 (10%)
Primed 3564 (37.5%)
Wibj2
STARR-seq
Active 2174 (58.2%)
Poised
155 (4.2%)
Primed 1404 (37.6%)
H
Unique - Primed enhancers
Motif importance
Motif enrichment (log2 odds ratio)
I
Common - Primed enhancers
Motif importance
Motif enrichment (log2 odds ratio)
J
Unique - Active enhancers
Motif importance
Motif enrichment (log2 odds ratio)
K
Common - Active enhancers
Motif importance
Motif enrichment (log2 odds ratio)

Figure S1. STARR-seq quality control and sequence motifs in active and primed enhancers.
(**A**) Scatter plot showing PluriTest scores of HipSci hiPSC lines for pluripotency and novelty. The hiPSC line used for STARR-seq is highlighted (Yoch6; HPSI0215i-yoch_6).
(**B**) Copy Number Variations (CNVs) in HipSci hiPSC lines. HiPSC line Yoch6 is highlighted.
(**C**) Pearson correlations of STARR-seq replicates.
(**D**) Example coverage tracks of STARR-seq replicates 1-3 and the STARR-seq input background control.
(**E**) Venn diagram showing the overlap between Yoch6 and Wibj2 STARR-seq peaks.
(**F**) STARR-seq and ATAC-seq IGV screenshots of human chromosome 9:92,030,000-92,180,000 for HPSI0215i-yoch_6 (Yoch6) and HPSI0214i-wibj_2 (Wibj2).
(**G**) Numbers and proportions of active, primed and poised STARR-seq enhancers in the Yoch6 and Wibj2 hiPSC lines.
(**H**-**I**) Scatter plot showing motif enrichment (x-axis) and DeepSTARR-predicted global importance (y-axis) at primed enhancers (red). Unique (H): Motifs called only at primed enhancers in comparison to active enhancers. Common (I): Motifs called at both enhancer classes.
(**J**-**K**) Scatter plot showing motif enrichment (x-axis) and DeepSTARR-predicted global importance (y-axis) at active enhancers (red). Unique (J): Motifs called only at active enhancers in comparison to primed enhancers. Common (K): Motifs called at both enhancer classes.

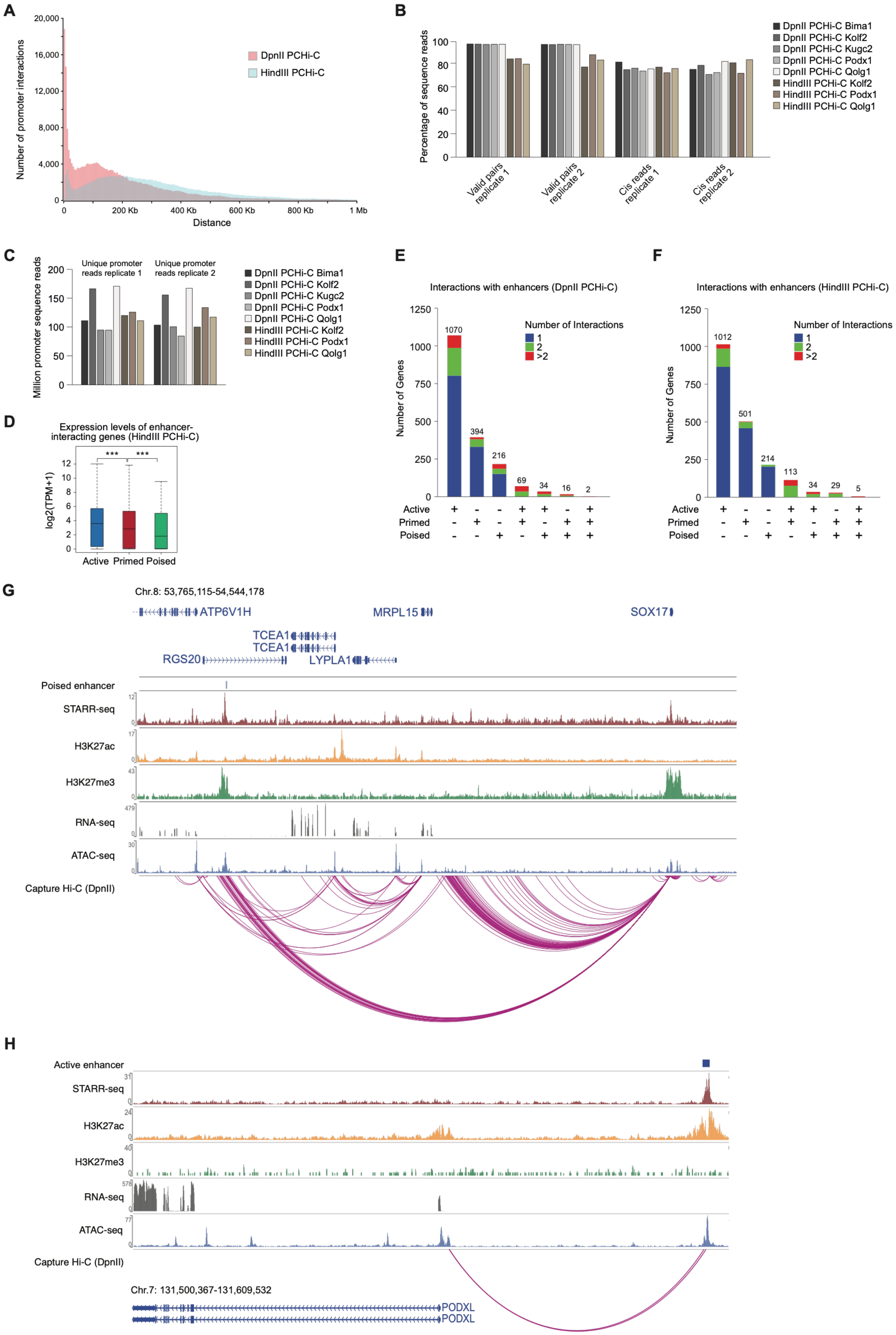

A
20,000
16,000
12,000
8,000
4,000
0
Number of promoter interactions
DpnII PCHi-C
HindIII PCHi-C
0
200 Kb
400 Kb
600 Kb
800 Kb
1 Mb
Distance
B
Percentage of sequence reads
100
80
60
40
20
0
Valid pairs replicate 1
Valid pairs replicate 2
Cis reads replicate 1
Cis reads replicate 2
DpnII PCHi-C Bima1
DpnII PCHi-C Kolf2
DpnII PCHi-C Kugc2
DpnII PCHi-C Podx1
DpnII PCHi-C Qolg1
HindIII PCHi-C Kolf2
HindIII PCHi-C Podx1
HindIII PCHi-C Qolg1
C
Million promoter sequence reads
200
150
100
50
0
Unique promoter reads replicate 1
Unique promoter reads replicate 2
DpnII PCHi-C Bima1
DpnII PCHi-C Kolf2
DpnII PCHi-C Kugc2
DpnII PCHi-C Podx1
DpnII PCHi-C Qolg1
HindIII PCHi-C Kolf2
HindIII PCHi-C Podx1
HindIII PCHi-C Qolg1
D
Expression levels of enhancer-interacting genes (HindIII PCHi-C)
log2(TPM+1)
12
10
8
6
4
2
0
***
***
Active
Primed
Poised
E
Interactions with enhancers (DpnII PCHi-C)
Number of Genes
1250
1000
750
500
250
0
Number of Interactions
1
2
>2
1070
394
216
69
34
16
2
Active + - - + + - +
Primed - + - + - + +
Poised - - + - + + +
F
Interactions with enhancers (HindIII PCHi-C)
Number of Genes
1250
1000
750
500
250
0
Number of Interactions
1
2
>2
1012
501
214
113
34
29
5
Active + - - + + - +
Primed - + - + - + +
Poised - - + - + + +
G
Chr.8: 53,765,115-54,544,178
ATP6V1H
MRPL15
SOX17
TCEA1
TCEA1
RGS20
LYPLA1
Poised enhancer
STARR-seq
12
0
H3K27ac
17
0
H3K27me3
43
0
RNA-seq
479
0
ATAC-seq
30
0
Capture Hi-C (DpnII)
H
Active enhancer
STARR-seq
31
0
H3K27ac
24
0
H3K27me3
40
0
RNA-seq
578
0
ATAC-seq
77
0
Capture Hi-C (DpnII)
Chr.7: 131,500,367-131,609,532
PODXL
PODXL

Figure S2. Promoter Capture Hi-C identifies target genes of enhancers.

(**A**) Number of interactions and their distances to captured promoters in bp in PCHi-C data generated using restriction enzymes with a six bp recognition sequence (HindIII PCHi-C) or a four bp recognition sequence (DpnII PCHi-C) in hiPSCs.

(**B**) Percentage of valid and *cis* sequencing reads in HindIII or DpnII PCHi-C datasets with indicated hiPSC cell lines.

(**C**) Number of promoter-centered reads (Unique promoter reads) in indicated PCHi-C datasets.

(**D**) Expression (RNA-seq, log2 (normalized counts+1)) of enhancer-interacting genes. Interactions were mapped with HindIII PCHi-C data in hiPSCs.

(**E**) Number of genes interacting with one, two or more than two active, primed and/or poised enhancers. Interactions were mapped using DpnII PCHi-C data.

(**F**) Number of genes interacting with one, two or more than two active, primed and/or poised enhancers. Interactions were mapped using HindIII PCHi-C data.

(**G**) Coverage tracks of genome-wide data on chromosome 8 showing a representative example of a poised enhancer. Capture Hi-C corresponds to DpnII PCHi-C data.

(**H**) Coverage tracks of genome-wide data on chromosome 7 showing a representative example of an active enhancer. Capture Hi-C corresponds to DpnII PCHi-C data.

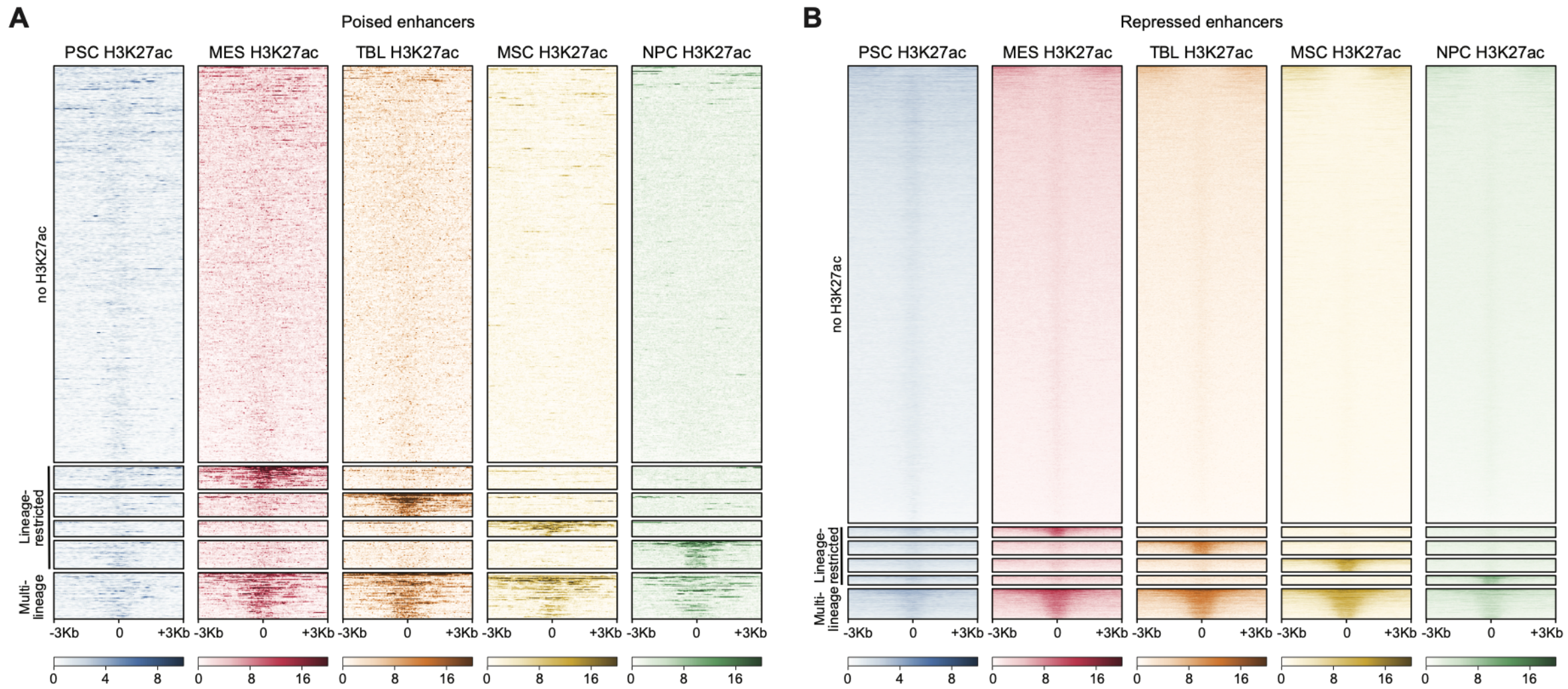

Figure S3. The majority of poised and chromatin-repressed enhancers in hiPSCs do not gain H3K27ac during differentiation.

(**A**) Heatmap showing H3K27ac enrichment (ChIP-seq) at poised enhancers (mapped in hiPSCs) +/- 3kb of STARR-seq peak centers in primed human pluripotent stem cells (PSC), mesendoderm (MES), trophoblast-like cells (TBL), mesenchymal stem cells (MSC), and neuronal progenitor cells (NPC). The majority of enhancers are not enriched for H3K27ac in any of the analyzed cell types (no H3K27ac). Subsets are enriched for H3K27ac in one (Lineage-restricted) or multiple differentiated cell types (Multi-lineage).
(**B**) Heatmap showing H3K27ac enrichment (ChIP-seq**)** at chromatin-repressed enhancers (mapped in hiPSCs) +/- 3kb of STARR-seq peak centers in PSC, MES, TBL, MSC, and NPC.

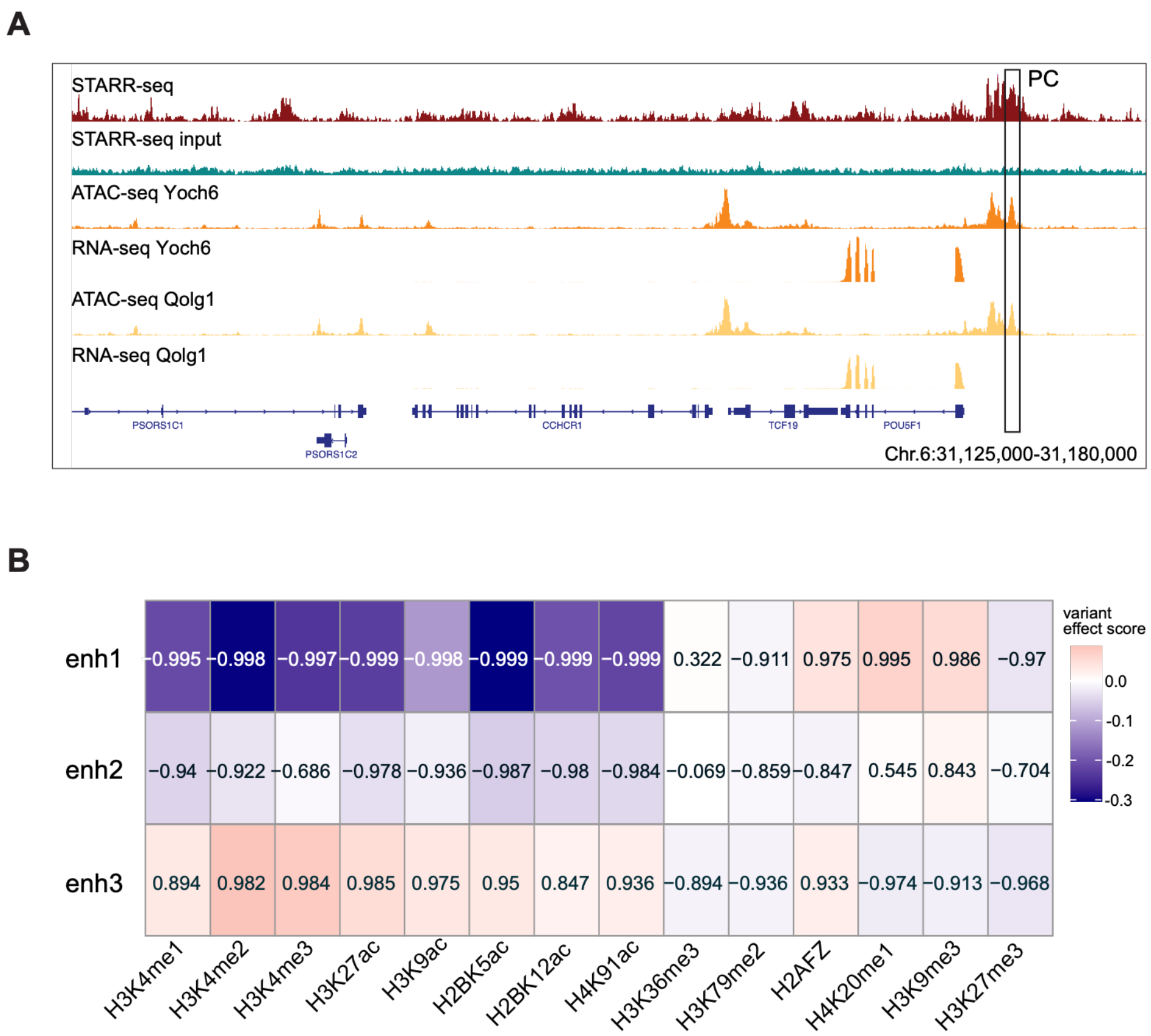

Figure S4. AlphaGenome predicts changes in histone modifications at enhancer variants.
(**A**) Coverage tracks of STARR-seq (Yoch6) as well as ATAC-seq and RNA-seq data from two independent hiPSC lines (Yoch6 and Qolg1) for the positive control region (*POU5F1* distal enhancer) used in the luciferase assay in Fig. 4C.
(**B**) AlphaGenome predictions for the effect of variants at enhancers on the enrichment of active and repressive histone modifications. Enhancer variants are shown in Fig. 4C-F, and the Yoch6 enhancers (enh1-3) were used as a reference.

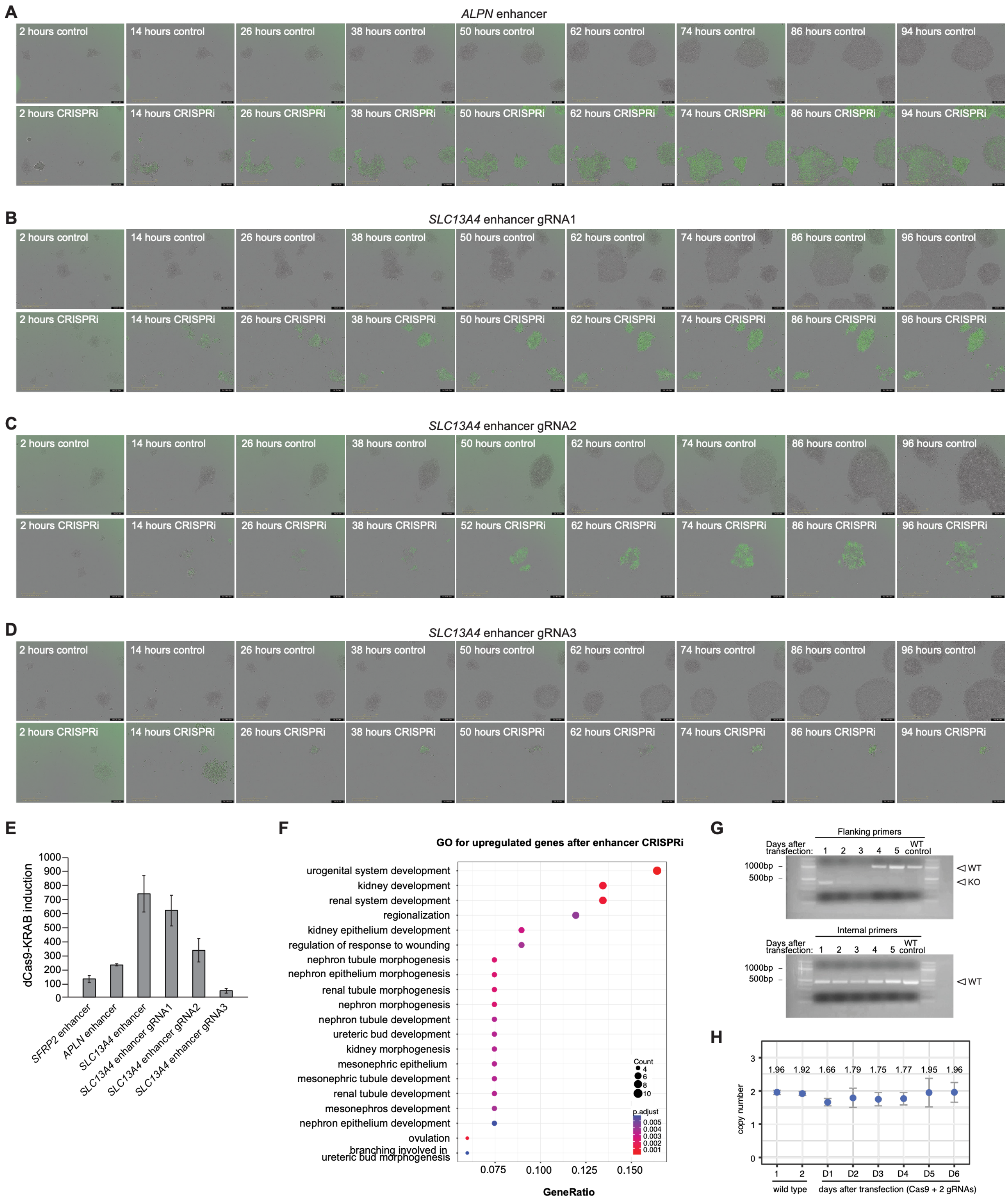
A
ALPN enhancer
2 hours control
14 hours control
26 hours control
38 hours control
50 hours control
62 hours control
74 hours control
86 hours control
94 hours control
2 hours CRISPRi
14 hours CRISPRi
26 hours CRISPRi
38 hours CRISPRi
50 hours CRISPRi
62 hours CRISPRi
74 hours CRISPRi
86 hours CRISPRi
94 hours CRISPRi
B
SLC13A4 enhancer gRNA1
2 hours control
14 hours control
26 hours control
38 hours control
50 hours control
62 hours control
74 hours control
86 hours control
96 hours control
2 hours CRISPRi
14 hours CRISPRi
26 hours CRISPRi
38 hours CRISPRi
50 hours CRISPRi
62 hours CRISPRi
74 hours CRISPRi
86 hours CRISPRi
96 hours CRISPRi
C
SLC13A4 enhancer gRNA2
2 hours control
14 hours control
26 hours control
38 hours control
50 hours control
62 hours control
74 hours control
86 hours control
96 hours control
2 hours CRISPRi
14 hours CRISPRi
26 hours CRISPRi
38 hours CRISPRi
52 hours CRISPRi
62 hours CRISPRi
74 hours CRISPRi
86 hours CRISPRi
96 hours CRISPRi
D
SLC13A4 enhancer gRNA3
2 hours control
14 hours control
26 hours control
38 hours control
50 hours control
62 hours control
74 hours control
86 hours control
96 hours control
2 hours CRISPRi
14 hours CRISPRi
26 hours CRISPRi
38 hours CRISPRi
50 hours CRISPRi
62 hours CRISPRi
74 hours CRISPRi
86 hours CRISPRi
94 hours CRISPRi
E
dCas9-KRAB induction
1000
900
800
700
600
500
400
300
200
100
0
SFRP2 enhancer
APLN enhancer
SLC13A4 enhancer
SLC13A4 enhancer gRNA1
SLC13A4 enhancer gRNA2
SLC13A4 enhancer gRNA3
F
GO for upregulated genes after enhancer CRISPRi
urogenital system development
kidney development
renal system development
regionalization
kidney epithelium development
regulation of response to wounding
nephron tubule morphogenesis
nephron epithelium morphogenesis
renal tubule morphogenesis
nephron morphogenesis
nephron tubule development
ureteric bud development
kidney morphogenesis
mesonephric epithelium
mesonephric tubule development
renal tubule development
mesonephros development
nephron epithelium development
ovulation
branching involved in ureteric bud morphogenesis
Count
4
6
8
10
p.adjust
0.005
0.004
0.003
0.002
0.001
0.075
0.100
0.125
0.150
GeneRatio
G
Flanking primers
Days after transfection:
1
2
3
4
5
WT control
1000bp
500bp
WT
KO
Internal primers
Days after transfection:
1
2
3
4
5
WT control
1000bp
500bp
WT
H
copy number
3
2
1
0
1.96
1.92
1.66
1.79
1.75
1.77
1.95
1.96
1
2
D1
D2
D3
D4
D5
D6
wild type
days after transfection (Cas9 + 2 gRNAs)

Figure S5. Repression of the *SLC13A4* enhancer impairs cell viability.

(**A**) Live-cell imaging of hiPSCs without (control) or with (CRISPRi) the addition of DOX and TMP to induce the expression of dCas9-KRAB, marked by GFP expression (green). Constitutive expression of three gRNAs targeting the primed enhancer of *APLN*.

(**B**-**D**) Live-cell imaging of hiPSCs without (control) or with (CRISPRi) the addition of DOX and TMP to induce the expression of dCas9-KRAB, marked by GFP expression (green). Constitutive expression of individual gRNAs (gRNA 1-3) targeting the primed enhancer of *SLC13A4*.

(**E**) Quantitative RT-PCR for dCas9-KRAB after the addition of DOX and TMP (for 4 days) in hiPSC lines with constitutive expression of gRNAs targeting enhancers of *SFRP2*, *APLN*, or *SLC13A4*. $2^{-\Delta\Delta Ct}$ ± s.d.; normalization using GAPDH (housekeeping gene) and uninduced hiPSCs as a reference (= 1).

(**F**) GO term enrichment of differentially upregulated genes in RNA-seq datasets from hiPSCs after the addition of DOX and TMP for 1 day to induce dCas9-KRAB with constitutive expression of gRNAs targeting the enhancer of *SLC13A4* in comparison to control hiPSCs.

(**G**) Gel electrophoresis analysis of PCR-amplified genomic DNA from untransfected hiPSCs (WT) and hiPSCs transfected with gRNAs to delete the *SLC13A4* enhancer (KO). Primers flanking the *SLC13A4* enhancer (top) or internal control primers (bottom) were used. The analysis confirmed the enhancer deletion from the endogenous genomic locus at early (days 1-3) but not later (days 4 and 5) time points after transfection.

(**H**) Digital droplet PCR analysis of *SLC13A4* enhancer deletion at indicated time points.

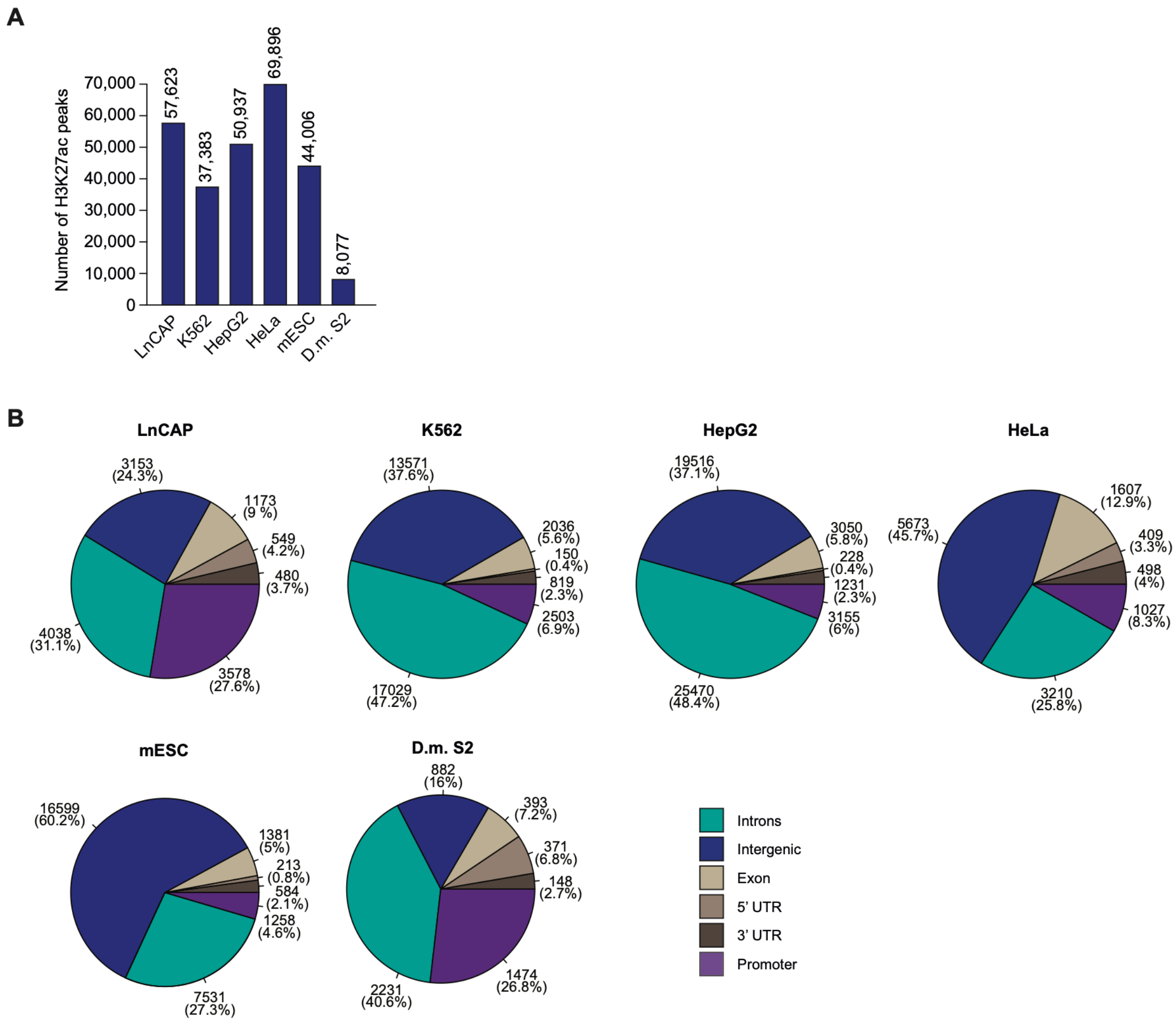

Figure S6. Genomic features of putative non-canonical enhancers.
(**A**) Number of called H3K27ac peaks (ChIP-seq) for datasets described in Figure 6D.
(**B**) Numbers (and percentage of total) of STARR-seq enhancers within indicated genomic features for datasets described in Figure 6D.

Table S1.
Genomic coordinates of chromatin-repressed hiPSC STARR-seq enhancers

Table S2.
Genomic coordinates of active hiPSC STARR-seq enhancers

Table S3.
Genomic coordinates of primed hiPSC STARR-seq enhancers

Table S4.
Genomic coordinates of poised hiPSC STARR-seq enhancers

Table S5.
DpnII Promoter Capture Hi-C probes

Table S6.
HindIII Promoter Capture Hi-C probes

Table S7.
RT-qPCR and next generation sequencing library generation primers

Table S8.
gRNAs for CRISPRi and CRISPRa

Table S9.
gRNAs and genotyping primers for CRISPR enhancer deletions

Movie S1.
SLC13A4 enhancer CRISPRi 3 gRNAs induced (hiPSC line Yoch6)

Movie S2.
SLC13A4 enhancer CRISPRi 3 gRNAs control (hiPSC line Yoch6)

Movie S3.
APLN enhancer CRISPRi 3 gRNAs control (hiPSC line Yoch6)

Movie S4.
APLN enhancer CRISPRi 3 gRNAs induced (hiPSC line Yoch6)

Movie S5.
SLC13A4 enhancer CRISPRi 3 gRNAs control (hiPSC line Kolf2)

Movie S6.
SLC13A4 enhancer CRISPRi 3 gRNAs induced (hiPSC line Kolf2)

REFERENCES

1. D. Shlyueva, G. Stampfel, A. Stark, Transcriptional enhancers: From properties to genome-wide predictions. *Nat. Rev. Genet.* **15**, 272–286 (2014).

2. S. Kim, J. Wysocka, Deciphering the multi-scale, quantitative cis-regulatory code. *Mol. Cell* **83**, 373–392 (2023).

3. E. E. M. Furlong, M. Levine, Developmental enhancers and chromosome topology. *Science* **361**, 1341–1345 (2018).

4. S. Schoenfelder, P. Fraser, Long-range enhancer–promoter contacts in gene expression control. *Nat. Rev. Genet.* **20**, 437–455 (2019).

5. ENCODE Project Consortium, An integrated encyclopedia of DNA elements in the human genome. *Nature* **489**, 57–74 (2012).

6. T. Sagai, M. Hosoya, Y. Mizushina, M. Tamura, T. Shiroishi, Elimination of a long-range cis-regulatory module causes complete loss of limb-specific Shh expression and truncation of the mouse limb. *Development* **132**, 797–803 (2005).

7. D. G. Lupiáñez, K. Kraft, V. Heinrich, P. Krawitz, F. Brancati, E. Klopocki, D. Horn, H. Kayserili, J. M. Opitz, R. Laxova, F. Santos-Simarro, B. Gilbert-Dussardier, L. Wittler, M. Borschiwer, S. A. Haas, M. Osterwalder, M. Franke, B. Timmermann, J. Hecht, M. Spielmann, A. Visel, S. Mundlos, Disruptions of topological chromatin domains cause pathogenic rewiring of gene-enhancer interactions. *Cell* **161**, 1012–1025 (2015).

8. N. Gonen, C. R. Futtner, S. Wood, S. A. Garcia-Moreno, I. M. Salamone, S. C. Samson, R. Sekido, F. Poulat, D. M. Maatouk, R. Lovell-Badge, Sex reversal following deletion of a single distal enhancer of Sox9. *Science* **360**, 1469–1473 (2018).

9. M. T. Maurano, R. Humbert, E. Rynes, R. E. Thurman, E. Haugen, H. Wang, A. P. Reynolds, R. Sandstrom, H. Qu, J. Brody, A. Shafer, F. Neri, K. Lee, T. Kutyavin, S. Stehling-Sun, A. K. Johnson, T. K. Canfield, E. Giste, M. Diegel, D. Bates, R. S. Hansen, S. Neph, P. J. Sabo, S.

Heimfeld, A. Raubitschek, S. Ziegler, C. Cotsapas, N. Sotoodehnia, I. Glass, S. R. Sunyaev, R. Kaul, J. A. Stamatoyannopoulos, Systematic localization of common disease-associated variation in regulatory DNA. *Science* **337**, 1190–1195 (2012).

10. E. Smith, A. Shilatifard, Enhancer biology and enhanceropathies. *Nat. Struct. Mol. Biol.* **21**, 210–219 (2014).

11. A. Claringbould, J. B. Zaugg, Enhancers in disease: Molecular basis and emerging treatment strategies. *Trends Mol. Med.* **27**, 1060–1073 (2021).

12. N. A. Kearns, H. Pham, B. Tabak, R. M. Genga, N. J. Silverstein, M. Garber, R. Maehr, Functional annotation of native enhancers with a Cas9–histone demethylase fusion. *Nat. Methods* **12**, 401–403 (2015).

13. B. Bonev, N. Mendelson Cohen, Q. Szabo, L. Fritsch, G. L. Papadopoulos, Y. Lubling, X. Xu, X. Lv, J.-P. Hugnot, A. Tanay, G. Cavalli, Multiscale 3D genome rewiring during mouse neural development. *Cell* **171**, 557–572.e24 (2017).

14. Z. Chen, V. Snetkova, G. Bower, S. Jacinto, B. Clock, A. Dizehchi, I. Barozzi, B. J. Mannion, A. Alcaina-Caro, J. Lopez-Rios, D. E. Dickel, A. Visel, L. A. Pennacchio, E. Z. Kvon, Increased enhancer–promoter interactions during developmental enhancer activation in mammals. *Nat. Genet.* **56**, 675–685 (2024).

15. P. Liu, M. Chen, Y. Liu, L. S. Qi, S. Ding, CRISPR-based chromatin remodeling of the endogenous Oct4 or Sox2 locus enables reprogramming to pluripotency. *Cell Stem Cell* **22**, 252–261.e4 (2018).

16. W. W. C. Tang, A. Castillo-Venzor, W. H. Gruhn, T. Kobayashi, C. A. Penfold, M. D. Morgan, D. Sun, N. Irie, M. A. Surani, Sequential enhancer state remodelling defines human germline competence and specification. *Nat. Cell Biol.* **24**, 448–460 (2022).

17. J. Banerji, S. Rusconi, W. Schaffner, Expression of a beta-globin gene is enhanced by remote SV40 DNA sequences. *Cell* **27**, 299–308 (1981).

18. A. Visel, M. J. Blow, Z. Li, T. Zhang, J. A. Akiyama, A. Holt, I. Plajzer-Frick, M. Shoukry, C. Wright, F. Chen, V. Afzal, B. Ren, E. M. Rubin, L. A. Pennacchio, ChIP-seq accurately predicts tissue-specific activity of enhancers. *Nature* **457**, 854–858 (2009).

19. N. D. Heintzman, G. C. Hon, R. D. Hawkins, P. Kheradpour, A. Stark, L. F. Harp, Z. Ye, L. K. Lee, R. K. Stuart, C. W. Ching, K. A. Ching, J. E. Antosiewicz-Bourget, H. Liu, X. Zhang, R. D. Green, V. V. Lobanenkov, R. Stewart, J. A. Thomson, G. E. Crawford, M. Kellis, B. Ren, Histone modifications at human enhancers reflect global cell-type-specific gene expression. *Nature* **459**, 108–112 (2009).

20. M. P. Creyghton, A. W. Cheng, G. G. Welstead, T. Kooistra, B. W. Carey, E. J. Steine, J. Hanna, M. A. Lodato, G. M. Frampton, P. A. Sharp, L. A. Boyer, R. A. Young, R. Jaenisch, Histone H3K27ac separates active from poised enhancers and predicts developmental state. *Proc. Natl. Acad. Sci. U.S.A.* **107**, 21931–21936 (2010).

21. A. Rada-Iglesias, R. Bajpai, T. Swigut, S. A. Brugmann, R. A. Flynn, J. Wysocka, A unique chromatin signature uncovers early developmental enhancers in humans. *Nature* **470**, 279–283 (2011).

22. V. Sartorelli, S. M. Lauberth, Enhancer RNAs are an important regulatory layer of the epigenome. *Nat. Struct. Mol. Biol.* **27**, 521–528 (2020).

23. T. S. Barakat, F. Halbritter, M. Zhang, A. F. Rendeiro, E. Perenthaler, C. Bock, I. Chambers, Functional dissection of the enhancer repertoire in human embryonic stem cells. *Cell Stem Cell* **23**, 276–288.e8 (2018).

24. A. Visel, S. Minovitsky, I. Dubchak, L. A. Pennacchio, VISTA Enhancer Browser—A database of tissue-specific human enhancers. *Nucleic Acids Res.* **35**, D88–92 (2007).

25. D. U. Gorkin, I. Barozzi, Y. Zhao, Y. Zhang, H. Huang, A. Y. Lee, B. Li, J. Chiou, A. Wildberg, B. Ding, B. Zhang, M. Wang, J. S. Strattan, J. M. Davidson, Y. Qiu, V. Afzal, J. A. Akiyama, I. Plajzer-Frick, C. S. Novak, M. Kato, T. H. Garvin, Q. T. Pham, A. N. Harrington, B. J. Mannion, E. A. Lee, Y. Fukuda-Yuzawa, Y. He, S. Preissl, S. Chee, J. Y. Han, B. A. Williams,

D. Trout, H. Amrhein, H. Yang, J. M. Cherry, W. Wang, K. Gaulton, J. R. Ecker, Y. Shen, D. E. Dickel, A. Visel, L. A. Pennacchio, B. Ren, An atlas of dynamic chromatin landscapes in mouse fetal development. *Nature* **583**, 744–751 (2020).

26. B. J. Mannion, S. Tran, I. Plajzer-Frick, C. S. Novak, V. Afzal, J. A. Akiyama, I. Sospedra-Arrufat, S. Barton, E. Beckman, T. H. Garvin, P. Godfrey, J. Godoy, R. D. Hunter, M. Kato, M. Kosicki, A. N. Kronshage, E. A. Lee, E. M. Meky, Q. T. Pham, K. von Maydell, Y. Zhu, J. Lopez-Rios, D. E. Dickel, M. Osterwalder, A. Visel, L. A. Pennacchio, Uncovering hidden enhancers through unbiased in vivo testing. *Nat. Commun.* **16**, 7313 (2025).

27. M. Kosicki, F. A. Baltoumas, G. Kelman, J. Boverhof, Y. Ong, L. E. Cook, D. E. Dickel, G. A. Pavlopoulos, L. A. Pennacchio, A. Visel, VISTA Enhancer browser: An updated database of tissue-specific developmental enhancers. *Nucleic Acids Res.* **53**, D324–D330 (2025).

28. A. Sankar, F. Mohammad, A. K. Sundaramurthy, H. Wang, M. Lerdrup, T. Tatar, K. Helin, Histone editing elucidates the functional roles of H3K27 methylation and acetylation in mammals. *Nat. Genet.* **54**, 754–760 (2022).

29. T. Zhang, Z. Zhang, Q. Dong, J. Xiong, B. Zhu, Histone H3K27 acetylation is dispensable for enhancer activity in mouse embryonic stem cells. *Genome Biol.* **21**, 45 (2020).

30. M. M. Pradeepa, G. R. Grimes, Y. Kumar, G. Olley, G. C. A. Taylor, R. Schneider, W. A. Bickmore, Histone H3 globular domain acetylation identifies a new class of enhancers. *Nat. Genet.* **48**, 681–686 (2016).

31. N. Rajagopal, S. Srinivasan, K. Kooshesh, Y. Guo, M. D. Edwards, B. Banerjee, T. Syed, B. J. M. Emons, D. K. Gifford, R. I. Sherwood, High-throughput mapping of regulatory DNA. *Nat. Biotechnol.* **34**, 167–174 (2016).

32. Y. Diao, R. Fang, B. Li, Z. Meng, J. Yu, Y. Qiu, K. C. Lin, H. Huang, T. Liu, R. J. Marina, I. Jung, Y. Shen, K.-L. Guan, B. Ren, A tiling-deletion-based genetic screen for cis-regulatory element identification in mammalian cells. *Nat. Methods* **14**, 629–635 (2017).

33. C. D. Arnold, D. Gerlach, C. Stelzer, Ł. M. Boryń, M. Rath, A. Stark, Genome-wide quantitative enhancer activity maps identified by STARR-seq. *Science* **339**, 1074–1077 (2013).

34. J. D. Buenrostro, P. G. Giresi, L. C. Zaba, H. Y. Chang, W. J. Greenleaf, Transposition of native chromatin for fast and sensitive epigenomic profiling of open chromatin, DNA-binding proteins and nucleosome position. *Nat. Methods* **10**, 1213–1218 (2013).

35. B. Schwalb, M. Michel, B. Zacher, K. Frühauf, C. Demel, A. Tresch, J. Gagneur, P. Cramer, TT-seq maps the human transient transcriptome. *Science* **352**, 1225–1228 (2016).

36. H. S. Kaya-Okur, S. J. Wu, C. A. Codomo, E. S. Pledger, T. D. Bryson, J. G. Henikoff, K. Ahmad, S. Henikoff, CUT&Tag for efficient epigenomic profiling of small samples and single cells. *Nat. Commun.* **10**, 1930 (2019).

37. B. Mifsud, F. Tavares-Cadete, A. N. Young, R. Sugar, S. Schoenfelder, L. Ferreira, S. W. Wingett, S. Andrews, W. Grey, P. A. Ewels, B. Herman, S. Happe, A. Higgs, E. LeProust, G. A. Follows, P. Fraser, N. M. Luscombe, C. S. Osborne, Mapping long-range promoter contacts in human cells with high-resolution capture Hi-C. *Nat. Genet.* **47**, 598–606 (2015).

38. S. Schoenfelder, M. Furlan-Magaril, B. Mifsud, F. Tavares-Cadete, R. Sugar, B.-M. Javierre, T. Nagano, Y. Katsman, M. Sakthidevi, S. W. Wingett, E. Dimitrova, A. Dimond, L. B. Edelman, S. Elderkin, K. Tabbada, E. Darbo, S. Andrews, B. Herman, A. Higgs, E. LeProust, C. S. Osborne, J. A. Mitchell, N. M. Luscombe, P. Fraser, The pluripotent regulatory circuitry connecting promoters to their long-range interacting elements. *Genome Res.* **25**, 582–597 (2015).

39. C. Neumayr, M. Pagani, A. Stark, C. D. Arnold, STARR-seq and UMI-STARR-seq: Assessing enhancer activities for genome-wide-, high-, and low-complexity candidate libraries. *Curr. Protoc. Mol. Biol.* **128**, e105 (2019).

40. F. Muerdter, Ł. M. Boryń, A. R. Woodfin, C. Neumayr, M. Rath, M. A. Zabidi, M. Pagani, V. Haberle, T. Kazmar, R. R. Catarino, K. Schernhuber, C. D. Arnold, A. Stark, Resolving systematic errors in widely used enhancer activity assays in human cells. *Nat. Methods* **15**, 141–149 (2018).

41. I. Streeter, P. W. Harrison, A. Faulconbridge, HipSci Consortium, P. Flicek, H. Parkinson, L. Clarke, The human-induced pluripotent stem cell initiative—Data resources for cellular genetics. *Nucleic Acids Res.* **45**, D691–D697 (2017).

42. F.-J. Müller, B. M. Schuldt, R. Williams, D. Mason, G. Altun, E. P. Papapetrou, S. Danner, J. E. Goldmann, A. Herbst, N. O. Schmidt, J. B. Aldenhoff, L. C. Laurent, J. F. Loring, A bioinformatic assay for pluripotency in human cells. *Nat. Methods* **8**, 315–317 (2011).

43. D. Lee, M. Shi, J. Moran, M. Wall, J. Zhang, J. Liu, D. Fitzgerald, Y. Kyono, L. Ma, K. P. White, M. Gerstein, STARRPeaker: Uniform processing and accurate identification of STARR-seq active regions. *Genome Biol.* **21**, 298 (2020).

44. M. R. Corces, A. E. Trevino, E. G. Hamilton, P. G. Greenside, N. A. Sinnott-Armstrong, S. Vesuna, A. T. Satpathy, A. J. Rubin, K. S. Montine, B. Wu, A. Kathiria, S. W. Cho, M. R. Mumbach, A. C. Carter, M. Kasowski, L. A. Orloff, V. I. Risca, A. Kundaje, P. A. Khavari, T. J. Montine, W. J. Greenleaf, H. Y. Chang, An improved ATAC-seq protocol reduces background and enables interrogation of frozen tissues. *Nat. Methods* **14**, 959–962 (2017).

45. J. Eggenberger, D. Blanco-Melo, M. Panis, K. J. Brennand, B. R. tenOever, Type I interferon response impairs differentiation potential of pluripotent stem cells. *Proc. Natl. Acad. Sci. U.S.A.* **116**, 1384–1393 (2019).

46. Y. Liu, S. Yu, V. K. Dhiman, T. Brunetti, H. Eckart, K. P. White, Functional assessment of human enhancer activities using whole-genome STARR-sequencing. *Genome Biol.* **18**, 219 (2017).

47. T. Peng, Y. Zhai, Y. Atlasi, M. ter Huurne, H. Marks, H. G. Stunnenberg, W. Megchelenbrink, STARR-seq identifies active, chromatin-masked, and dormant enhancers in pluripotent mouse embryonic stem cells. *Genome Biol.* **21**, 243 (2020).

48. L. Hofbauer, L.-M. Pleyer, F. Reiter, A. Schleiffer, A. Vlasova, L. Serebreni, A. Huang, A. Stark, A genome-wide screen identifies silencers with distinct chromatin properties and mechanisms of repression. *Mol. Cell* **84**, 4503–4521.e14 (2024).

49. B. P. de Almeida, F. Reiter, M. Pagani, A. Stark, DeepSTARR predicts enhancer activity from DNA sequence and enables the de novo design of synthetic enhancers. *Nat. Genet.* **54**, 613–624 (2022).

50. I. Hossain, P. Priam, S. C. Reynoso, S. Sahni, X. X. Zhang, L. Côté, J. Doumat, C. Chik, T. Fu, J. A. Lessard, W. A. Pastor, ZIC2 and ZIC3 promote SWI/SNF recruitment to safeguard progression towards human primed pluripotency. *Nat. Commun.* **15**, 8539 (2024).

51. S. Wingett, P. Ewels, M. Furlan-Magaril, T. Nagano, S. Schoenfelder, P. Fraser, S. Andrews, HiCUP: Pipeline for mapping and processing Hi-C data. *F1000Res* **4**, 1310 (2015).

52. J. Cairns, P. Freire-Pritchett, S. W. Wingett, C. Várnai, A. Dimond, V. Plagnol, D. Zerbino, S. Schoenfelder, B.-M. Javierre, C. Osborne, P. Fraser, M. Spivakov, CHiCAGO: Robust detection of DNA looping interactions in Capture Hi-C data. *Genome Biol.* **17**, 127 (2016).

53. J. M. Alexander, J. Guan, B. Li, L. Maliskova, M. Song, Y. Shen, B. Huang, S. Lomvardas, O. D. Weiner, Live-cell imaging reveals enhancer-dependent Sox2 transcription in the absence of enhancer proximity. *eLife* **8**, e41769 (2019).

54. N. S. Benabdallah, I. Williamson, R. S. Illingworth, L. Kane, S. Boyle, D. Sengupta, G. R. Grimes, P. Therizols, W. A. Bickmore, Decreased enhancer-promoter proximity accompanying enhancer activation. *Mol. Cell* **76**, 473–484.e7 (2019).

55. S. Cruz-Molina, P. Respuela, C. Tebartz, P. Kolovos, M. Nikolic, R. Fueyo, W. F. J. van Ijcken, F. Grosveld, P. Frommolt, H. Bazzi, A. Rada-Iglesias, PRC2 facilitates the regulatory topology required for poised enhancer function during pluripotent stem cell differentiation. *Cell Stem Cell* **20**, 689–705.e9 (2017).

56. W. Xie, M. D. Schultz, R. Lister, Z. Hou, N. Rajagopal, P. Ray, J. W. Whitaker, S. Tian, R. D. Hawkins, D. Leung, H. Yang, T. Wang, A. Y. Lee, S. A. Swanson, J. Zhang, Y. Zhu, A. Kim, J. R. Nery, M. A. Urich, S. Kuan, C. Yen, S. Klugman, P. Yu, K. Suknuntha, N. E. Propson, H. Chen, L. E. Edsall, U. Wagner, Y. Li, Z. Ye, A. Kulkarni, Z. Xuan, W.-Y. Chung, N. C. Chi, J. E. Antosiewicz-Bourget, I. Slukvin, R. Stewart, M. Q. Zhang, W. Wang, J. A. Thomson, J. R.

Ecker, B. Ren, Epigenomic analysis of multilineage differentiation of human embryonic stem cells. *Cell* **153**, 1134–1148 (2013).

57. P. Chovanec, A. J. Collier, C. Krueger, C. Várnai, C. I. Semprich, S. Schoenfelder, A. E. Corcoran, P. J. Rugg-Gunn, Widespread reorganisation of pluripotent factor binding and gene regulatory interactions between human pluripotent states. *Nat. Commun.* **12**, 2098 (2021).

58. L. A. Lettice, S. J. H. Heaney, L. A. Purdie, L. Li, P. de Beer, B. A. Oostra, D. Goode, G. Elgar, R. E. Hill, E. de Graaff, A long-range Shh enhancer regulates expression in the developing limb and fin and is associated with preaxial polydactyly. *Hum. Mol. Genet.* **12**, 1725–1735 (2003).

59. C. D. Todd, J. Ijaz, F. Torabi, O. Dovgusha, S. Bevan, O. Cracknell, T. Lohoff, S. Clark, R. Argelaguet, J. Pierce, I. Kafetzopoulos, A. Santambrogio, J. Nichols, F. von Meyenn, U. Günesdogan, S. Schoenfelder, W. Reik, Epigenetic priming of mammalian embryonic enhancer elements coordinates developmental gene networks. *Genome Biol.* **26**, 214 (2025).

60. A. M. Miangolarra, J. Kurlovich, F. Torabi, O. Dovgusha, Y. Cao, L. Lin, U. Günesdogan, S. Schoenfelder, M. Howard, Signatures of digital Polycomb regulation in functional iPSC heterogeneity between individuals. bioRxiv 666753 [Preprint] (2026). https://doi.org/10.1101/2025.07.25.666753.

61. Ž. Avsec, N. Latysheva, J. Cheng, G. Novati, K. R. Taylor, T. Ward, C. Bycroft, L. Nicolaisen, E. Arvaniti, J. Pan, R. Thomas, V. Dutordoir, M. Perino, S. De, A. Karollus, A. Gayoso, T. Sargeant, A. Mottram, L. H. Wong, P. Drotár, A. Kosiorek, A. Senior, R. Tanburn, T. Applebaum, S. Basu, D. Hassabis, P. Kohli, Advancing regulatory variant effect prediction with AlphaGenome. *Nature* **649**, 1206–1218 (2026).

62. C. P. Fulco, M. Munschauer, R. Anyoha, G. Munson, S. R. Grossman, E. M. Perez, M. Kane, B. Cleary, E. S. Lander, J. M. Engreitz, Systematic mapping of functional enhancer-promoter connections with CRISPR interference. *Science* **354**, 769–773 (2016).

63. M. Gasperini, A. J. Hill, J. L. McFaline-Figueroa, B. Martin, S. Kim, M. D. Zhang, D. Jackson, A. Leith, J. Schreiber, W. S. Noble, C. Trapnell, N. Ahituv, J. Shendure, A genome-wide

framework for mapping gene regulation via cellular genetic screens. *Cell* **176**, 377–390.e19 (2019).

64. D. Schraivogel, A. R. Gschwind, J. H. Milbank, D. R. Leonce, P. Jakob, L. Mathur, J. O. Korbel, C. A. Merten, L. Velten, L. M. Steinmetz, Targeted Perturb-seq enables genome-scale genetic screens in single cells. *Nat. Methods* **17**, 629–635 (2020).

65. T. Narita, Y. Higashijima, S. Kilic, T. Liebner, J. Walter, C. Choudhary, Acetylation of histone H2B marks active enhancers and predicts CBP/p300 target genes. *Nat. Genet.* **55**, 679–692 (2023).

66. M. M. Pradeepa, Causal role of histone acetylations in enhancer function. *Transcription* **8**, 40–47 (2017).

67. G. E. Zentner, P. J. Tesar, P. C. Scacheri, Epigenetic signatures distinguish multiple classes of enhancers with distinct cellular functions. *Genome Res.* **21**, 1273–1283 (2011).

68. D. A. Braun, C. E. Sadowski, S. Kohl, S. Lovric, S. A. Astrinidis, W. L. Pabst, H. Y. Gee, S. Ashraf, J. A. Lawson, S. Shril, M. Airik, W. Tan, D. Schapiro, J. Rao, W.-I. Choi, T. Hermle, M. J. Kemper, M. Pohl, F. Ozaltin, M. Konrad, R. Bogdanovic, R. Büscher, U. Helmchen, E. Serdaroglu, R. P. Lifton, W. Antonin, F. Hildebrandt, Mutations in nuclear pore genes NUP93, NUP205 and XPO5 cause steroid-resistant nephrotic syndrome. *Nat. Genet.* **48**, 457–465 (2016).

69. W. Chen, Y. Zhang, S. Yang, Z. Shi, W. Zeng, Z. Lu, X. Zhou, Bi-allelic mutations in NUP205 and NUP210 are associated with abnormal cardiac left-right patterning. *Circ. Genom. Precis. Med.* **12**, e002492 (2019).

70. P. A. Dawson, J. Rakoczy, D. G. Simmons, Placental, renal, and ileal sulfate transporter gene expression in mouse gestation. *Biol. Reprod.* **87**, 43 (2012).

71. D. G. Simmons, J. Rakoczy, J. Jefferis, R. Lourie, H. D. McIntyre, P. A. Dawson, Human placental sulfate transporter mRNA profiling from term pregnancies identifies abundant *SLC13A4* in syncytiotrophoblasts and *SLC26A2* in cytotrophoblasts. *Placenta* **34**, 381–384 (2013).

72. T. J. Harvey, R. A. Davila, D. Vidovic, S. Sharmin, M. Piper, D. G. Simmons, Genome-wide transcriptomic analysis of the forebrain of postnatal *Slc13a4*$^{+/-}$ mice. *BMC. Res. Notes* **14**, 269 (2021).

73. A. Yilmaz, M. Peretz, A. Aharony, I. Sagi, N. Benvenisty, Defining essential genes for human pluripotent stem cells by CRISPR-Cas9 screening in haploid cells. *Nat. Cell Biol.* **20**, 610–619 (2018).

74. A. Eberharter, P. B. Becker, Histone acetylation: A switch between repressive and permissive chromatin. *EMBO Rep.* **3**, 224–229 (2002).

75. S. J. Hogg, O. Motorna, L. A. Cluse, T. M. Johanson, H. D. Coughlan, R. Raviram, R. M. Myers, M. Costacurta, I. Todorovski, L. Pijpers, S. Bjelosevic, T. Williams, S. N. Huskins, C. J. Kearney, J. R. Devlin, Z. Fan, J. S. Jabbari, B. P. Martin, M. Fareh, M. J. Kelly, D. Dupéré-Richer, J. J. Sandow, B. Feran, D. Knight, T. Khong, A. Spencer, S. J. Harrison, G. Gregory, V. O. Wickramasinghe, A. I. Webb, P. C. Taberlay, K. D. Bromberg, A. Lai, A. T. Papenfuss, G. K. Smyth, R. S. Allan, J. D. Licht, D. A. Landau, O. Abdel-Wahab, J. Shortt, S. J. Vervoort, R. W. Johnstone, Targeting histone acetylation dynamics and oncogenic transcription by catalytic P300/CBP inhibition. *Mol. Cell* **81**, 2183–2200.e13 (2021).

76. A. R. Pengelly, Ö. Copur, H. Jäckle, A. Herzig, J. Müller, A histone mutant reproduces the phenotype caused by loss of histone-modifying factor Polycomb. *Science* **339**, 698–699 (2013).

77. J. E. Phillips-Cremins, V. G. Corces, Chromatin insulators: Linking genome organization to cellular function. *Mol. Cell* **50**, 461–474 (2013).

78. N. Kubo, H. Ishii, X. Xiong, S. Bianco, F. Meitinger, R. Hu, J. D. Hocker, M. Conte, D. Gorkin, M. Yu, B. Li, J. R. Dixon, M. Hu, M. Nicodemi, H. Zhao, B. Ren, Promoter-proximal CTCF binding promotes distal enhancer-dependent gene activation. *Nat. Struct. Mol. Biol.* **28**, 152–161 (2021).

79. N. Q. Liu, M. Magnitov, M. M. G. A. Schijns, T. van Schaik, H. Teunissen, B. van Steensel, E. de Wit, Extrusion fountains are restricted by WAPL-dependent cohesin release and CTCF barriers. *Nucleic Acids Res.* **53**, gkaf549 (2025).

80. N. J. Rinzema, K. Sofiadis, S. J. D. Tjalsma, M. J. A. M. Verstegen, Y. Oz, C. Valdes-Quezada, A.-K. Felder, T. Filipovska, S. van der Elst, Z. de Andrade dos Ramos, R. Han, P. H. L. Krijger, W. de Laat, Building regulatory landscapes reveals that an enhancer can recruit cohesin to create contact domains, engage CTCF sites and activate distant genes. *Nat. Struct. Mol. Biol.* **29**, 563–574 (2022).

81. T. Narita, S. Kilic, Y. Higashijima, N. M. Scherer, G. Pappas, E. Maskey, C. Choudhary, Disentangling the architectural and non-architectural functions of CTCF and cohesin in gene regulation. *Nat. Genet.* **57**, 3137–3151 (2025).

82. L. Braccioli, E. de Wit, CTCF: A Swiss-army knife for genome organization and transcription regulation. *Essays Biochem.* **63**, 157–165 (2019).

83. J. Dubois-Chevalier, F. Oger, H. Dehondt, F. F. Firmin, C. Gheeraert, B. Staels, P. Lefebvre, J. Eeckhoute, A dynamic CTCF chromatin binding landscape promotes DNA hydroxymethylation and transcriptional induction of adipocyte differentiation. *Nucleic Acids Res.* **42**, 10943–10959 (2014).

84. G. Ren, W. Jin, K. Cui, J. Rodrigez, G. Hu, Z. Zhang, D. R. Larson, K. Zhao, CTCF-mediated enhancer-promoter interaction is a critical regulator of cell-to-cell variation of gene expression. *Mol. Cell* **67**, 1049–1058.e6 (2017).

85. J. Ray, E. Jagoda, M. U. Sheth, J. Galante, D. Amgalan, A. R. Gschwind, C. J. Munger, J. Huang, G. Munson, M. Murphy, E. Mattei, T. Barry, V. Singh, A. Baskaran, H. Kang, E. Katsevich, L. M. Steinmetz, J. M. Engreitz, A systematic survey of distal element-gene regulatory interactions with Direct-Capture Targeted Perturb-seq. bioRxiv 676677 [Preprint] (2025). https://doi.org/10.1101/2025.09.16.676677.

86. O. Mikhaylichenko, V. Bondarenko, D. Harnett, I. E. Schor, M. Males, R. R. Viales, E. E. M. Furlong, The degree of enhancer or promoter activity is reflected by the levels and directionality of eRNA transcription. *Genes Dev.* **32**, 42–57 (2018).

87. F. Inoue, M. Kircher, B. Martin, G. M. Cooper, D. M. Witten, M. T. McManus, N. Ahituv, J. Shendure, A systematic comparison reveals substantial differences in chromosomal versus episomal encoding of enhancer activity. *Genome Res.* **27**, 38–52 (2017).

88. M. A. Zabidi, C. D. Arnold, K. Schernhuber, M. Pagani, M. Rath, O. Frank, A. Stark, Enhancer-core-promoter specificity separates developmental and housekeeping gene regulation. *Nature* **518**, 556–559 (2015).

89. B. Sahu, T. Hartonen, P. Pihlajamaa, B. Wei, K. Dave, F. Zhu, E. Kaasinen, K. Lidschreiber, M. Lidschreiber, C. O. Daub, P. Cramer, T. Kivioja, J. Taipale, Sequence determinants of human gene regulatory elements. *Nat. Genet.* **54**, 283–294 (2022).

90. R. M. Boileau, K. X. Chen, R. Blelloch, Loss of MLL3/4 decouples enhancer H3K4 monomethylation, H3K27 acetylation, and gene activation during embryonic stem cell differentiation. *Genome Biol.* **24**, 41 (2023).

91. I. Olan, M. Ando-Kuri, A. J. Parry, T. Handa, S. Schoenfelder, P. Fraser, Y. Ohkawa, H. Kimura, M. Narita, M. Narita, HMGA1 orchestrates chromatin compartmentalization and sequesters genes into 3D networks coordinating senescence heterogeneity. *Nat. Commun.* **15**, 6891 (2024).

92. H. Vlaming, C. A. Mimoso, A. R. Field, B. J. E. Martin, K. Adelman, Screening thousands of transcribed coding and non-coding regions reveals sequence determinants of RNA polymerase II elongation potential. *Nat. Struct. Mol. Biol.* **29**, 613–620 (2022).

93. A. D. Bell, C. L. Usher, S. A. McCarroll, Analyzing copy number variation with droplet digital PCR. *Methods Mol. Biol.* **1768**, 143–160 (2018).

94. A. Dobin, C. A. Davis, F. Schlesinger, J. Drenkow, C. Zaleski, S. Jha, P. Batut, M. Chaisson, T. R. Gingeras, STAR: Ultrafast universal RNA-seq aligner. *Bioinformatics* **29**, 15–21 (2013).

95. S. Anders, P. T. Pyl, W. Huber, HTSeq—A Python framework to work with high-throughput sequencing data. *Bioinformatics* **31**, 166–169 (2015).

96. M. I. Love, W. Huber, S. Anders, Moderated estimation of fold change and dispersion for RNA-seq data with DESeq2. *Genome Biol.* **15**, 550 (2014).

97. H. Wickham, *ggplot2: Elegant Graphics for Data Analysis* (Springer, Switzerland, ed. 1, 2009).

98. G. Yu, L.-G. Wang, Y. Han, Q.-Y. He, clusterProfiler: An R package for comparing biological themes among gene clusters. *OMICS* **16**, 284–287 (2012).

99. F. Ramírez, D. P. Ryan, B. Grüning, V. Bhardwaj, F. Kilpert, A. S. Richter, S. Heyne, F. Dündar, T. Manke, deepTools2: A next generation web server for deep-sequencing data analysis. *Nucleic Acids Res.* **44**, W160–W165 (2016).

100. M. Martin, Cutadapt removes adapter sequences from high-throughput sequencing reads. *EMBnet J.* **17**, 10–12 (2011).

101. B. Langmead, S. L. Salzberg, Fast gapped-read alignment with Bowtie 2. *Nat. Methods* **9**, 357–359 (2012).

102. H. Li, B. Handsaker, A. Wysoker, T. Fennell, J. Ruan, N. Homer, G. Marth, G. Abecasis, R. Durbin, 1000 Genome Project Data Processing Subgroup, The Sequence Alignment/Map format and SAMtools. *Bioinformatics* **25**, 2078–2079 (2009).

103. H. M. Amemiya, A. Kundaje, A. P. Boyle, The ENCODE Blacklist: Identification of problematic regions of the genome. *Sci. Rep.* **9**, 9354 (2019).

104. J. Feng, T. Liu, B. Qin, Y. Zhang, X. S. Liu, Identifying ChIP-seq enrichment using MACS. *Nat. Protoc.* **7**, 1728–1740 (2012).

105. R. C. McLeay, T. L. Bailey, Motif Enrichment Analysis: A unified framework and an evaluation on ChIP data. *BMC Bioinformatics* **11**, 165 (2010).

106. T. L. Bailey, J. Johnson, C. E. Grant, W. S. Noble, The MEME suite. *Nucleic Acids Res.* **43**, W39–W49 (2015).
